# Supplementary material for: Linked-read sequencing of gametes allows efficient genome-wide analysis of meiotic recombination
Source: Nat Commun. 2019 Sep 20;10:4310. doi: 10.1038/s41467-019-12209-2 (PMC6754367; doi:10.1038/s41467-019-12209-2)
Supplement: Supplementary file 3 — Reporting Summary [file 41467_2019_12209_MOESM3_ESM.pdf]

nature research

Corresponding author(s): Beth Rowan, Korbinian Schneeberger

Last updated by author(s): Jul 22, 2019

Reporting Summary

Nature Research wishes to improve the reproducibility of the work that we publish. This form provides structure for consistency and transparency in reporting. For further information on Nature Research policies, see Authors & Referees and the Editorial Policy Checklist.

Statistics

For all statistical analyses, confirm that the following items are present in the figure legend, table legend, main text, or Methods section.

n/a	Confirmed	
☐	☒	The exact sample size (*n*) for each experimental group/condition, given as a discrete number and unit of measurement
☐	☒	A statement on whether measurements were taken from distinct samples or whether the same sample was measured repeatedly
☐	☒	The statistical test(s) used AND whether they are one- or two-sided<br>*Only common tests should be described solely by name; describe more complex techniques in the Methods section.*
☒	☐	A description of all covariates tested
☐	☒	A description of any assumptions or corrections, such as tests of normality and adjustment for multiple comparisons
☐	☒	A full description of the statistical parameters including central tendency (e.g. means) or other basic estimates (e.g. regression coefficient) AND variation (e.g. standard deviation) or associated estimates of uncertainty (e.g. confidence intervals)
☐	☒	For null hypothesis testing, the test statistic (e.g. *F*, *t*, *r*) with confidence intervals, effect sizes, degrees of freedom and *P* value noted<br>*Give P values as exact values whenever suitable.*
☒	☐	For Bayesian analysis, information on the choice of priors and Markov chain Monte Carlo settings
☒	☐	For hierarchical and complex designs, identification of the appropriate level for tests and full reporting of outcomes
☐	☒	Estimates of effect sizes (e.g. Cohen's *d*, Pearson's *r*), indicating how they were calculated

Our web collection on statistics for biologists contains articles on many of the points above.

Software and code

Policy information about availability of computer code

Data collection	No code used for data collection.
Data analysis	The following tools were used in data analysis. Binary code: longranger (v2.2.2). Open source code: Bowtie2 (version 2.2.8), SAMtools/BCFtools44 (version 1.3.1), TIGER (version 1.0). R code for t-test, correlation test, Kolmogorov-Smirnov test and permutation test. Custom C/C++ code (including DrLink v1.0 at https://github.com/schneebergerlab/DrLink) for CO detection (molecule recovery and genotyping), relative recombination frequency estimation, recombination chromosomal distribution, GC content calculation, GFF (including TE) feature association, DNA methylation level estimation.

For manuscripts utilizing custom algorithms or software that are central to the research but not yet described in published literature, software must be made available to editors/reviewers. We strongly encourage code deposition in a community repository (e.g. GitHub). See the Nature Research guidelines for submitting code & software for further information.

Data

Policy information about availability of data

All manuscripts must include a data availability statement. This statement should provide the following information, where applicable:

- Accession codes, unique identifiers, or web links for publicly available datasets
- A list of figures that have associated raw data
- A description of any restrictions on data availability

Read data of all eight 10X linked-read libraries (ERS2851779-ERS2851786) and 50 whole genome sequencing libraries (ERS2851943-ERS2851992) that support the findings of this study are available in BAM format from European Nucleotide Archive under accession number ERP111558.

Field-specific reporting

Please select the one below that is the best fit for your research. If you are not sure, read the appropriate sections before making your selection.

☒ Life sciences ☐ Behavioural & social sciences ☐ Ecological, evolutionary & environmental sciences

For a reference copy of the document with all sections, see nature.com/documents/nr-reporting-summary-flat.pdf

Life sciences study design

All studies must disclose on these points even when the disclosure is negative.

Sample size

Simulations, regarding uniqueness of predicted COs and pool size of Col X Ler F2s which followed a Poisson distribution, were used to predetermine the sample size. Details below were also provided in the supplementary data of the submission.

To estimate the effects of varying numbers of distinct, haploid genomes G (pool size), we first assumed a total number of distinct CO events N, and a recombinant molecule number n. N was set as N=G * 4.15 (i.e. the average number of COs per haploid genome). For the recombinant molecule number, we estimated the probability of a molecule to cover a CO as 10 kb * 4.15 / 120Mb (=3.33e-04), assuming a core molecule length (region in which a CO can be identified) of 10 kb, 4.15 CO events per haploid genome and a haploid genome size of 120 Mb. Further assuming a 10X library with 10 million molecules, we finally estimated a recombinant molecule number n of 3,333 per library. Based on N and n, the average molecule coverage across all distinct CO events would be lamda=n/N. Thus, the expected number of COs covered by k molecules (k=0, 1, 2, …, n) would be N * e^{-lamda} * lamda^k / k! . Using all the above, we investigated the effect of G on the molecule coverages in a pool. While increasing the pool size can always increase the uniqueness of predicted COs, the pool size needs to increase by ~1000 to reach 90% uniqueness as compared to a pool size of 1250 with 85% uniqueness (supplementary Table S6), which becomes impractically trivial.

Thus, based on simulations, three F2 sample sizes of 50, 200, and 1250 were selected. The small pool with the 50 F2s with 400 expected COs was sufficient and practical to validate the method using individual whole genome sequencings (WGS). Larger pools of 200 and 1250 F2 were used to investigate the effect of pool size on uniqueness of predicted COs in practice, and compare with COs detected within a different population of Col x Ler F2s using WGS.

Data exclusions

As for the benchmark data, 415 COs were initially detected in the 50 individual F2s by TIGER pipeline, while 15 COs were excluded because they could not be detected as there were very low marker density (Supplementary Table S2: marker-inter distance over 10 kb).

Replication

For each larger pool of P200 and P1250, two replicates were created. The detections from the replicates were consistent to each other.

Randomization

All samples were randomly sampled.

Blinding

The investigators were not blinded to group allocation during data collection and data analysis. The investigators joined experimental design, and all allocated groups were finally merged as one large pool (P1250). The grouping targeted to keep all individual DNA represented as equal as possible in the final pool for sequencing.

Reporting for specific materials, systems and methods

We require information from authors about some types of materials, experimental systems and methods used in many studies. Here, indicate whether each material, system or method listed is relevant to your study. If you are not sure if a list item applies to your research, read the appropriate section before selecting a response.

Materials & experimental systems

n/a	Involved in the study
☒	☐ Antibodies
☒	☐ Eukaryotic cell lines
☒	☐ Palaeontology
☒	☐ Animals and other organisms
☒	☐ Human research participants
☒	☐ Clinical data

Methods

n/a	Involved in the study
☒	☐ ChIP-seq
☒	☐ Flow cytometry
☒	☐ MRI-based neuroimaging
