# Supplementary material for: Linked-read sequencing of gametes allows efficient genome-wide analysis of meiotic recombination
Source: Nat Commun. 2019 Sep 20;10:4310. doi: 10.1038/s41467-019-12209-2 (PMC6754367; doi:10.1038/s41467-019-12209-2)
Supplement: Supplementary file 4 — Description of Additional Supplementary Files [file 41467_2019_12209_MOESM4_ESM.pdf]

Description of Additional Supplementary Files

Filename: Supplementary_Data_1.xlsx
Description: Sequencing summary

Filename: Supplementary_Data_2.xlsx
Description: CO benchmark dataset

Filename: Supplementary_Data_3.xlsx
Description: Criteria for CO molecule filtering

Filename: Supplementary_Data_4.xlsx
Description: Col-0 and Ler allelic markers

Filename: Supplementary_Data_5.xlsx
Description: Random overlapping of independent COs

Filename: Supplementary_Data_6.xlsx
Description: Molecule coverage per CO for various pool size

Filename: Supplementary_Data_7.xlsx
Description: Comparison on costs of different technologies
