# Supplementary material for: Linked-read sequencing of gametes allows efficient genome-wide analysis of meiotic recombination
Source: Nat Commun. 2019 Sep 20;10:4310. doi: 10.1038/s41467-019-12209-2 (PMC6754367; doi:10.1038/s41467-019-12209-2)
Supplement: Supplementary file 12 — Source Data [file 41467_2019_12209_MOESM12_ESM.zip › Source_Data/Source_Data_supplementary_Figure_1.zip!/Source_Data_supplementary_Figure_1/supplementary_Figure_1_3358_ABCDE_before_BluePippin.pdf]

Filename: 2017-12-06-01-3358_A_B_C_D_E-3363_M_O_P_Q.gDNA

Gel Image

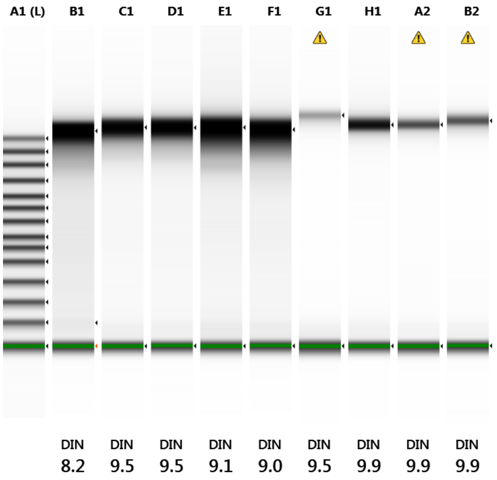

Default image (Contrast 100%)

Sample Info

Well	DIN	Conc. [ng/µl]	Sample Description	Alert	Observations
A1	-	64.1	Ladder		Ladder
B1	8.2	55.8	3358.A1 before Blue Pippin		
C1	9.5	36.7	3358.B1 before Blue Pippin		
D1	9.5	42.3	3358.C1 before Blue Pippin		
E1	9.1	59.4	3358.D1 before Blue Pippin		
F1	9.0	52.9	3358.E1 before Blue Pippin		
G1	9.5	3.96	3363.M2 after Blue Pippin	⚠	Sample concentration outside functional range for DIN
H1	9.9	18.2	3363.O2 after Blue Pippin		
A2	9.9	8.83	3363.P2 after Blue Pippin	⚠	Sample concentration outside recommended range
B2	9.9	8.39	3363.Q2 after Blue Pippin	⚠	Sample concentration outside recommended range

 © Agilent Technologies, Inc. 2016

A1: Ladder

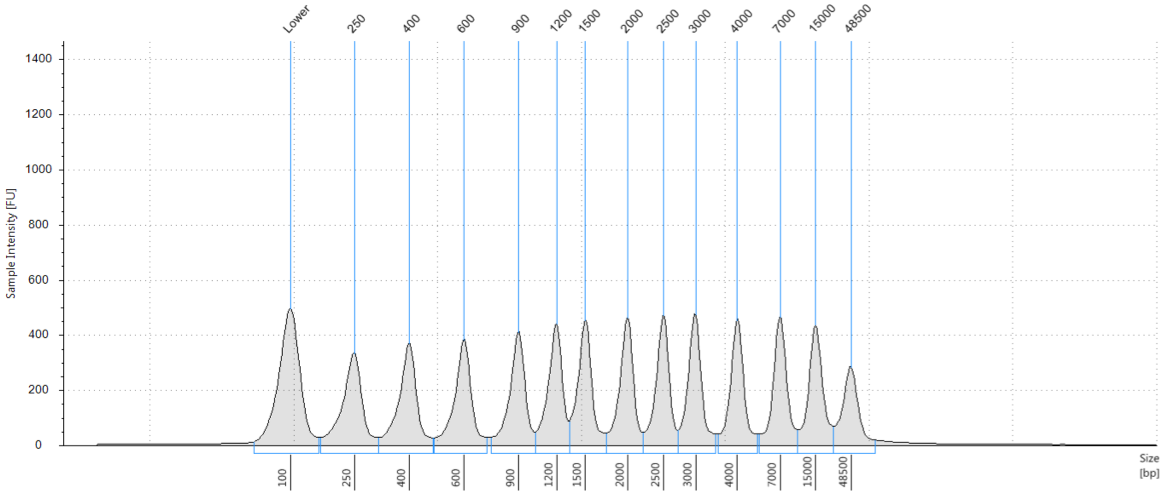

Sample Table

Well	DIN	Conc. [ng/µl]	Sample Description	Alert	Observations
A1	-	64.1	Ladder		Ladder

Peak Table

Size [bp]	Calibrated Conc. [ng/µl]	Assigned Conc. [ng/µl]	% Integrated Area	From [bp]	To [bp]	Peak Comment	Observations
100	8.50	8.50	-	59	150		Lower Marker
250	5.07	-	7.92	153	307		
400	5.09	-	7.94	307	477		
600	4.99	-	7.80	481	709		
900	5.01	-	7.83	735	1021		
1200	5.06	-	7.90	1021	1329		
1500	5.14	-	8.03	1329	1732		
2000	5.04	-	7.87	1732	2203		
2500	5.02	-	7.83	2203	2711		
3000	5.13	-	8.01	2711	3450		
4000	4.88	-	7.62	3507	5210		
7000	5.05	-	7.88	5292	10247		
15000	4.94	-	7.71	10247	21960		
48500	3.53	-	5.51	21960	>60000		
-	-	-	-	-	-		Sample Well

 © Agilent Technologies, Inc. 2016

B1: 3358.A1 before Blue Pippin

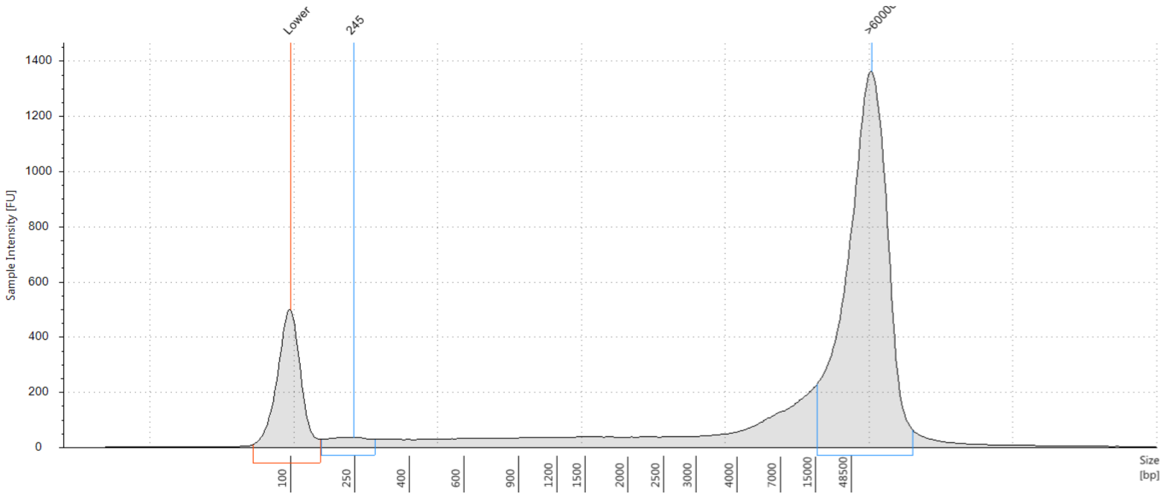

Sample Table

Well	DIN	Conc. [ng/µl]	Sample Description	Alert	Observations
B1	8.2	55.8	3358.A1 before Blue Pippin		

Peak Table

Size [bp]	Calibrated Conc. [ng/µl]	Assigned Conc. [ng/µl]	% Integrated Area	From [bp]	To [bp]	Peak Comment	Observations
100	8.50	8.50	-	58	153		Lower Marker
245	1.03	-	2.46	155	297		
>60000	40.2	-	95.88	15445	>60000		
-	-	-	-	-	-		Sample Well

 © Agilent Technologies, Inc. 2016

C1: 3358.B1 before Blue Pippin

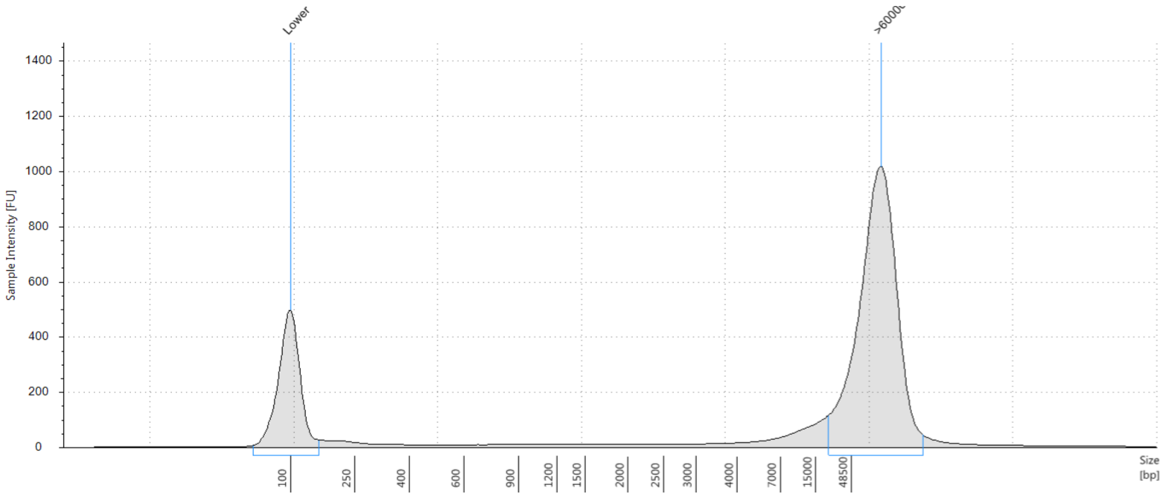

Sample Table

Well	DIN	Conc. [ng/µl]	Sample Description	Alert	Observations
C1	9.5	36.7	3358.B1 before Blue Pippin		

Peak Table

Size [bp]	Calibrated Conc. [ng/µl]	Assigned Conc. [ng/µl]	% Integrated Area	From [bp]	To [bp]	Peak Comment	Observations
100	8.50	8.50	-	59	150		Lower Marker
>60000	30.4	-	97.79	19544	>60000		
-	-	-	-	-	-		Sample Well

 © Agilent Technologies, Inc. 2016

D1: 3358.C1 before Blue Pippin

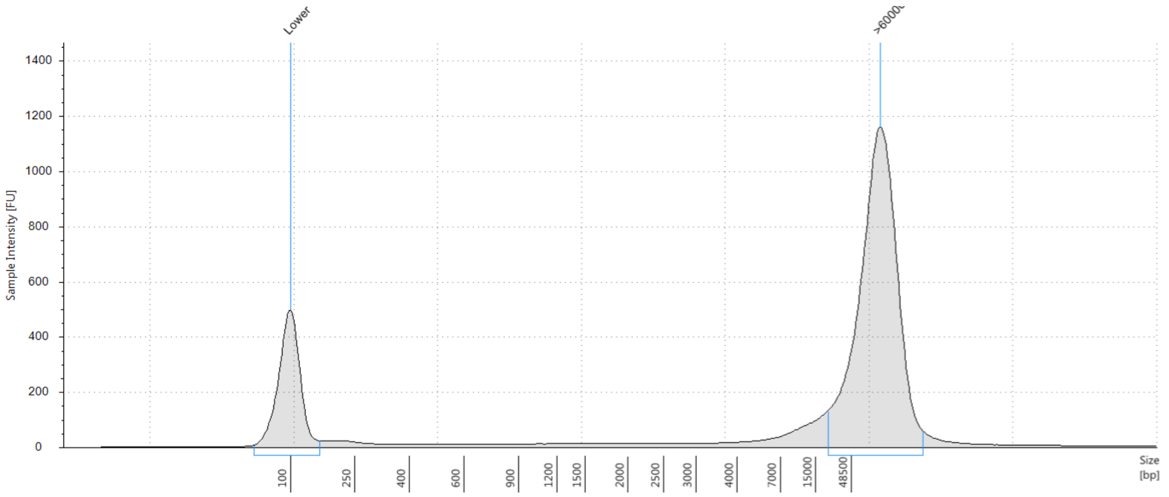

Sample Table

Well	DIN	Conc. [ng/µl]	Sample Description	Alert	Observations
D1	9.5	42.3	3358.C1 before Blue Pippin		

Peak Table

Size [bp]	Calibrated Conc. [ng/µl]	Assigned Conc. [ng/µl]	% Integrated Area	From [bp]	To [bp]	Peak Comment	Observations
100	8.50	8.50	-	59	150		Lower Marker
>60000	34.8	-	97.97	19311	>60000		
-	-	-	-	-	-		Sample Well

 © Agilent Technologies, Inc. 2016

E1: 3358.D1 before Blue Pippin

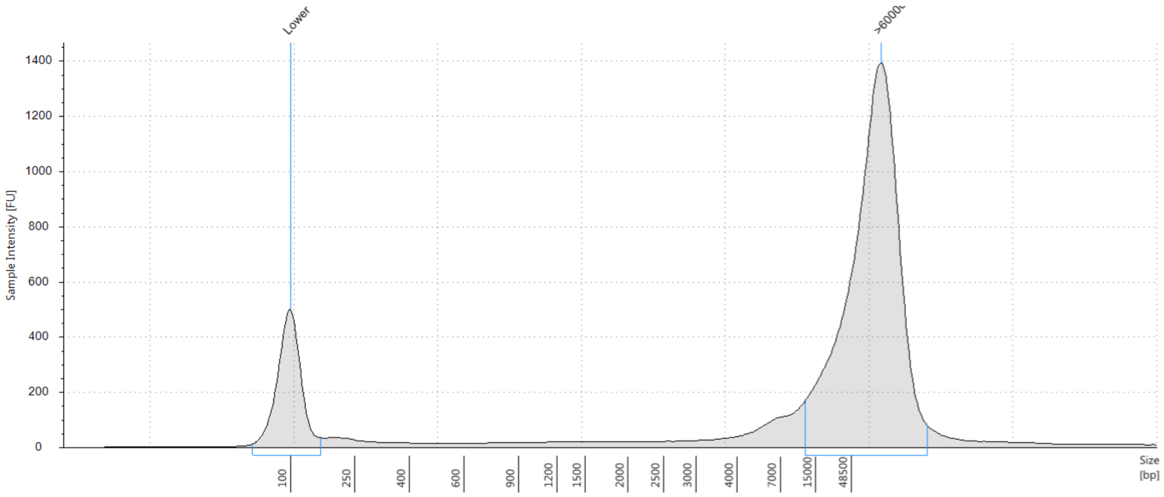

Sample Table

Well	DIN	Conc. [ng/µl]	Sample Description	Alert	Observations
E1	9.1	59.4	3358.D1 before Blue Pippin		

Peak Table

Size [bp]	Calibrated Conc. [ng/µl]	Assigned Conc. [ng/µl]	% Integrated Area	From [bp]	To [bp]	Peak Comment	Observations
100	8.50	8.50	-	58	153		Lower Marker
>60000	48.5	-	96.50	11798	>60000		
-	-	-	-	-	-		Sample Well

 © Agilent Technologies, Inc. 2016

F1: 3358.E1 before Blue Pippin

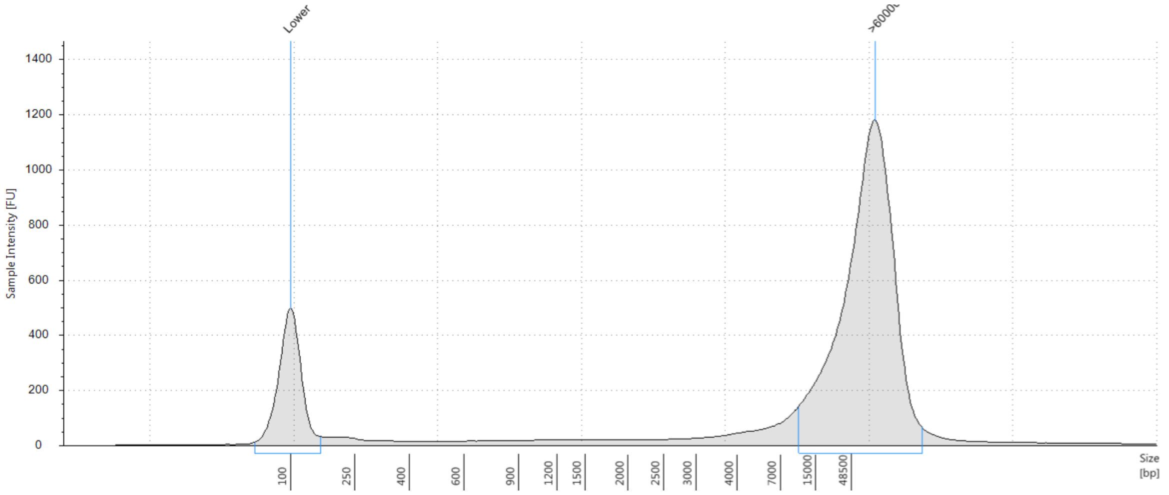

Sample Table

Well	DIN	Conc. [ng/µl]	Sample Description	Alert	Observations
F1	9.0	52.9	3358.E1 before Blue Pippin		

Peak Table

Size [bp]	Calibrated Conc. [ng/µl]	Assigned Conc. [ng/µl]	% Integrated Area	From [bp]	To [bp]	Peak Comment	Observations
100	8.50	8.50	-	59	152		Lower Marker
>60000	43.7	-	96.25	10244	>60000		
-	-	-	-	-	-		Sample Well

 © Agilent Technologies, Inc. 2016
