# Supplementary material for: Linked-read sequencing of gametes allows efficient genome-wide analysis of meiotic recombination
Source: Nat Commun. 2019 Sep 20;10:4310. doi: 10.1038/s41467-019-12209-2 (PMC6754367; doi:10.1038/s41467-019-12209-2)
Supplement: Supplementary file 12 — Source Data [file 41467_2019_12209_MOESM12_ESM.zip › Source_Data/Source_Data_supplementary_Figure_1.zip!/Source_Data_supplementary_Figure_1/supplemenrary_Figure_1_3358_DE_after_BluePippin.pdf]

Filename: 2017-12-15-3344_B1_3358.gDNA

Gel Image

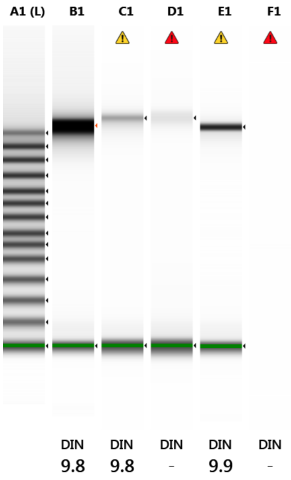

Default image (Contrast 100%)

Sample Info

Well	DIN	Conc. [ng/µl]	Sample Description	Alert	Observations
A1	-	89.4	Ladder		Ladder
B1	9.8	45.5	3344.A.2		
C1	9.8	4.78	3358.D.2		Sample concentration outside functional range for DIN
D1	-	2.66	3358.E.2		Sample concentration outside functional range for DIN
E1	9.9	9.72	Qubit HS Standard #2		Sample concentration outside recommended range
F1	-				Marker(s) not detected

 © Agilent Technologies, Inc. 2016

A1: Ladder

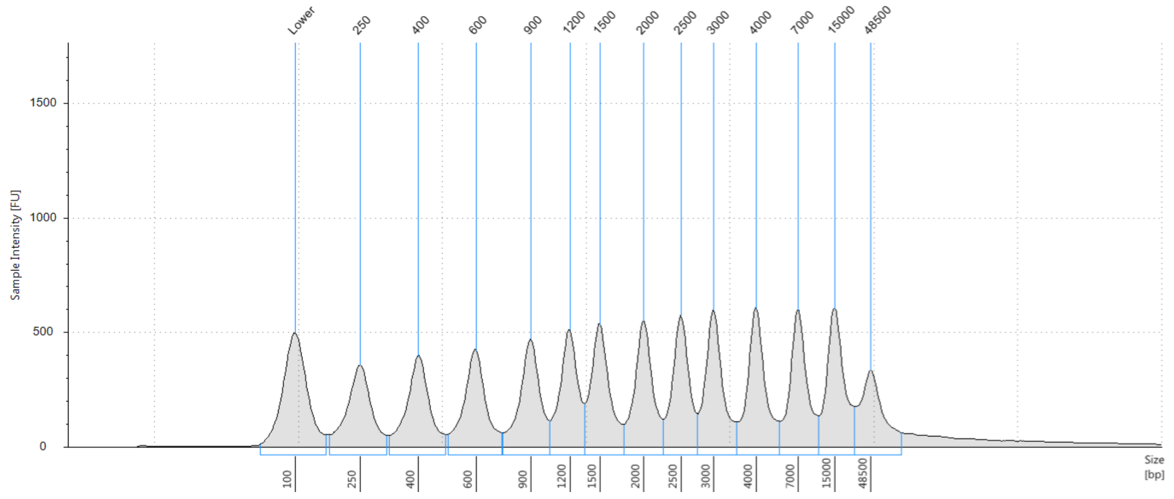

Sample Table

Well	DIN	Conc. [ng/µl]	Sample Description	Alert	Observations
A1	-	89.4	Ladder		Ladder

Peak Table

Size [bp]	Calibrated Conc. [ng/µl]	Assigned Conc. [ng/µl]	% Integrated Area	From [bp]	To [bp]	Peak Comment	Observations
100	8.50	8.50	-	61	154		Lower Marker
250	5.92	-	6.80	162	310		
400	6.12	-	7.03	316	484		
600	6.28	-	7.21	492	725		
900	6.53	-	7.50	732	1035		
1200	6.53	-	7.51	1035	1342		
1500	6.95	-	7.98	1342	1752		
2000	6.96	-	7.99	1752	2252		
2500	6.72	-	7.72	2252	2739		
3000	7.24	-	8.31	2739	3492		
4000	7.43	-	8.54	3492	5459		
7000	7.13	-	8.19	5459	10632		
15000	7.45	-	8.56	10632	22790		
48500	5.25	-	6.04	22790	>60000		
-	-	-	-	-	-		Sample Well

 © Agilent Technologies, Inc. 2016

C1: 3358.D.2

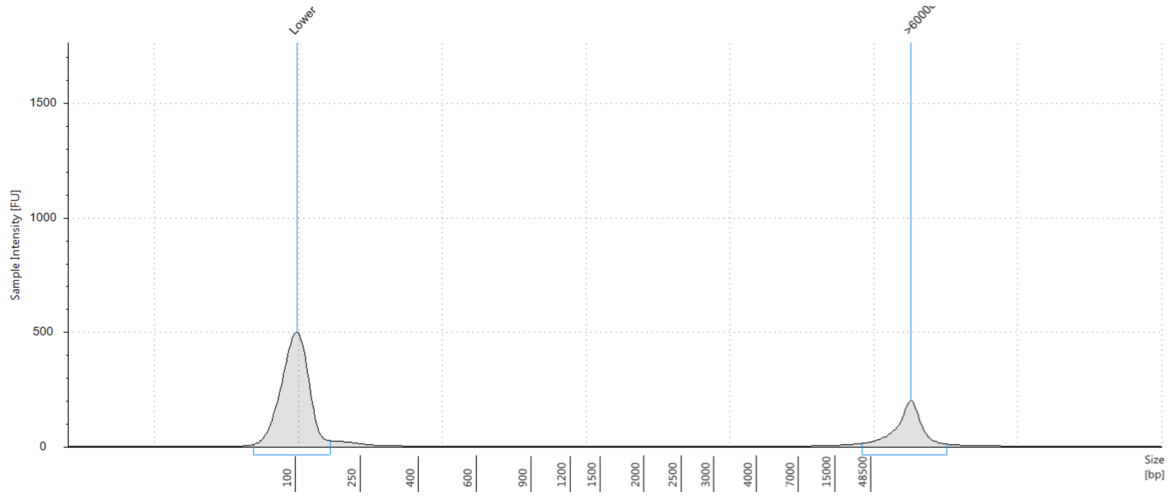

Sample Table

Well	DIN	Conc. [ng/µl]	Sample Description	Alert	Observations
C1	9.8	4.78	3358.D.2	⚠	Sample concentration outside functional range for DIN

Peak Table

Size [bp]	Calibrated Conc. [ng/µl]	Assigned Conc. [ng/µl]	% Integrated Area	From [bp]	To [bp]	Peak Comment	Observations
100	8.50	8.50	-	54	160		Lower Marker
>60000	3.11	-	100.00	27106	>60000		

 © Agilent Technologies, Inc. 2016

D1: 3358.E.2

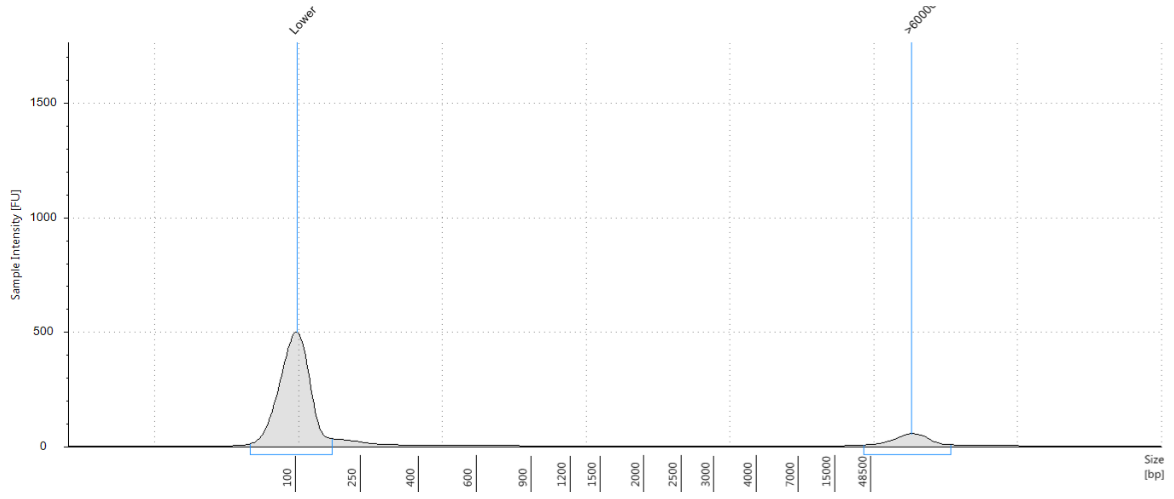

Sample Table

Well	DIN	Conc. [ng/µl]	Sample Description	Alert	Observations
D1	-	2.66	3358.E.2	⚠	Sample concentration outside functional range for DIN

Peak Table

Size [bp]	Calibrated Conc. [ng/µl]	Assigned Conc. [ng/µl]	% Integrated Area	From [bp]	To [bp]	Peak Comment	Observations
100	8.50	8.50	-	51	165		Lower Marker
>60000	1.16	-	99.12	28102	>60000		
-	-	-	-	-	-		Sample Well

 © Agilent Technologies, Inc. 2016

E1: Qubit HS Standard #2

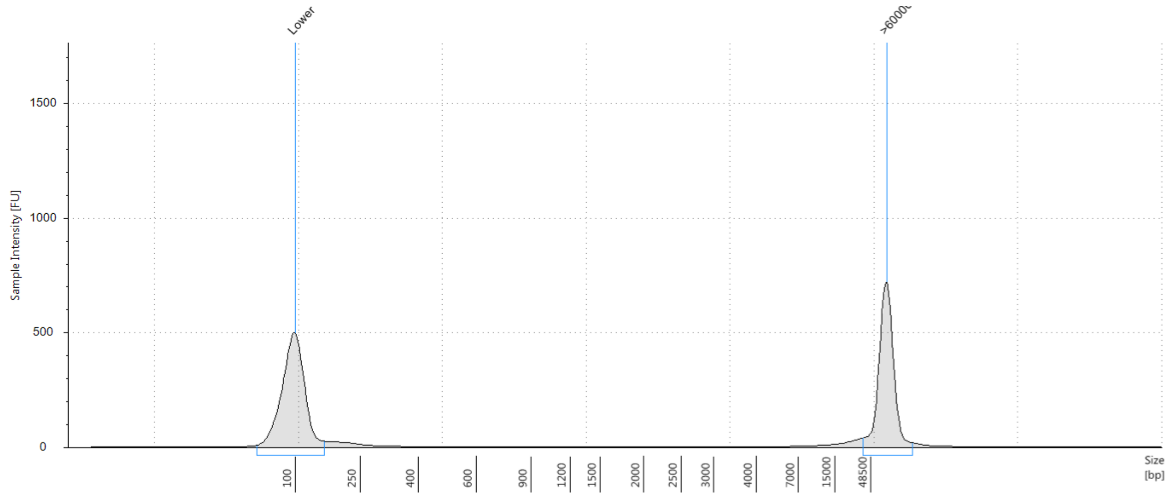

Sample Table

Well	DIN	Conc. [ng/µl]	Sample Description	Alert	Observations
E1	9.9	9.72	Qubit HS Standard #2	⚠	Sample concentration outside recommended range

Peak Table

Size [bp]	Calibrated Conc. [ng/µl]	Assigned Conc. [ng/µl]	% Integrated Area	From [bp]	To [bp]	Peak Comment	Observations
100	8.50	8.50	-	58	151		Lower Marker
>60000	7.89	-	100.00	27632	>60000		

 © Agilent Technologies, Inc. 2016
