# Supplementary material for: Linked-read sequencing of gametes allows efficient genome-wide analysis of meiotic recombination
Source: Nat Commun. 2019 Sep 20;10:4310. doi: 10.1038/s41467-019-12209-2 (PMC6754367; doi:10.1038/s41467-019-12209-2)
Supplement: Supplementary file 12 — Source Data [file 41467_2019_12209_MOESM12_ESM.zip › Source_Data/Source_Data_supplementary_Figure_1.zip!/Source_Data_supplementary_Figure_1/supplementary_Figure_1_3358_ABC_after_BluePippin_sub.pdf]

Filename: 2017-12-07-01-3358_A_B_C_D_after BluePippin.gDNA

Gel Image

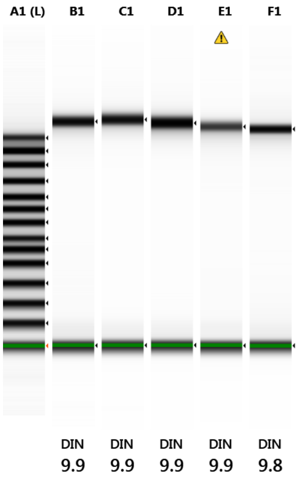

Default image (Contrast 100%)

Sample Info

Well	DIN	Conc. [ng/µl]	Sample Description	Alert	Observations
A1	-	122	Ladder		Ladder
B1	9.9	12.2	3358.A.1 after Blue Pippin purification		
C1	9.9	12.0	3358.B.1 after Blue Pippin purification		
D1	9.9	15.6	3358.C.1 after Blue Pippin purification		
E1	9.9	8.67	3358.D.1 after Blue Pippin purification	⚠	Sample concentration outside recommended range
F1	9.8	11.6	Qubit HS Standard #2		

 © Agilent Technologies, Inc. 2016

A1: Ladder

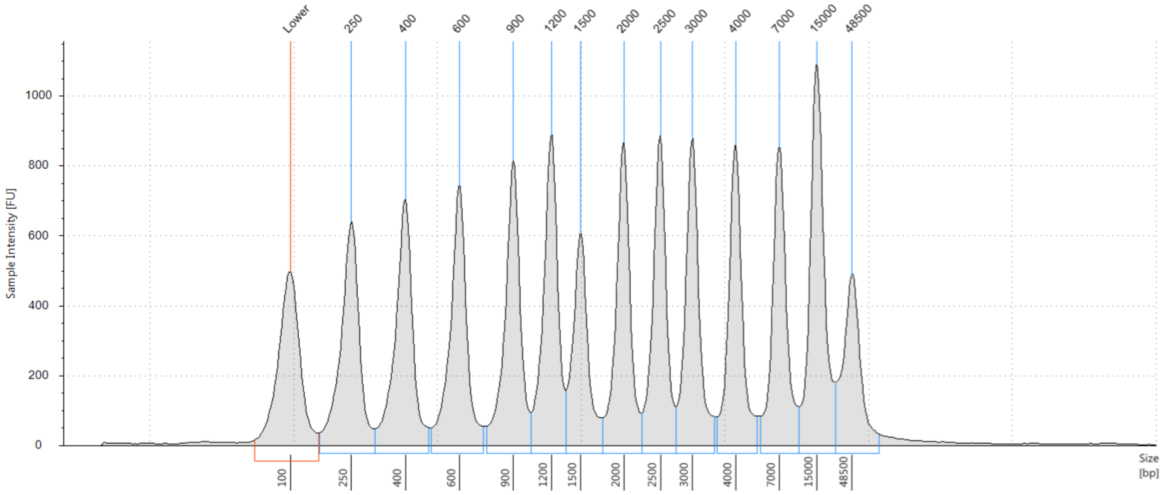

Sample Table

Well	DIN	Conc. [ng/µl]	Sample Description	Alert	Observations
A1	-	122	Ladder		Ladder

Peak Table

Size [bp]	Calibrated Conc. [ng/µl]	Assigned Conc. [ng/µl]	% Integrated Area	From [bp]	To [bp]	Peak Comment	Observations
100	8.50	8.50	-	59	153		Lower Marker
250	9.17	-	7.58	155	307		
400	9.50	-	7.85	307	477		
600	9.47	-	7.83	486	716		
900	9.73	-	8.04	735	1030		
1200	10.1	-	8.33	1030	1348		
1500	7.17	-	5.92	1348	1739		
2000	9.46	-	7.82	1739	2228		
2500	9.36	-	7.74	2228	2729		
3000	9.51	-	7.86	2729	3478		
4000	9.24	-	7.63	3532	5252		
7000	9.37	-	7.74	5495	10494		
15000	12.3	-	10.14	10494	21964		
48500	6.67	-	5.51	21964	>60000		

© Agilent Technologies, Inc. 2016

B1: 3358.A.1 after Blue Pippin purification

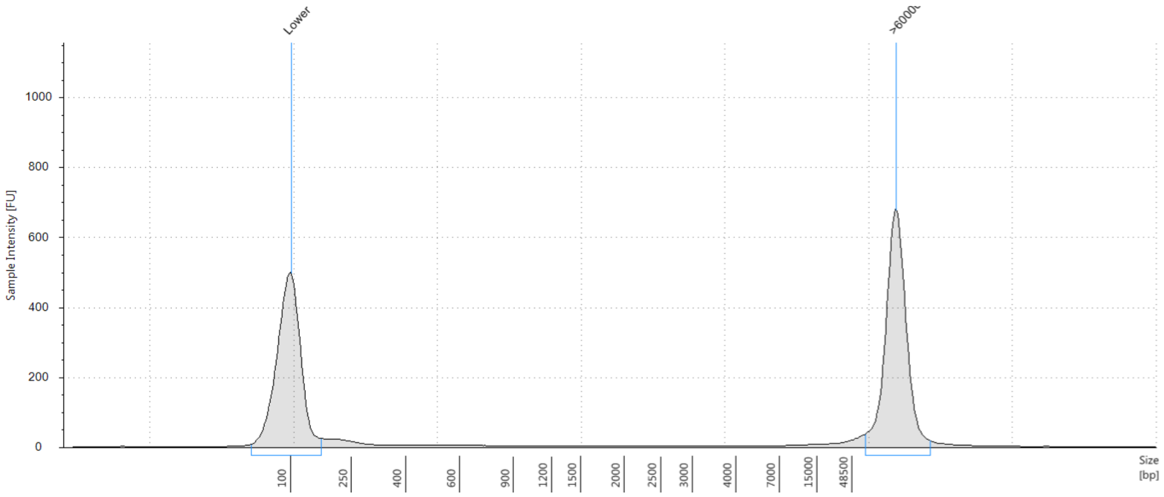

Sample Table

Well	DIN	Conc. [ng/µl]	Sample Description	Alert	Observations
B1	9.9	12.2	3358.A.1 after Blue Pippin purification		

Peak Table

Size [bp]	Calibrated Conc. [ng/µl]	Assigned Conc. [ng/µl]	% Integrated Area	From [bp]	To [bp]	Peak Comment	Observations
100	8.50	8.50	-	55	157		Lower Marker
>60000	9.97	-	100.00	>60000	>60000		

 © Agilent Technologies, Inc. 2016

C1: 3358.B.1 after Blue Pippin purification

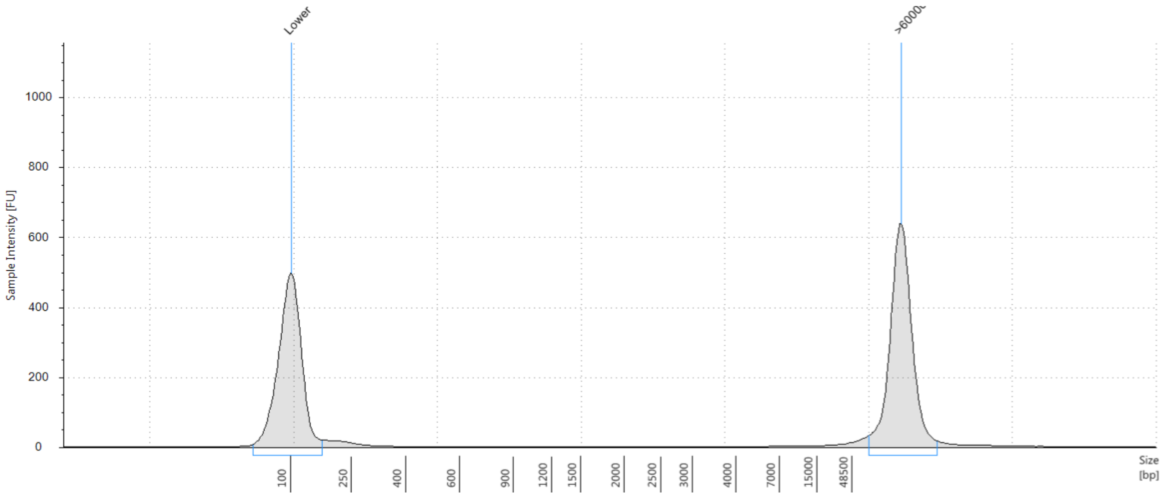

Sample Table

Well	DIN	Conc. [ng/µl]	Sample Description	Alert	Observations
C1	9.9	12.0	3358.B.1 after Blue Pippin purification		

Peak Table

Size [bp]	Calibrated Conc. [ng/µl]	Assigned Conc. [ng/µl]	% Integrated Area	From [bp]	To [bp]	Peak Comment	Observations
100	8.50	8.50	-	56	158		Lower Marker
>60000	10.2	-	100.00	>60000	>60000		

 © Agilent Technologies, Inc. 2016

D1: 3358.C.1 after Blue Pippin purification

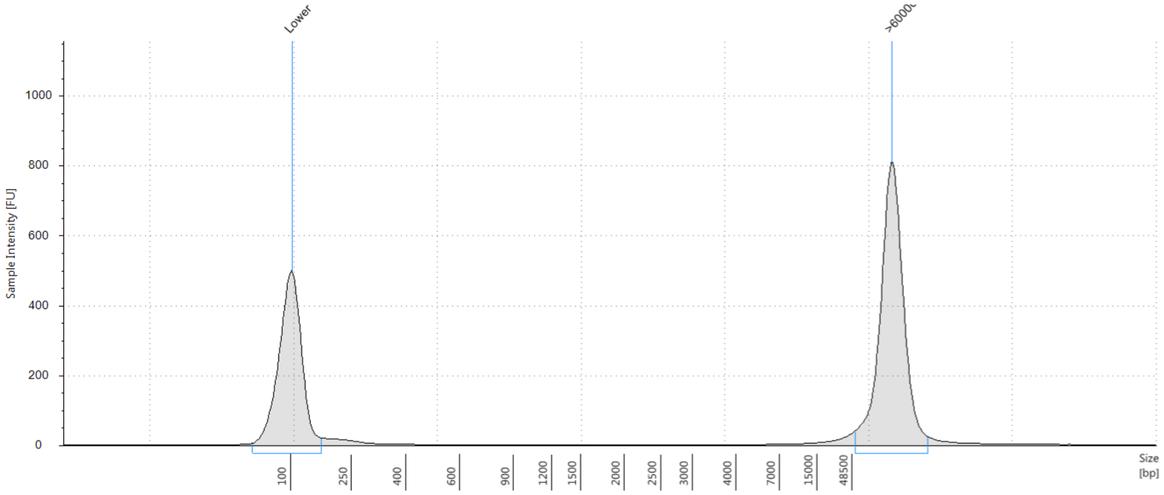

Sample Table

Well	DIN	Conc. [ng/µl]	Sample Description	Alert	Observations
D1	9.9	15.6	3358.C.1 after Blue Pippin purification		

Peak Table

Size [bp]	Calibrated Conc. [ng/µl]	Assigned Conc. [ng/µl]	% Integrated Area	From [bp]	To [bp]	Peak Comment	Observations
100	8.50	8.50	-	55	156		Lower Marker
>60000	14.0	-	100.00	51054	>60000		

 © Agilent Technologies, Inc. 2016

F1: Qubit HS Standard #2

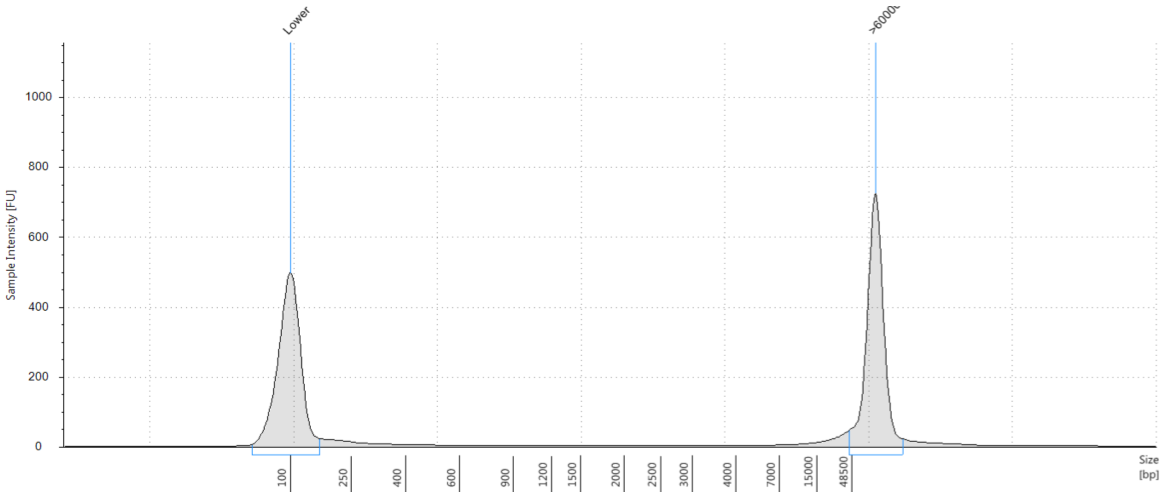

Sample Table

Well	DIN	Conc. [ng/µl]	Sample Description	Alert	Observations
F1	9.8	11.6	Qubit HS Standard #2		

Peak Table

Size [bp]	Calibrated Conc. [ng/µl]	Assigned Conc. [ng/µl]	% Integrated Area	From [bp]	To [bp]	Peak Comment	Observations
100	8.50	8.50	-	56	155		Lower Marker
>60000	9.02	-	100.00	33833	>60000		

 © Agilent Technologies, Inc. 2016
