# Supplementary material for: Linked-read sequencing of gametes allows efficient genome-wide analysis of meiotic recombination
Source: Nat Commun. 2019 Sep 20;10:4310. doi: 10.1038/s41467-019-12209-2 (PMC6754367; doi:10.1038/s41467-019-12209-2)
Supplement: Supplementary file 12 — Source Data [file 41467_2019_12209_MOESM12_ESM.zip › Source_Data/Source_Data_supplementary_Figure_4.zip!/Source_Data_supplementary_Figure_4/supplemenary_Figure_4_3358_FEMTO_D1-D11_Summary.pdf]

FEMTO Pulse Run Summary:

Filename and Data Path: Z:\project_data\LIMS_projects\
Filename and Data Path: 3358_He_Quan_Sun_10X_Genomics_for_recombination_break_identification_MPIPZ\
Filename and Data Path: Lab\15-43-24\2017 12 13 15H 43M.raw
Created: 13-Dec-17 4:00 PM
# of Capillaries: 12
Array Serial #: 041017-03USFP
Effect Length: 22 cm
Array Usage Count: 61
FEMTO Pulse Version #: 1.0.0.32
Device Serial #: F12119

METHOD INFORMATION
Method Name: FP-1002-22 - gDNA 165Kb.mthds
Gel Prime: No
Full Conditioning: Yes
Gel Prime to Buffer: No
Gel Selection: Gel 1
Perform Prerun: -5.0 kV, 60 sec.
Rinse: No
Marker 1: No
Rinse: Tray: 3, Row: A, # Dips: 1
Sample Injection: -5.0 kV, 30 sec.
Separation: 70.0 min.
Tray Name: Tray-1

Analysis Mode: gDNA

NOTES

Time Shift: Using configured: slope=0.00025, intercept=0.0

 Copyright 2015 Advanced Analytical Technologies, Inc.

Gel Image

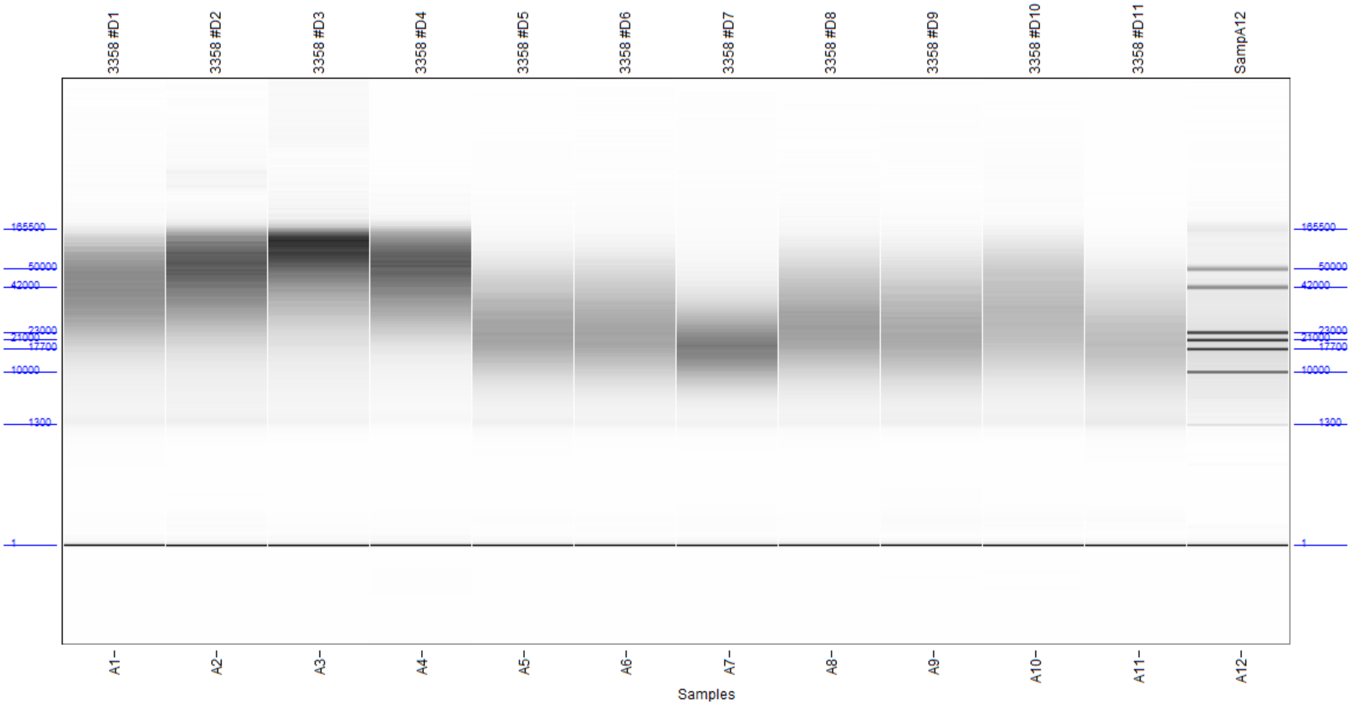

 Copyright 2015 Advanced Analytical Technologies, Inc.

GQN Summary

Well	Sample ID	GQN	Size Threshold (bp)	Total Conc. (ng/uL)
A1	3358 #D1	2.3	50000	0.3602
A2	3358 #D2	3.6	50000	0.4704
A3	3358 #D3	5.1	50000	0.4532
A4	3358 #D4	3.9	50000	0.4296
A5	3358 #D5	0.6	50000	0.3113
A6	3358 #D6	0.7	50000	0.3218
A7	3358 #D7	0.3	50000	0.3356
A8	3358 #D8	0.9	50000	0.3293
A9	3358 #D9	0.7	50000	0.3111
A10	3358 #D10	1.4	50000	0.2881
A11	3358 #D11	0.3	50000	0.2375
A12	SampA12			

 Copyright 2015 Advanced Analytical Technologies, Inc.

Filename and Data Path: Z:\project_data\LIMS_projects\
3358_He_Quan_Sun_10X_Genomics_for_recombination_break_identification_MPIPZ\Lab\15-43-24\
2017 12 13 15H 43M.raw

A1: 3358 #D1

GQN: 2.3

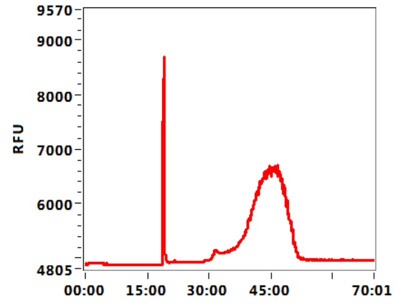

A2: 3358 #D2

GQN: 3.6

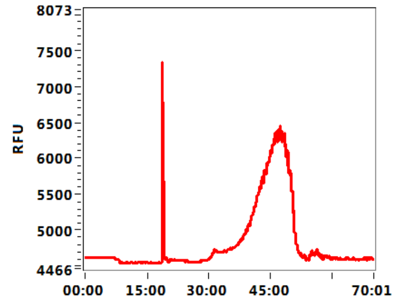

A3: 3358 #D3

GQN: 5.1

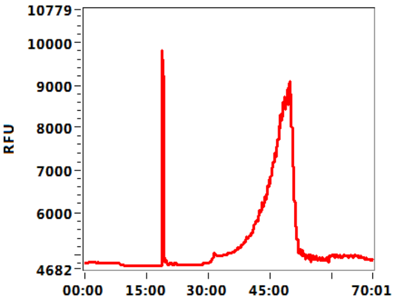

A4: 3358 #D4

GQN: 3.9

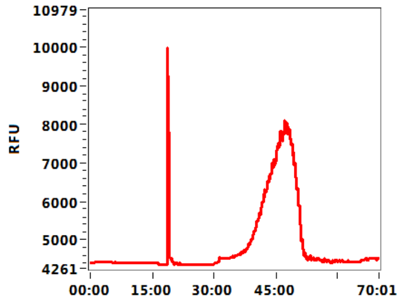

A5: 3358 #D5

GQN: 0.6

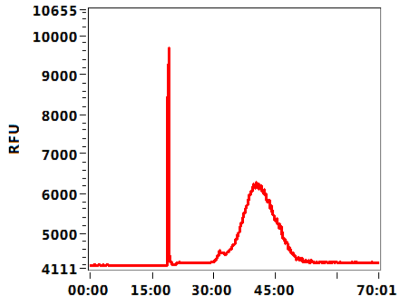

A6: 3358 #D6

GQN: 0.7

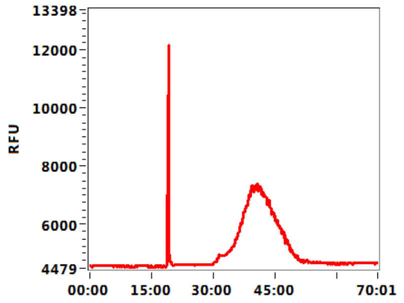

A7: 3358 #D7

GQN: 0.3

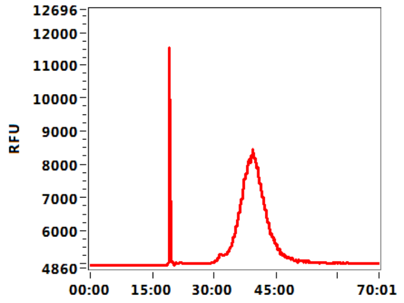

A8: 3358 #D8

GQN: 0.9

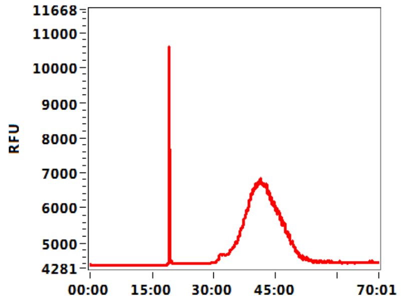

A9: 3358 #D9

GQN: 0.7

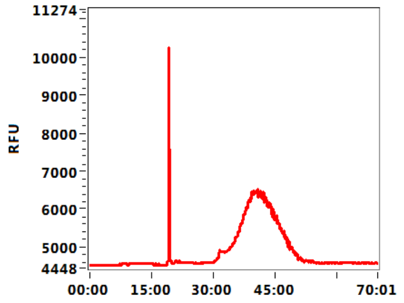

A10: 3358 #D10

GQN: 1.4

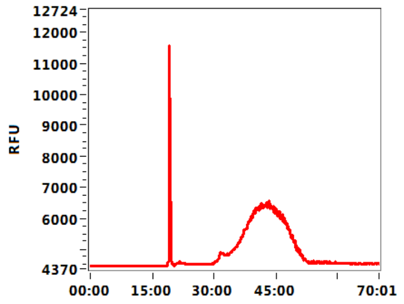

A11: 3358 #D11

GQN: 0.3

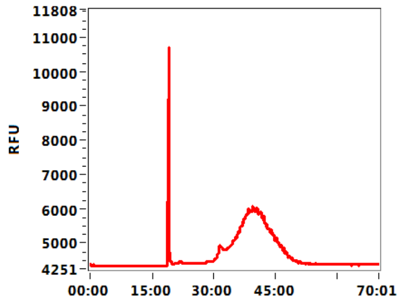

A12: SampA12

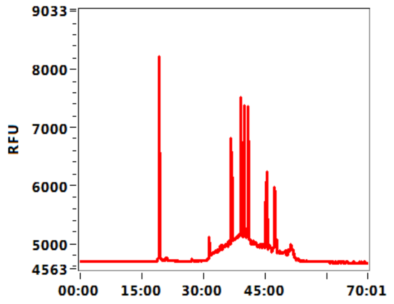

 Copyright 2015 Advanced Analytical Technologies, Inc.

Sample: 3358 #D1
Well Location: A1
Created: 13-Dec-17 4:00 PM

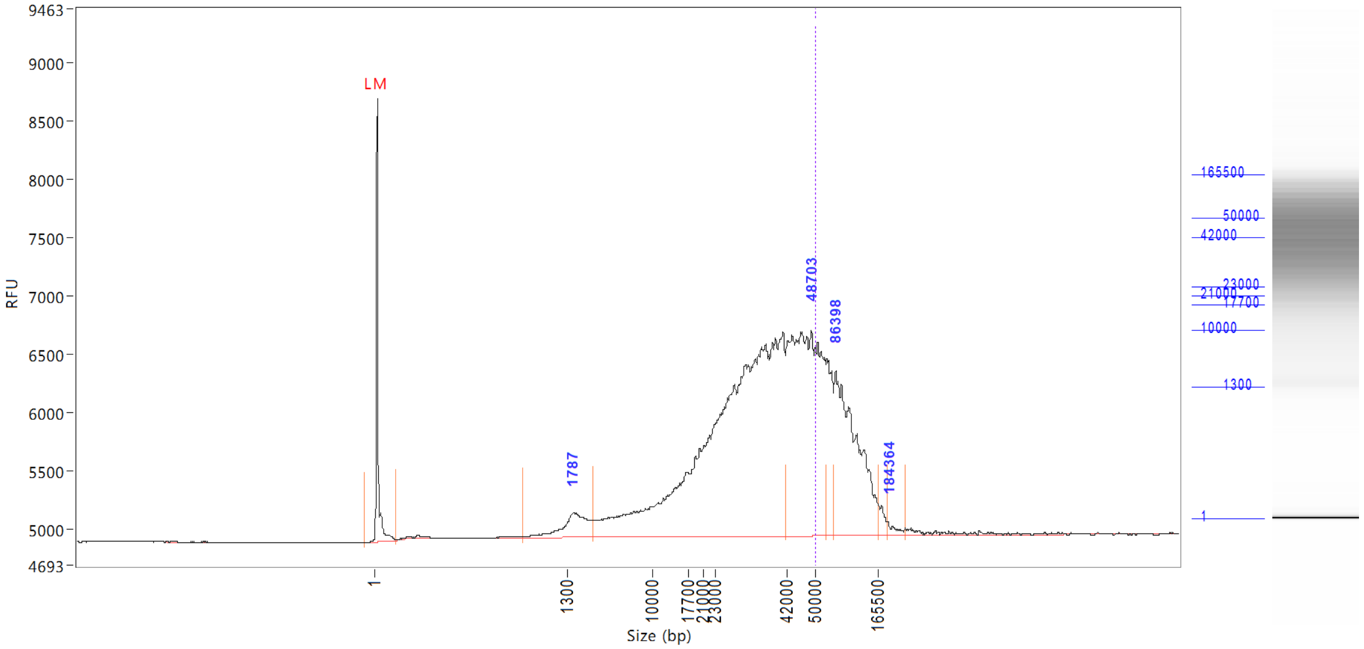

Peak	Size (bp)	Conc. (ng/uL)	From (bp)	To (bp)	Avg. Size (bp)	CV%	RFU	Corr. Peak Area
1	1 (LM)	NaN	0	0	>200000	NaN	0	NaN
2	0	NaN	0	0	>200000	NaN	0	NaN
3	1	0.0023	0	135	8	281.48	3807	25.533
4	1787	0.0115	996	3894	2091	41.25	209	12.621
5	48703	0.0827	42000	69898	49397	14.15	1767	90.874
6	86398	0.0434	83971	165983	115385	18.90	1410	47.688
7	184364	0.0012	183396	>200000	197489	5.04	122	1.280

TIC:	NaN	ng/uL
TIM:	NaN	nmole/L
Total Conc.:	0.3602	ng/uL
GQN:	2.3	
Threshold:	50000	

Sample Peak Width (sec): 20   Sample Min Peak Height: 50   Sample Baseline V to V?: Y   Sample Baseline V to V pts: 3
Sample Filter: Binomial   # of Pts for Filter: 3   Sample Start Region (min): 0   Sample End Region (min): 70
Manual Baseline Start (min): 25   Manual Baseline End (min): 70
Marker Peak Width (sec): 8   Marker Min Peak Height: 500   Marker Baseline V to V?: N   Marker Baseline V to V pts: 3
Lower Marker Selection: First Peak > 500 RFU   Upper Marker Selection: Last Peak > 500 RFU
Ladder Size (bp)1, 1300, 10000, 17700, 21000, 23000, 42000, 50000, 165500
Quantification Using: Ladder   Final Concentration (ng/uL): 0.0200   Dilution Factor: 10.0
Min. RFU for Data Processing: 1   Size Threshold (b.p.): 50000

 Copyright 2015 Advanced Analytical Technologies, Inc.

Sample: 3358 #D2
Well Location: A2
Created: 13-Dec-17 4:00 PM

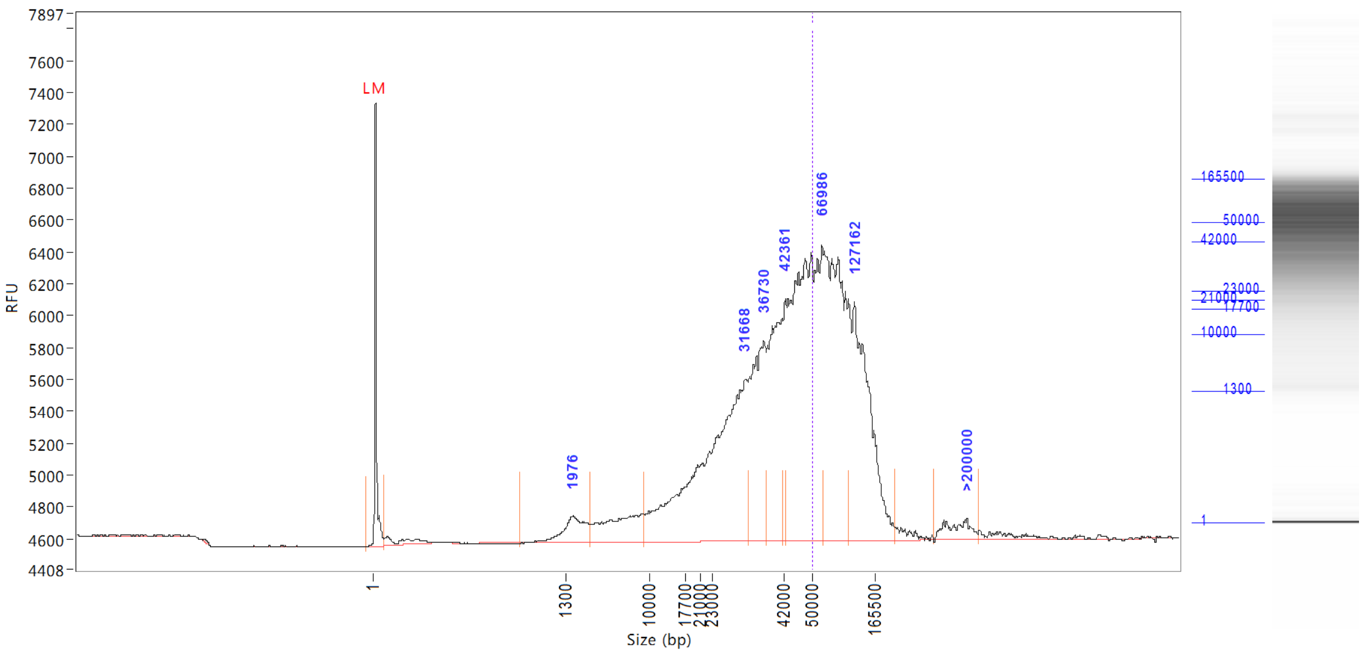

Peak	Size (bp)	Conc. (ng/uL)	From (bp)	To (bp)	Avg. Size (bp)	CV%	RFU	Corr. Peak Area
1	1 (LM)	0.0023	0	57	2	518.83	2786	18.137
2	1976	0.0108	991	3759	2159	37.50	165	8.403
3	31668	0.1033	9460	32500	23298	27.06	1017	80.586
4	36730	0.0391	32500	37354	35008	4.04	1253	30.485
5	42361	0.0509	37354	42794	40165	3.95	1517	39.701
6	66986	0.1170	41931	69898	49953	14.62	1855	91.264
7	127162	0.0564	117456	>200000	144162	13.14	1494	44.002
8	>200000	0.0049	>200000	>200000	>200000	6.67	138	3.785

TIC:	0.3822	ng/uL
TIM:	0.0240	nmole/L
Total Conc.:	0.4704	ng/uL
GQN:	3.6	
Threshold:	50000	

Sample Peak Width (sec): 20 Sample Min Peak Height: 50 Sample Baseline V to V?: Y Sample Baseline V to V pts: 3
Sample Filter: Binomial # of Pts for Filter: 3 Sample Start Region (min): 0 Sample End Region (min): 70
Manual Baseline Start (min): 25 Manual Baseline End (min): 70
Marker Peak Width (sec): 8 Marker Min Peak Height: 500 Marker Baseline V to V?: N Marker Baseline V to V pts: 3
Lower Marker Selection: First Peak > 500 RFU Upper Marker Selection: Last Peak > 500 RFU
Ladder Size (bp)1, 1300, 10000, 17700, 21000, 23000, 42000, 50000, 165500
Quantification Using: Ladder Final Concentration (ng/uL): 0.0200 Dilution Factor: 10.0
Min. RFU for Data Processing: 1 Size Threshold (b.p.): 50000

 Copyright 2015 Advanced Analytical Technologies, Inc.

Sample: 3358 #D3
Well Location: A3
Created: 13-Dec-17 4:00 PM

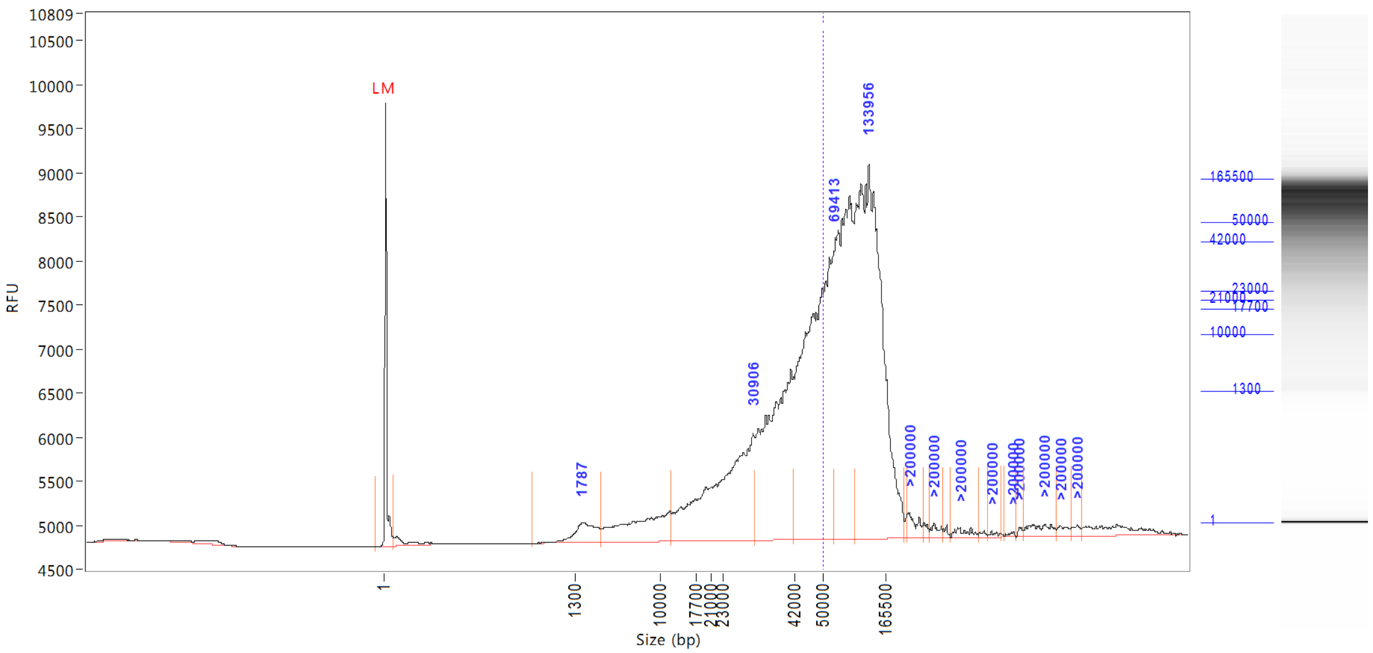

Peak	Size (bp)	Conc. (ng/uL)	From (bp)	To (bp)	Avg. Size (bp)	CV%	RFU	Corr. Peak Area
1	1 (LM)	0.0023	0	55	2	523.55	5041	32.601
2	1787	0.0083	1003	3840	2227	37.06	225	11.689
3	30906	0.0619	12288	31460	23229	22.90	1216	86.847
4	69413	0.1007	41931	69898	50631	14.97	3271	141.281
5	133956	0.1148	106295	>200000	138268	15.14	4249	161.066
6	>200000	0.0029	>200000	>200000	>200000	4.25	295	4.005
7	>200000	0.0012	>200000	>200000	>200000	2.62	162	1.648
8	>200000	0.0014	>200000	>200000	>200000	4.54	109	2.022
9	>200000	0.0004	>200000	>200000	>200000	1.88	71	0.556
10	>200000	0.0003	>200000	>200000	>200000	1.33	62	0.406
11	>200000	0.0004	>200000	>200000	>200000	0.75	114	0.565
12	>200000	0.0027	>200000	>200000	>200000	3.87	141	3.751
13	>200000	0.0010	>200000	>200000	>200000	1.59	120	1.458
14	>200000	0.0007	>200000	>200000	>200000	1.02	126	1.026

TIC:	0.2968	ng/uL
TIM:	0.0152	nmole/L
Total Conc.:	0.4532	ng/uL

GQN:	5.1
Threshold:	50000

```
Sample Peak Width (sec): 20   Sample Min Peak Height: 50    Sample Baseline V to V?: Y    Sample Baseline V to V pts: 3
Sample Filter: Binomial       # of Pts for Filter: 3        Sample Start Region (min): 0  Sample End Region (min): 70
Manual Baseline Start (min): 25     Manual Baseline End (min): 70
Marker Peak Width (sec): 8    Marker Min Peak Height: 500   Marker Baseline V to V?: N    Marker Baseline V to V pts: 3
Lower Marker Selection: First Peak > 500 RFU                Upper Marker Selection: Last Peak > 500 RFU
Ladder Size (bp)1, 1300, 10000, 17700, 21000, 23000, 42000, 50000, 165500
Quantification Using: Ladder          Final Concentration (ng/uL): 0.0200          Dilution Factor: 10.0
Min. RFU for Data Processing: 1      Size Threshold (b.p.): 50000
```


Copyright 2015 Advanced Analytical Technologies, Inc.

Sample: 3358 #D4
Well Location: A4
Created: 13-Dec-17 4:00 PM

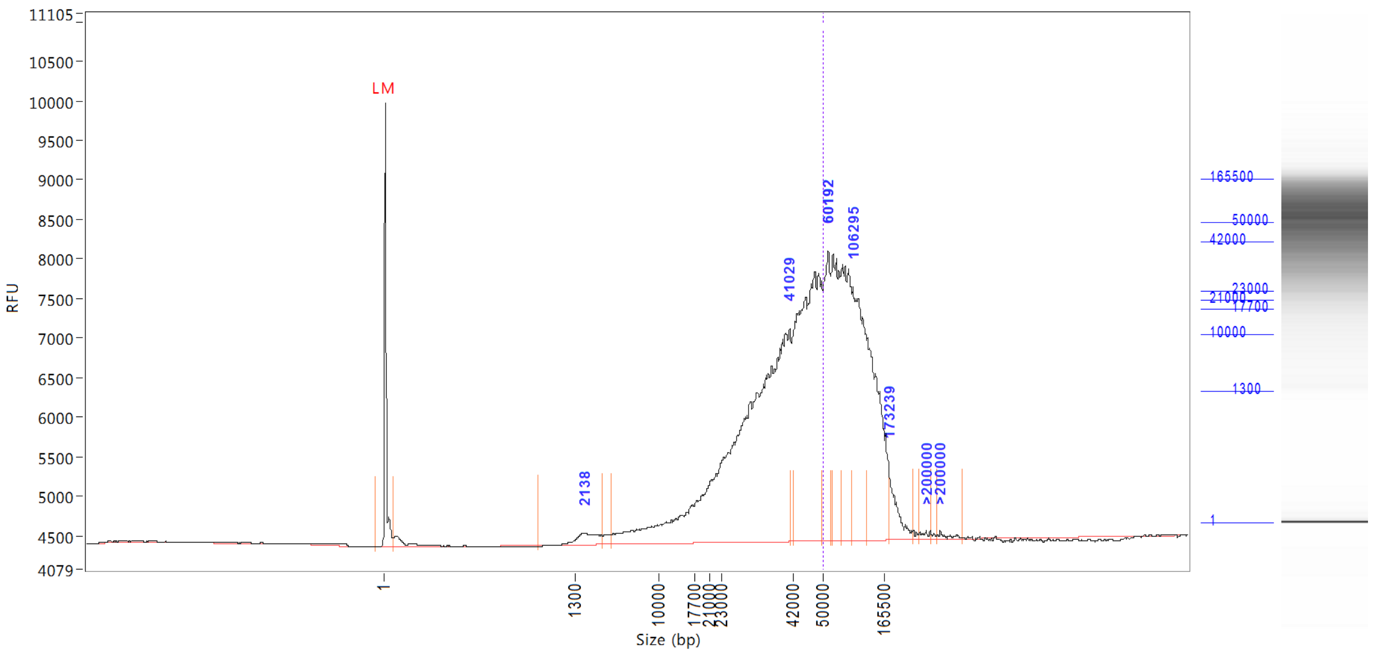

Peak	Size (bp)	Conc. (ng/uL)	From (bp)	To (bp)	Avg. Size (bp)	CV%	RFU	Corr. Peak Area
1	1 (LM)	0.0023	0	55	3	356.24	5618	35.335
2	2138	0.0048	1045	4030	2488	33.77	151	7.264
3	41029	0.1666	5029	41445	28879	32.42	2684	253.219
4	60192	0.1138	41931	68442	49647	13.79	3659	173.006
5	60192	0.0594	50001	86398	68145	15.50	3659	90.298
6	60192	0.1091	42000	66016	49023	12.27	3659	165.821
7	106295	0.0361	105324	132500	118355	6.70	3200	54.828
8	173239	0.0067	173239	>200000	187532	6.30	944	10.190
9	>200000	0.0007	>200000	>200000	>200000	2.70	112	1.033
10	>200000	0.0007	>200000	>200000	>200000	4.76	104	1.064

TIC:	0.4978	ng/uL
TIM:	0.0221	nmole/L
Total Conc.:	0.4296	ng/uL
GQN:	3.9	
Threshold:	50000	

Sample Peak Width (sec): 20   Sample Min Peak Height: 50   Sample Baseline V to V?: Y   Sample Baseline V to V pts: 3
Sample Filter: Binomial   # of Pts for Filter: 3   Sample Start Region (min): 0   Sample End Region (min): 70
Manual Baseline Start (min): 25   Manual Baseline End (min): 70
Marker Peak Width (sec): 8   Marker Min Peak Height: 500   Marker Baseline V to V?: N   Marker Baseline V to V pts: 3
Lower Marker Selection: First Peak > 500 RFU   Upper Marker Selection: Last Peak > 500 RFU
Ladder Size (bp)1, 1300, 10000, 17700, 21000, 23000, 42000, 50000, 165500
Quantification Using: Ladder   Final Concentration (ng/uL): 0.0200   Dilution Factor: 10.0
Min. RFU for Data Processing: 1   Size Threshold (b.p.): 50000

 Copyright 2015 Advanced Analytical Technologies, Inc.

Sample: 3358 #D5
Well Location: A5
Created: 13-Dec-17 4:00 PM

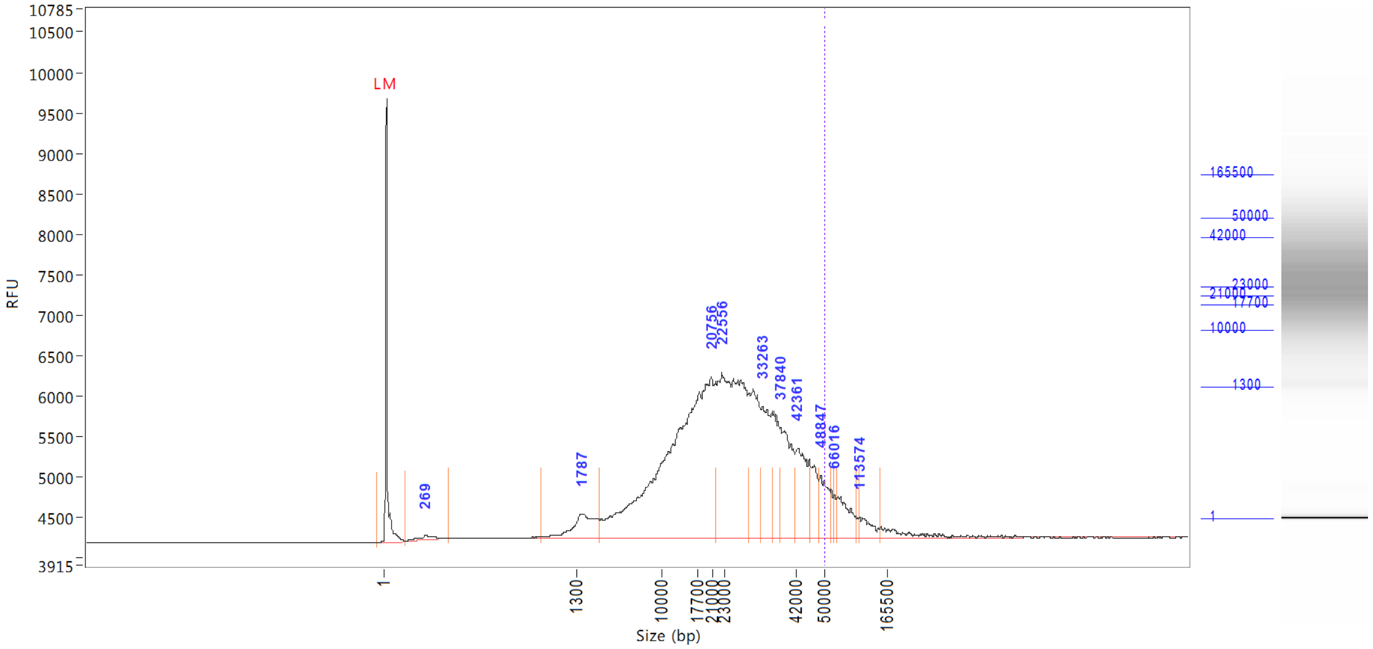

Peak	Size (bp)	Conc. (ng/uL)	From (bp)	To (bp)	Avg. Size (bp)	CV%	RFU	Corr. Peak Area
1	1 (LM)	0.0023	0	129	6	309.15	5490	35.059
2	269	0.0018	129	423	264	21.97	51	2.655
3	1787	0.0109	1054	3597	2023	38.27	304	16.472
4	20756	0.1257	3597	21756	14046	35.94	1996	189.641
5	22556	0.0647	21756	29658	25330	9.36	2046	97.534
6	33263	0.0195	32570	35898	34211	2.87	1626	29.401
7	37840	0.0316	37770	45892	41497	5.66	1363	47.629
8	42361	0.0299	42000	71354	48399	13.83	1103	45.090
9	48847	0.0071	48343	60192	51689	6.90	779	10.781
10	66016	0.0080	65530	108721	84265	14.50	530	12.067
11	113574	0.0031	112603	151912	128813	8.62	259	4.634

TIC:	0.3023	ng/uL
TIM:	0.0424	nmole/L
Total Conc.:	0.3113	ng/uL
GQN:	0.6	
Threshold:	50000	

Sample Peak Width (sec): 20   Sample Min Peak Height: 50   Sample Baseline V to V?: Y   Sample Baseline V to V pts: 3
Sample Filter: Binomial   # of Pts for Filter: 3   Sample Start Region (min): 0   Sample End Region (min): 70
Manual Baseline Start (min): 25   Manual Baseline End (min): 70
Marker Peak Width (sec): 8   Marker Min Peak Height: 500   Marker Baseline V to V?: N   Marker Baseline V to V pts: 3
Lower Marker Selection: First Peak > 500 RFU   Upper Marker Selection: Last Peak > 500 RFU
Ladder Size (bp)1, 1300, 10000, 17700, 21000, 23000, 42000, 50000, 165500
Quantification Using: Ladder   Final Concentration (ng/uL): 0.0200   Dilution Factor: 10.0
Min. RFU for Data Processing: 1   Size Threshold (b.p.): 50000

 Copyright 2015 Advanced Analytical Technologies, Inc.

Sample: 3358 #D6
Well Location: A6
Created: 13-Dec-17 4:00 PM

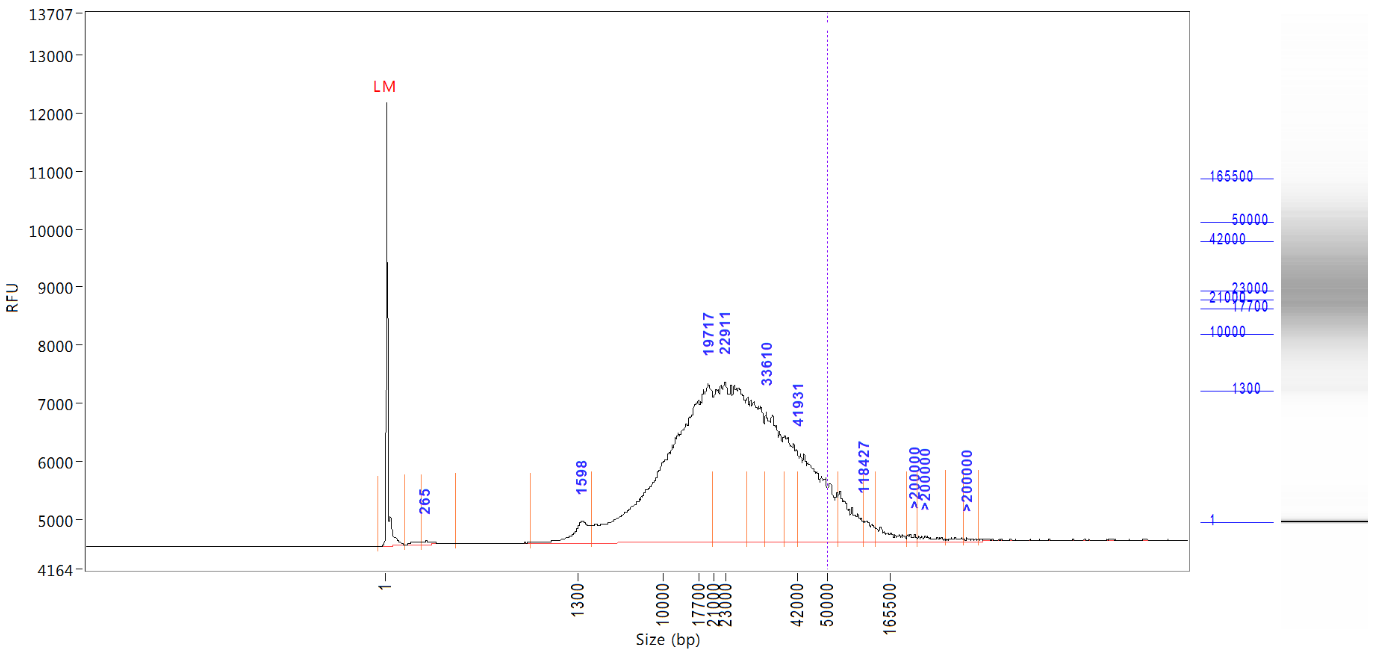

Peak	Size (bp)	Conc. (ng/uL)	From (bp)	To (bp)	Avg. Size (bp)	CV%	RFU	Corr. Peak Area
1	1 (LM)	0.0023	0	133	6	309.95	7622	47.593
2	265	0.0010	239	467	299	15.08	57	2.001
3	1598	0.0072	980	2598	1619	27.81	368	14.721
4	19717	0.1255	2598	20939	13113	37.50	2720	256.969
5	22911	0.0675	20939	28756	24380	9.52	2753	138.273
6	33610	0.0274	33333	38394	35750	4.11	2223	56.080
7	41931	0.0317	41931	70868	48542	13.90	1529	64.953
8	118427	0.0024	117942	141721	128803	5.38	362	4.862
9	>200000	0.0006	197423	>200000	>200000	2.84	118	1.173
10	>200000	0.0009	>200000	>200000	>200000	6.01	99	1.817
11	>200000	0.0003	>200000	>200000	>200000	2.48	71	0.550

TIC:	0.2644	ng/uL
TIM:	0.0354	nmole/L
Total Conc.:	0.3218	ng/uL
GQN:	0.7	
Threshold:	50000	

Sample Peak Width (sec): 20 Sample Min Peak Height: 50 Sample Baseline V to V?: Y Sample Baseline V to V pts: 3
Sample Filter: Binomial # of Pts for Filter: 3 Sample Start Region (min): 0 Sample End Region (min): 70
Manual Baseline Start (min): 25 Manual Baseline End (min): 70
Marker Peak Width (sec): 8 Marker Min Peak Height: 500 Marker Baseline V to V?: N Marker Baseline V to V pts: 3
Lower Marker Selection: First Peak > 500 RFU Upper Marker Selection: Last Peak > 500 RFU
Ladder Size (bp)1, 1300, 10000, 17700, 21000, 23000, 42000, 50000, 165500
Quantification Using: Ladder Final Concentration (ng/uL): 0.0200 Dilution Factor: 10.0
Min. RFU for Data Processing: 1 Size Threshold (b.p.): 50000

 Copyright 2015 Advanced Analytical Technologies, Inc.

Sample: 3358 #D7
Well Location: A7
Created: 13-Dec-17 4:00 PM

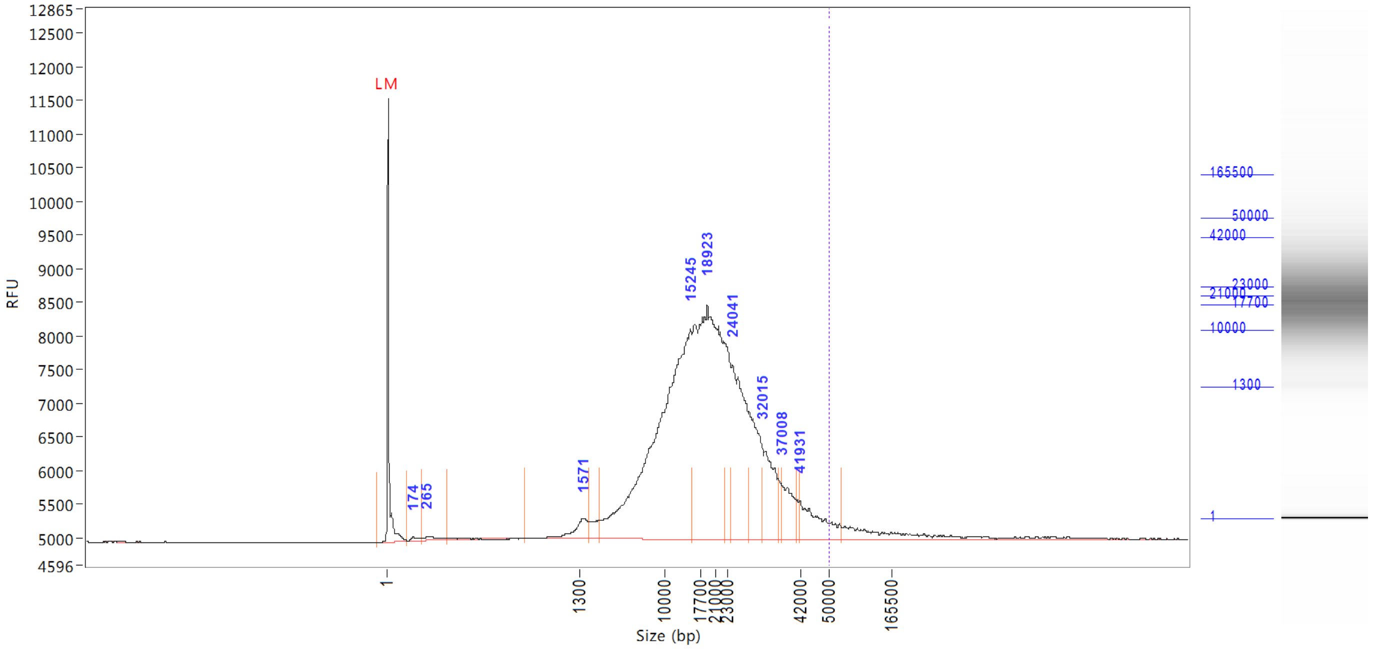

Peak	Size (bp)	Conc. (ng/uL)	From (bp)	To (bp)	Avg. Size (bp)	CV%	RFU	Corr. Peak Area
1	1 (LM)	0.0023	0	135	7	316.17	6605	42.261
2	174	0.0009	135	234	186	13.71	56	1.708
3	265	0.0014	234	398	298	14.14	64	2.480
4	1571	0.0059	925	2165	1443	21.37	302	10.667
5	15245	0.1212	3246	15636	10508	29.92	3118	220.305
6	18923	0.0931	15636	22467	19117	10.61	3486	169.218
7	24041	0.0342	23694	28409	25909	5.30	2589	62.184
8	32015	0.0145	32015	36175	33946	3.56	1367	26.296
9	37008	0.0086	36938	41029	38852	3.06	841	15.642
10	41931	0.0097	41931	70868	48016	13.64	577	17.702

TIC:	0.2894	ng/uL
TIM:	0.0531	nmole/L
Total Conc.:	0.3356	ng/uL
GQN:	0.3	
Threshold:	50000	

Sample Peak Width (sec): 20 Sample Min Peak Height: 50 Sample Baseline V to V?: Y Sample Baseline V to V pts: 3
Sample Filter: Binomial # of Pts for Filter: 3 Sample Start Region (min): 0 Sample End Region (min): 70
Manual Baseline Start (min): 25 Manual Baseline End (min): 70
Marker Peak Width (sec): 8 Marker Min Peak Height: 500 Marker Baseline V to V?: N Marker Baseline V to V pts: 3
Lower Marker Selection: First Peak > 500 RFU Upper Marker Selection: Last Peak > 500 RFU
Ladder Size (bp)1, 1300, 10000, 17700, 21000, 23000, 42000, 50000, 165500
Quantification Using: Ladder Final Concentration (ng/uL): 0.0200 Dilution Factor: 10.0
Min. RFU for Data Processing: 1 Size Threshold (b.p.): 50000

 Copyright 2015 Advanced Analytical Technologies, Inc.

Sample: 3358 #D8
Well Location: A8
Created: 13-Dec-17 4:00 PM

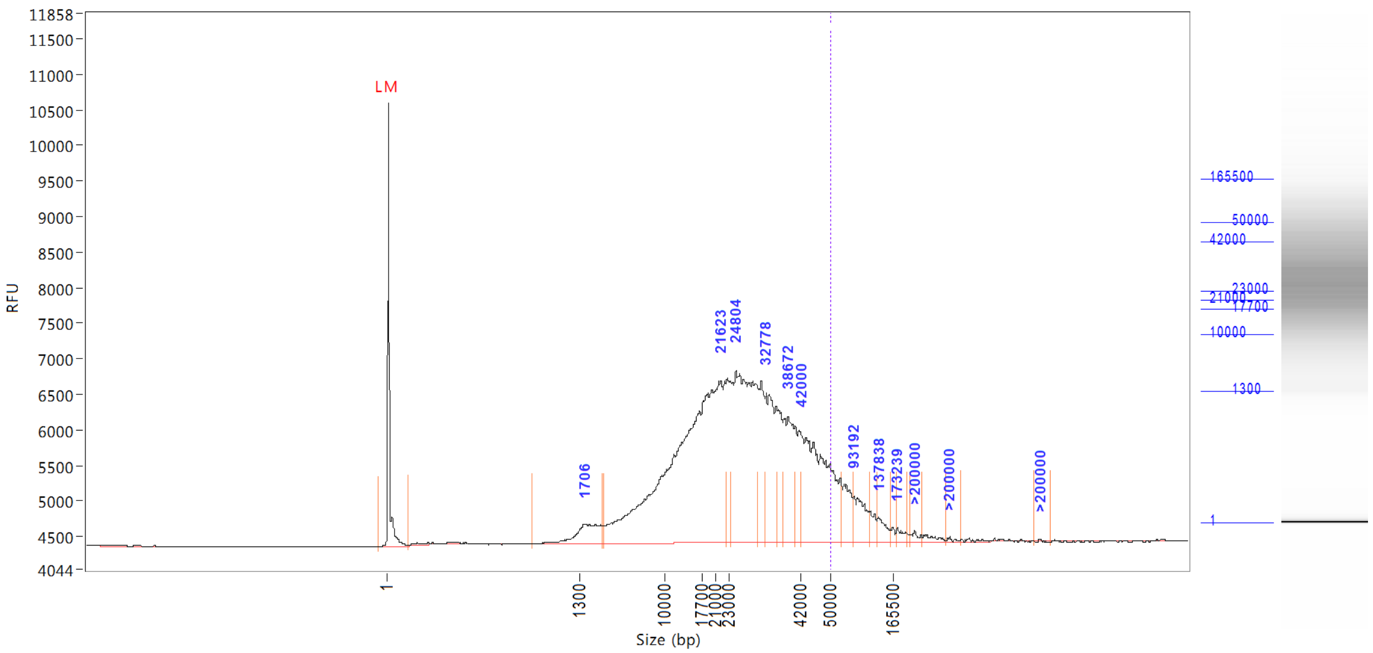

Peak	Size (bp)	Conc. (ng/uL)	From (bp)	To (bp)	Avg. Size (bp)	CV%	RFU	Corr. Peak Area
1	1 (LM)	0.0023	0	140	7	289.03	6244	40.455
2	1706	0.0088	978	3489	2031	36.97	270	15.321
3	21623	0.1320	3678	22645	14729	36.19	2276	229.681
4	24804	0.0518	23694	30559	27073	7.38	2413	90.145
5	32778	0.0211	32500	35829	34123	2.86	2095	36.784
6	38672	0.0166	37354	40475	38888	2.36	1777	28.963
7	42000	0.0377	42000	69898	48546	13.51	1512	65.581
8	93192	0.0068	91736	123280	106268	8.58	651	11.799
9	137838	0.0024	136383	160162	146713	4.67	346	4.171
10	173239	0.0010	171788	190652	180506	3.19	201	1.782
11	>200000	0.0008	196456	>200000	>200000	2.97	156	1.320
12	>200000	0.0002	>200000	>200000	>200000	2.76	66	0.419
13	>200000	0.0000	>200000	>200000	>200000	0.42	36	0.084
14	0	NaN	0	0	>200000	NaN	0	NaN

TIC:	NaN	ng/uL
TIM:	NaN	nmole/L
Total Conc.:	0.3293	ng/uL

GQN:	0.9
Threshold:	50000

```
Sample Peak Width (sec): 20   Sample Min Peak Height: 50    Sample Baseline V to V?: Y    Sample Baseline V to V pts: 3
Sample Filter: Binomial       # of Pts for Filter: 3        Sample Start Region (min): 0  Sample End Region (min): 70
Manual Baseline Start (min): 25     Manual Baseline End (min): 70
Marker Peak Width (sec): 8    Marker Min Peak Height: 500   Marker Baseline V to V?: N    Marker Baseline V to V pts: 3
Lower Marker Selection: First Peak > 500 RFU                Upper Marker Selection: Last Peak > 500 RFU
Ladder Size (bp)1, 1300, 10000, 17700, 21000, 23000, 42000, 50000, 165500
Quantification Using: Ladder          Final Concentration (ng/uL): 0.0200          Dilution Factor: 10.0
Min. RFU for Data Processing: 1      Size Threshold (b.p.): 50000
```

 Copyright 2015 Advanced Analytical Technologies, Inc.

Sample: 3358 #D9
Well Location: A9
Created: 13-Dec-17 4:00 PM

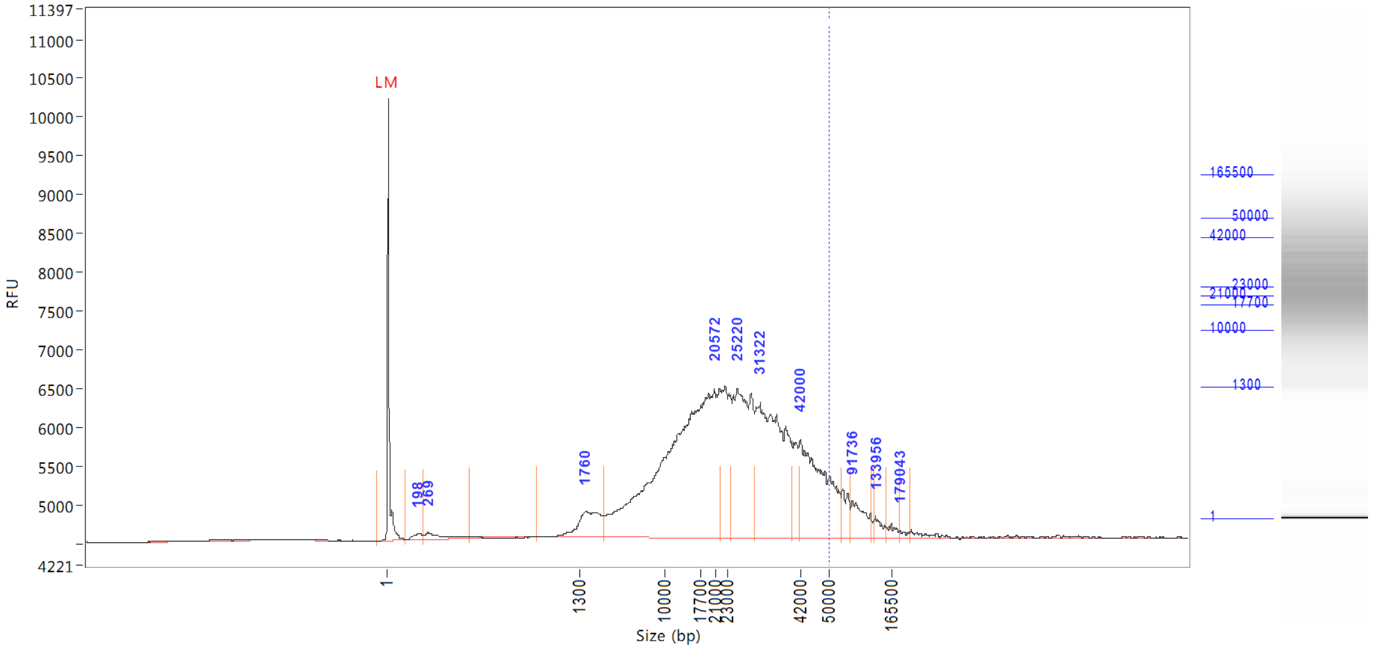

Peak	Size (bp)	Conc. (ng/uL)	From (bp)	To (bp)	Avg. Size (bp)	CV%	RFU	Corr. Peak Area
1	1 (LM)	0.0023	0	126	5	296.98	5711	36.658
2	198	0.0017	126	246	197	14.28	76	2.717
3	269	0.0029	246	557	351	23.68	84	4.548
4	1760	0.0118	1008	3732	2206	36.60	333	18.546
5	20572	0.1279	3732	21712	13648	37.00	1927	201.641
6	25220	0.0398	23833	29866	26792	6.54	1926	62.730
7	31322	0.0532	29866	40058	34638	8.46	1745	83.896
8	42000	0.0308	41931	70868	48478	13.85	1270	48.607
9	91736	0.0060	89309	127647	106208	10.36	474	9.492
10	133956	0.0019	131044	154824	141401	4.90	274	3.018
11	179043	0.0006	178076	198874	187993	3.44	99	0.883

TIC:	0.2765	ng/uL
TIM:	0.0582	nmole/L
Total Conc.:	0.3111	ng/uL
GQN:	0.7	
Threshold:	50000	

Sample Peak Width (sec): 20 Sample Min Peak Height: 50 Sample Baseline V to V?: Y Sample Baseline V to V pts: 3
Sample Filter: Binomial # of Pts for Filter: 3 Sample Start Region (min): 0 Sample End Region (min): 70
Manual Baseline Start (min): 25 Manual Baseline End (min): 70
Marker Peak Width (sec): 8 Marker Min Peak Height: 500 Marker Baseline V to V?: N Marker Baseline V to V pts: 3
Lower Marker Selection: First Peak > 500 RFU Upper Marker Selection: Last Peak > 500 RFU
Ladder Size (bp)1, 1300, 10000, 17700, 21000, 23000, 42000, 50000, 165500
Quantification Using: Ladder Final Concentration (ng/uL): 0.0200 Dilution Factor: 10.0
Min. RFU for Data Processing: 1 Size Threshold (b.p.): 50000

 Copyright 2015 Advanced Analytical Technologies, Inc.

Sample: 3358 #D10
Well Location: A10
Created: 13-Dec-17 4:00 PM

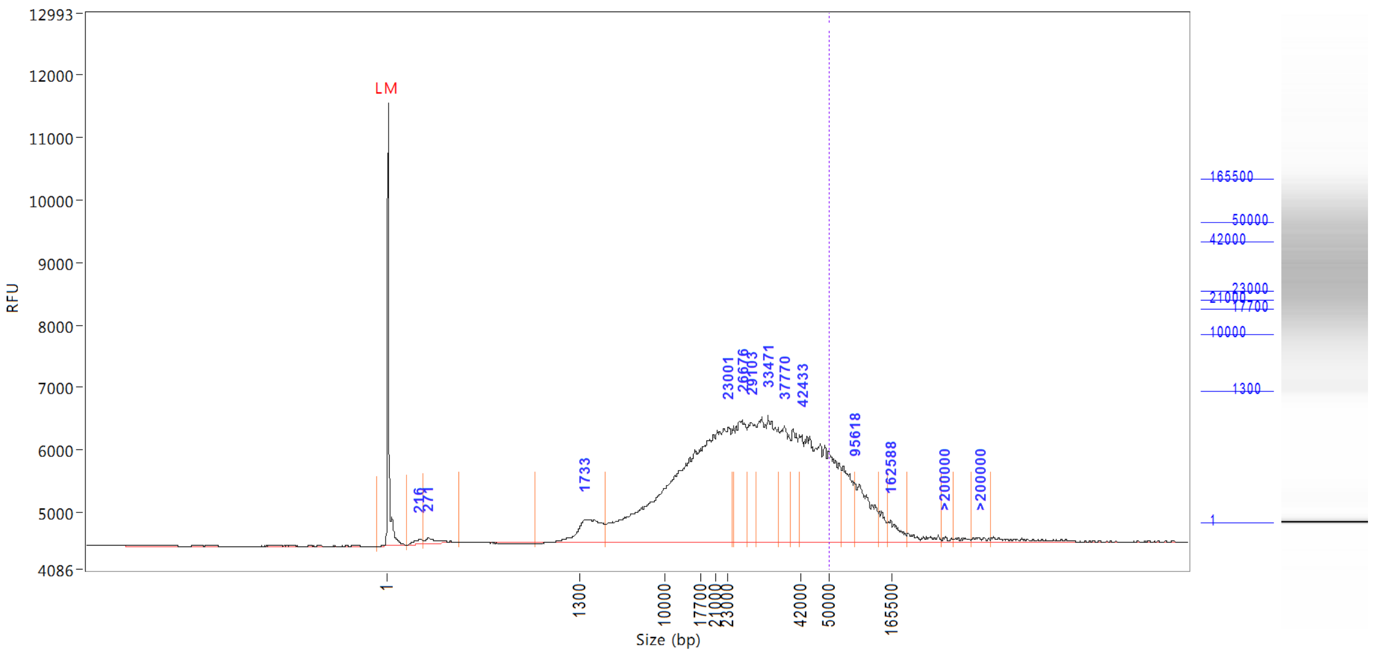

Peak	Size (bp)	Conc. (ng/uL)	From (bp)	To (bp)	Avg. Size (bp)	CV%	RFU	Corr. Peak Area
1	1 (LM)	0.0023	0	128	4	363.43	7108	45.183
2	216	0.0013	128	243	197	13.82	70	2.465
3	271	0.0018	243	483	311	16.46	92	3.587
4	1733	0.0109	996	3894	2258	37.53	369	21.166
5	23001	0.1085	3894	24110	15142	38.05	1837	210.969
6	26676	0.0185	24596	27855	26228	3.62	1967	36.013
7	29103	0.0141	27855	30351	29100	2.53	1914	27.332
8	33471	0.0334	30351	36522	33381	5.36	2024	64.927
9	37770	0.0145	36522	39434	37950	2.24	1854	28.225
10	42433	0.0423	41931	70383	49063	14.16	1718	82.313
11	95618	0.0112	95618	139780	115062	10.96	948	21.844
12	162588	0.0026	158221	192103	171934	5.50	358	4.967
13	>200000	0.0003	>200000	>200000	>200000	2.38	80	0.635
14	>200000	0.0006	>200000	>200000	>200000	3.15	75	1.081

TIC:	0.2601	ng/uL
TIM:	0.0459	nmole/L
Total Conc.:	0.2881	ng/uL
GQN:	1.4	
Threshold:	50000	

```
Sample Peak Width (sec): 20   Sample Min Peak Height: 50    Sample Baseline V to V?: Y    Sample Baseline V to V pts: 3
Sample Filter: Binomial       # of Pts for Filter: 3        Sample Start Region (min): 0  Sample End Region (min): 70
Manual Baseline Start (min): 25     Manual Baseline End (min): 70
Marker Peak Width (sec): 8    Marker Min Peak Height: 500   Marker Baseline V to V?: N    Marker Baseline V to V pts: 3
Lower Marker Selection: First Peak > 500 RFU                Upper Marker Selection: Last Peak > 500 RFU
Ladder Size (bp)1, 1300, 10000, 17700, 21000, 23000, 42000, 50000, 165500
Quantification Using: Ladder          Final Concentration (ng/uL): 0.0200          Dilution Factor: 10.0
Min. RFU for Data Processing: 1      Size Threshold (b.p.): 50000
```


Copyright 2015 Advanced Analytical Technologies, Inc.

Sample: 3358 #D11
Well Location: A11
Created: 13-Dec-17 4:00 PM

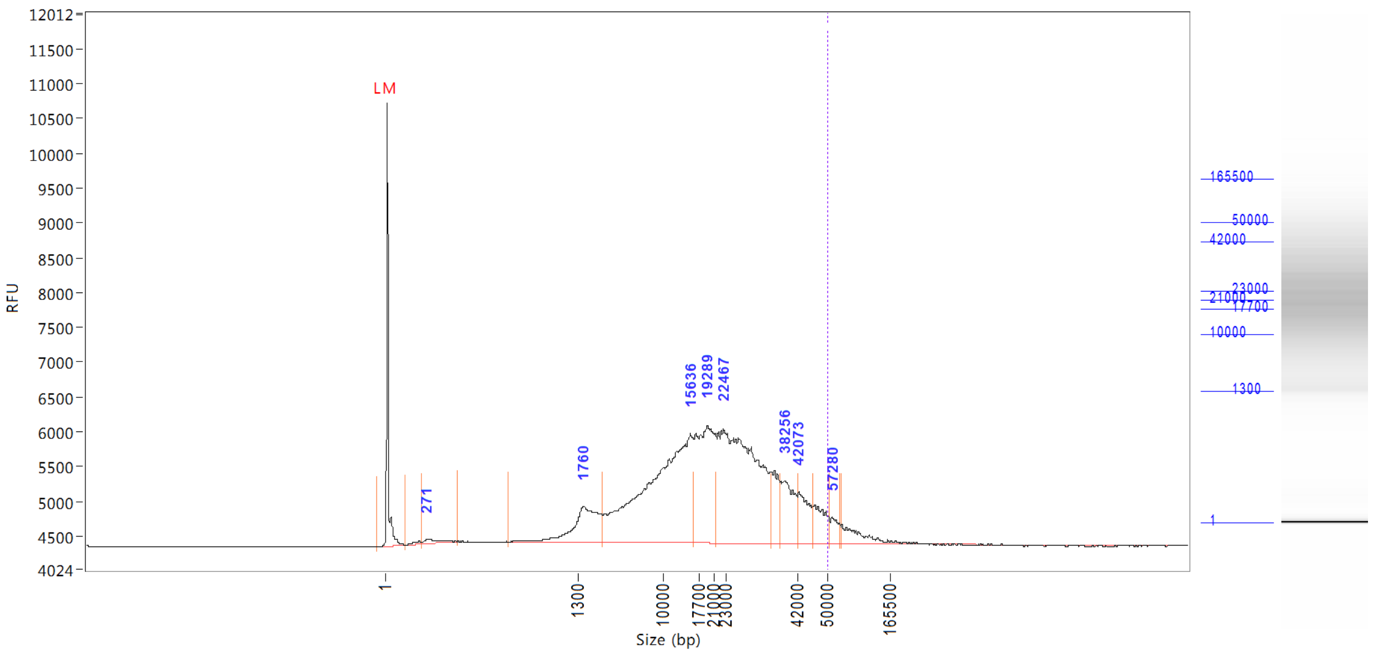

Peak	Size (bp)	Conc. (ng/uL)	From (bp)	To (bp)	Avg. Size (bp)	CV%	RFU	Corr. Peak Area
1	1 (LM)	0.0023	0	128	5	364.15	6381	40.516
2	271	0.0015	237	478	311	16.54	65	2.661
3	1760	0.0179	821	3732	2043	40.73	508	31.173
4	15636	0.0793	3732	16249	10162	34.07	1568	138.144
5	19289	0.0344	16249	21312	18806	8.02	1685	59.953
6	22467	0.0642	21312	34650	26944	14.50	1638	111.943
7	38256	0.0188	37216	45820	41064	5.99	906	32.780
8	42073	0.0161	41931	72810	48529	14.58	741	28.031
9	57280	0.0032	51457	75721	62911	11.12	377	5.558

TIC:	0.2354	ng/uL
TIM:	0.0436	nmole/L
Total Conc.:	0.2375	ng/uL
GQN:	0.3	
Threshold:	50000	

```
Sample Peak Width (sec): 20   Sample Min Peak Height: 50    Sample Baseline V to V?: Y    Sample Baseline V to V pts: 3
Sample Filter: Binomial       # of Pts for Filter: 3        Sample Start Region (min): 0  Sample End Region (min): 70
Manual Baseline Start (min): 25     Manual Baseline End (min): 70
Marker Peak Width (sec): 8    Marker Min Peak Height: 500   Marker Baseline V to V?: N    Marker Baseline V to V pts: 3
Lower Marker Selection: First Peak > 500 RFU                Upper Marker Selection: Last Peak > 500 RFU
Ladder Size (bp)1, 1300, 10000, 17700, 21000, 23000, 42000, 50000, 165500
Quantification Using: Ladder          Final Concentration (ng/uL): 0.0200          Dilution Factor: 10.0
Min. RFU for Data Processing: 1      Size Threshold (b.p.): 50000
```

 Copyright 2015 Advanced Analytical Technologies, Inc.

Sample: SampA12
Well Location: A12
Created: 13-Dec-17 4:00 PM

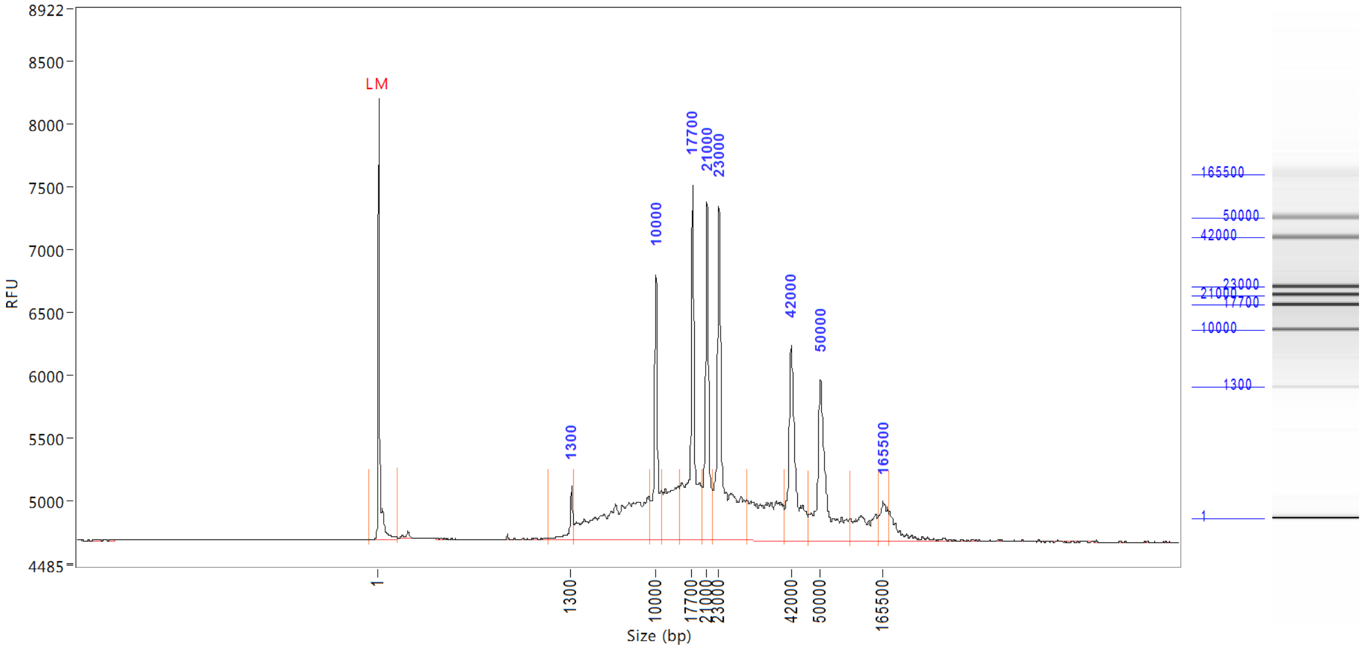

Peak	Size (bp)	Conc. (ng/uL)	From (bp)	To (bp)	Avg. Size (bp)	CV%	RFU	Corr. Peak Area
1	1 (LM)	0.0023	0	129	6	317.60	3525	22.808
2	1300	0.0033	1143	1544	1304	6.03	426	3.262
3	10000	0.0157	9433	11340	10106	4.29	2118	15.357
4	17700	0.0273	14911	19900	17458	6.58	2835	26.803
5	21000	0.0182	19900	21934	20945	2.00	2704	17.817
6	23000	0.0315	21934	30559	24681	9.88	2668	30.927
7	42000	0.0178	40267	46541	42651	3.35	1553	17.469
8	50000	0.0192	46541	105809	60497	26.76	1284	18.853
9	165500	0.0034	157250	176625	166962	3.26	319	3.365

TIC:	0.1364	ng/uL
TIM:	0.0141	nmole/L
Total Conc.:	0.2000	ng/uL

Sample Peak Width (sec): 20   Sample Min Peak Height: 300   Sample Baseline V to V?: Y   Sample Baseline V to V pts: 3
Sample Filter: Binomial   # of Pts for Filter: 3   Sample Start Region (min): 0   Sample End Region (min): 70
Manual Baseline Start (min): 25   Manual Baseline End (min): 70
Marker Peak Width (sec): 8   Marker Min Peak Height: 500   Marker Baseline V to V?: N   Marker Baseline V to V pts: 3
Lower Marker Selection: First Peak > 500 RFU   Upper Marker Selection: Last Peak > 500 RFU
Ladder Size (bp)1, 1300, 10000, 17700, 21000, 23000, 42000, 50000, 165500
Quantification Using: Ladder   Final Concentration (ng/uL): 0.0200   Dilution Factor: 10.0
Min. RFU for Data Processing: 1   Size Threshold (b.p.): 50000

Copyright 2015 Advanced Analytical Technologies, Inc.

Sample: SampA12
Well Location: A12
Created: 13-Dec-17 4:00 PM
Fit Type: Point to Point

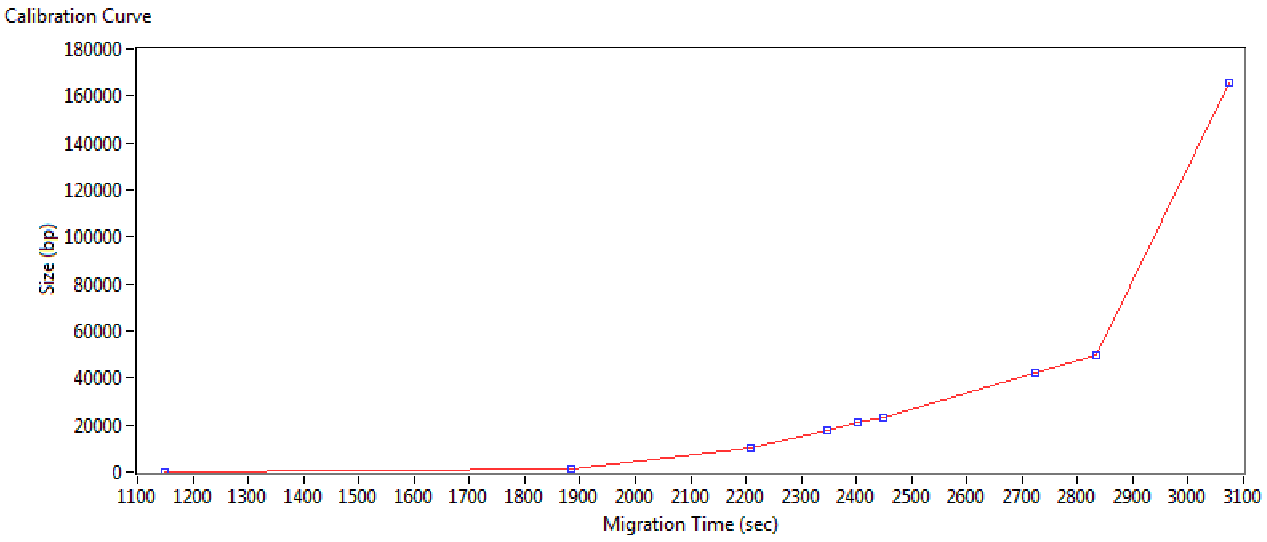

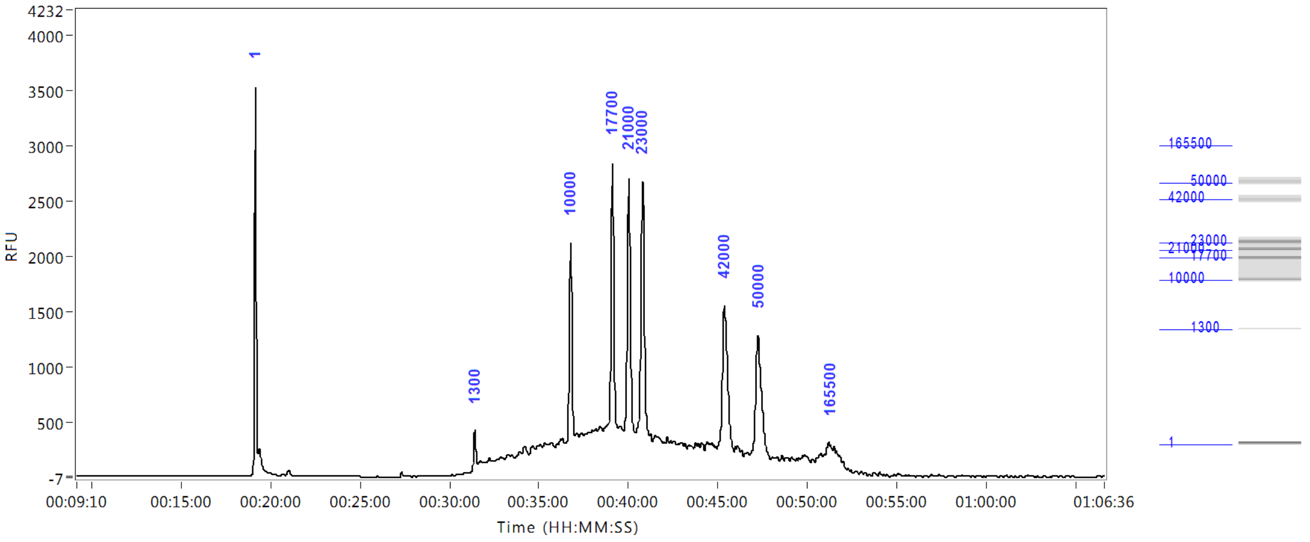

Copyright 2015 Advanced Analytical Technologies, Inc.
