# Supplementary material for: Linked-read sequencing of gametes allows efficient genome-wide analysis of meiotic recombination
Source: Nat Commun. 2019 Sep 20;10:4310. doi: 10.1038/s41467-019-12209-2 (PMC6754367; doi:10.1038/s41467-019-12209-2)

FEMTO Pulse Run Summary:

Filename and Data Path: Z:\project_data\LIMS_projects\
3358_He_Quan_Sun_10X_Genomics_for_recombination_break_identification_MPIPZ\
Public\17-10-46\2017 12 13 17H 10M.raw
Created: 13-Dec-17 5:27 PM
# of Capillaries: 12
Array Serial #: 041017-03USFP
Effect Length: 22 cm
Array Usage Count: 62
FEMTO Pulse Version #: 1.0.0.32
Device Serial #: F12119

METHOD INFORMATION
Method Name: FP-1002-22 - gDNA 165Kb.mthds
Gel Prime: No
Full Conditioning: Yes
Gel Prime to Buffer: No
Gel Selection: Gel 1
Perform Prerun: -5.0 kV, 60 sec.
Rinse: No
Marker 1: No
Rinse: Tray: 3, Row: A, # Dips: 1
Sample Injection: -5.0 kV, 30 sec.
Separation: 70.0 min.
Tray Name: Tray-1

Analysis Mode: gDNA

NOTES

Time Shift: Using configured: slope=0.00025, intercept=0.0

 Copyright 2015 Advanced Analytical Technologies, Inc.

Gel Image

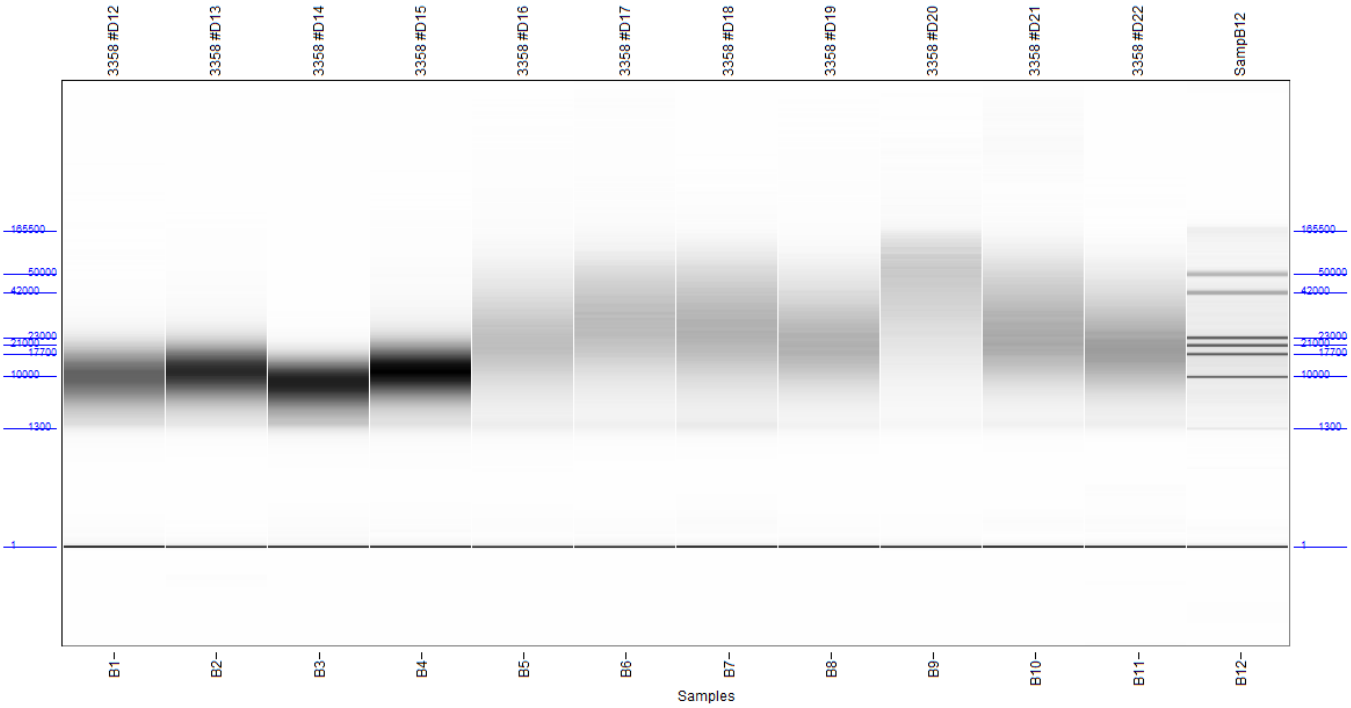

Copyright 2015 Advanced Analytical Technologies, Inc.

GQN Summary

Well	Sample ID	GQN	Size Threshold (bp)	Total Conc. (ng/uL)
B1	3358 #D12	0.0	50000	0.4380
B2	3358 #D13	0.0	50000	0.5403
B3	3358 #D14	0.0	50000	0.5605
B4	3358 #D15	0.0	50000	0.6443
B5	3358 #D16	0.6	50000	0.2766
B6	3358 #D17	1.1	50000	0.3218
B7	3358 #D18	0.9	50000	0.3624
B8	3358 #D19	0.5	50000	0.3046
B9	3358 #D20	2.9	50000	0.2276
B10	3358 #D21	1.0	50000	0.3870
B11	3358 #D22	0.3	50000	0.3725
B12	SampB12			

 Copyright 2015 Advanced Analytical Technologies, Inc.

Filename and Data Path: Z:\project_data\LIMS_projects\3358_He_Quan_Sun_10X_Genomics_for_recombination_break_identification_MPIPZ\Public\17-10-46\2017 12 13 17H 10M.raw

B1: 3358 #D12
GQN: 0.0

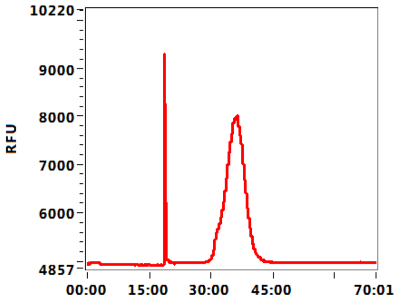

B2: 3358 #D13
GQN: 0.0

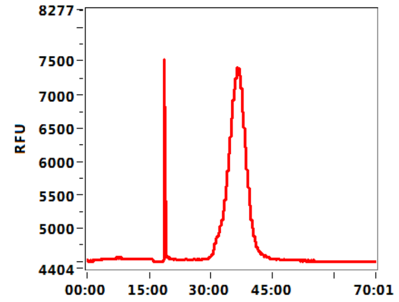

B3: 3358 #D14
GQN: 0.0

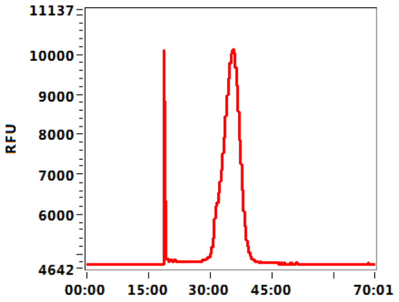

B4: 3358 #D15
GQN: 0.0

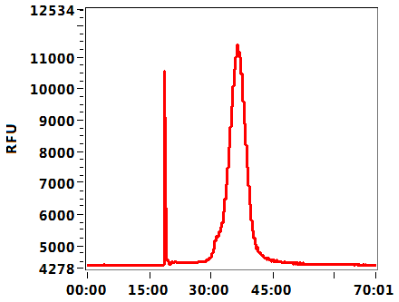

B5: 3358 #D16
GQN: 0.6

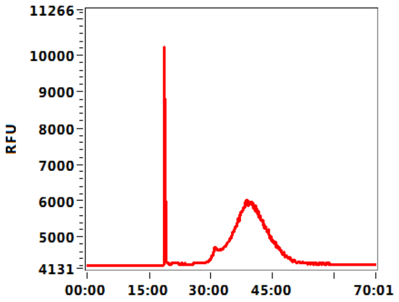

B6: 3358 #D17
GQN: 1.1

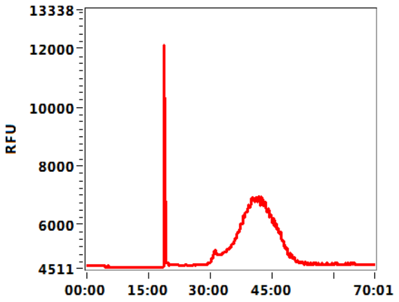

B7: 3358 #D18
GQN: 0.9

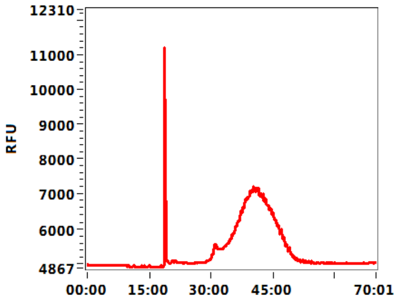

B8: 3358 #D19
GQN: 0.5

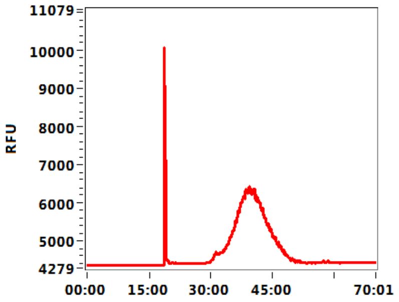

B9: 3358 #D20
GQN: 2.9

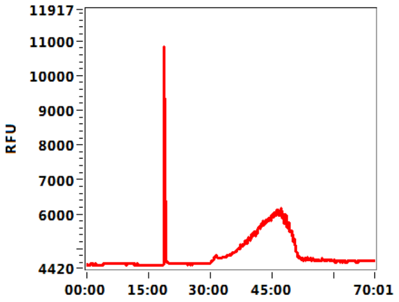

B10: 3358 #D21
GQN: 1.0

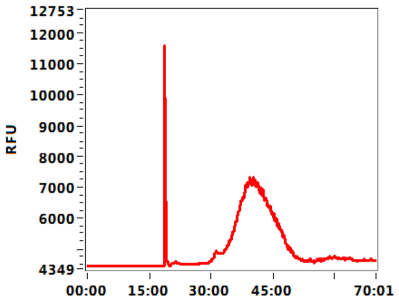

B11: 3358 #D22
GQN: 0.3

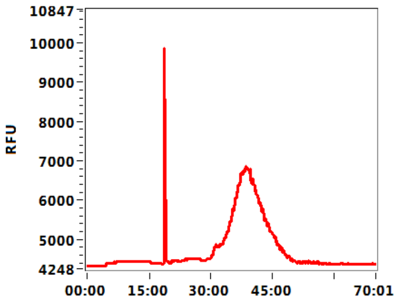

B12: SampB12

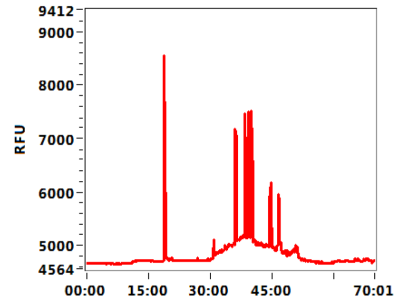

Copyright 2015 Advanced Analytical Technologies, Inc.

Sample: 3358 #D12
Well Location: B1
Created: 13-Dec-17 5:27 PM

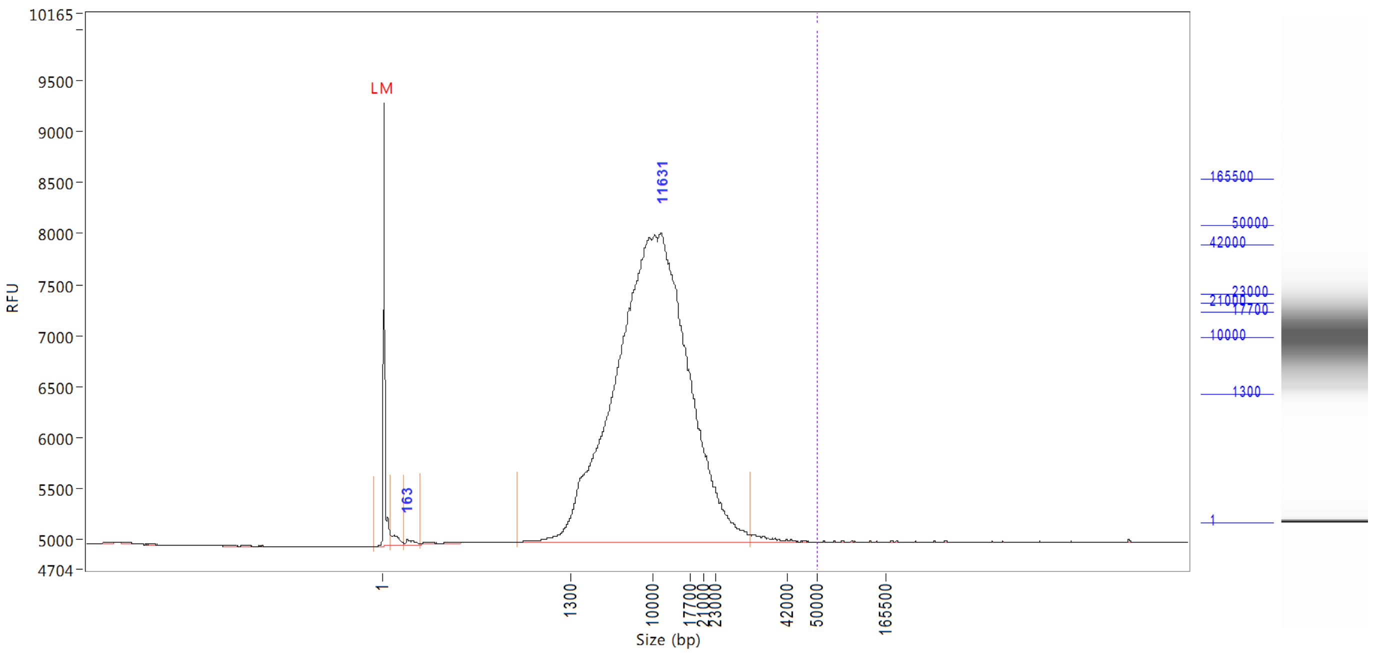

Peak	Size (bp)	Conc. (ng/uL)	From (bp)	To (bp)	Avg. Size (bp)	CV%	RFU	Corr. Peak Area
1	1 (LM)	0.0025	0	54	4	330.03	4359	27.202
2	163	0.0013	139	255	186	15.24	56	1.444
3	11631	0.4295	926	32154	10569	51.46	3043	462.763

TIC:	0.4308	ng/uL
TIM:	0.0787	nmole/L
Total Conc.:	0.4380	ng/uL

GQN:	0.0
Threshold:	50000

Sample Peak Width (sec): 20 Sample Min Peak Height: 50 Sample Baseline V to V?: Y Sample Baseline V to V pts: 3
Sample Filter: Binomial # of Pts for Filter: 3 Sample Start Region (min): 0 Sample End Region (min): 70
Manual Baseline Start (min): 25 Manual Baseline End (min): 70
Marker Peak Width (sec): 8 Marker Min Peak Height: 500 Marker Baseline V to V?: N Marker Baseline V to V pts: 3
Lower Marker Selection: First Peak > 500 RFU Upper Marker Selection: Last Peak > 500 RFU
Ladder Size (bp)1, 1300, 10000, 17700, 21000, 23000, 42000, 50000, 165500
Quantification Using: Ladder Final Concentration (ng/uL): 0.0200 Dilution Factor: 10.0
Min. RFU for Data Processing: 1 Size Threshold (b.p.): 50000

 Copyright 2015 Advanced Analytical Technologies, Inc.

Sample: 3358 #D13
Well Location: B2
Created: 13-Dec-17 5:27 PM

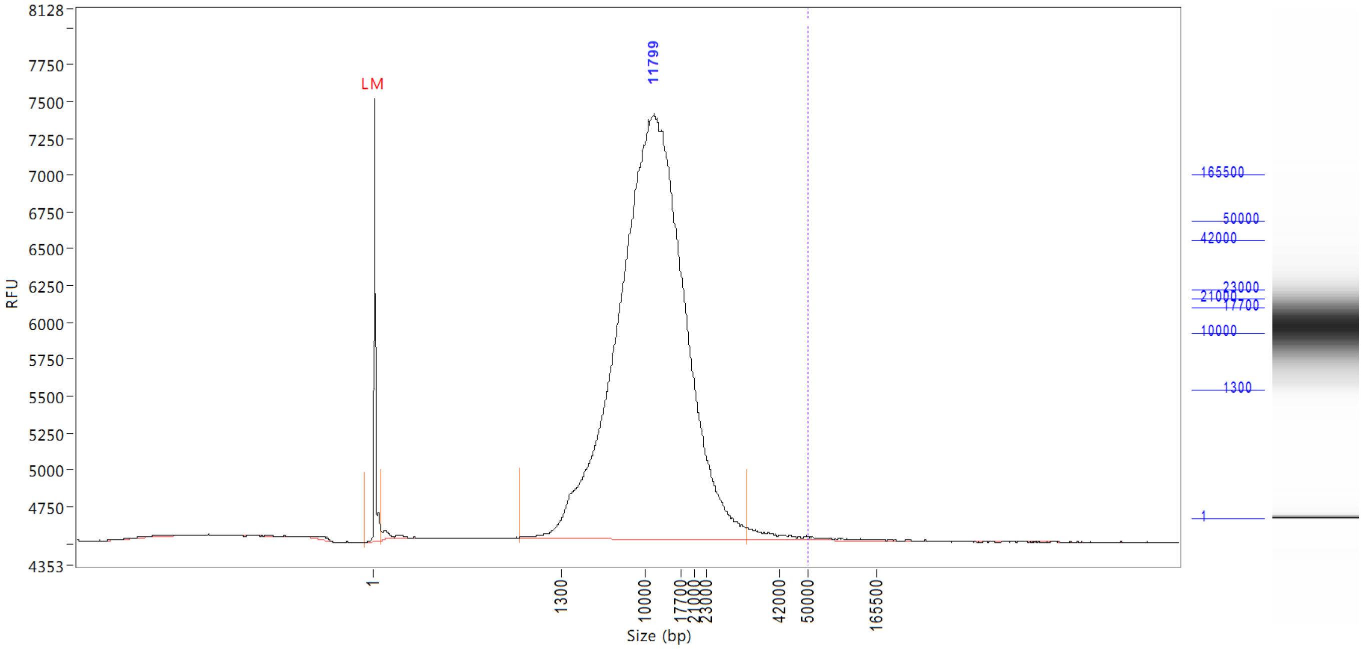

Peak	Size (bp)	Conc. (ng/uL)	From (bp)	To (bp)	Avg. Size (bp)	CV%	RFU	Corr. Peak Area
1	1 (LM)	0.0025	0	51	2	527.65	3012	18.695
2	11799	0.5315	1009	33679	11815	47.19	2885	393.602

TIC:	0.5315	ng/uL
TIM:	0.0741	nmole/L
Total Conc.:	0.5403	ng/uL
GQN:	0.0	
Threshold:	50000	

Sample Peak Width (sec): 20   Sample Min Peak Height: 50   Sample Baseline V to V?: Y   Sample Baseline V to V pts: 3
Sample Filter: Binomial   # of Pts for Filter: 3   Sample Start Region (min): 0   Sample End Region (min): 70
Manual Baseline Start (min): 25   Manual Baseline End (min): 70
Marker Peak Width (sec): 8   Marker Min Peak Height: 500   Marker Baseline V to V?: N   Marker Baseline V to V pts: 3
Lower Marker Selection: First Peak > 500 RFU   Upper Marker Selection: Last Peak > 500 RFU
Ladder Size (bp)1, 1300, 10000, 17700, 21000, 23000, 42000, 50000, 165500
Quantification Using: Ladder   Final Concentration (ng/uL): 0.0200   Dilution Factor: 10.0
Min. RFU for Data Processing: 1   Size Threshold (b.p.): 50000

 Copyright 2015 Advanced Analytical Technologies, Inc.

Sample: 3358 #D14
Well Location: B3
Created: 13-Dec-17 5:27 PM

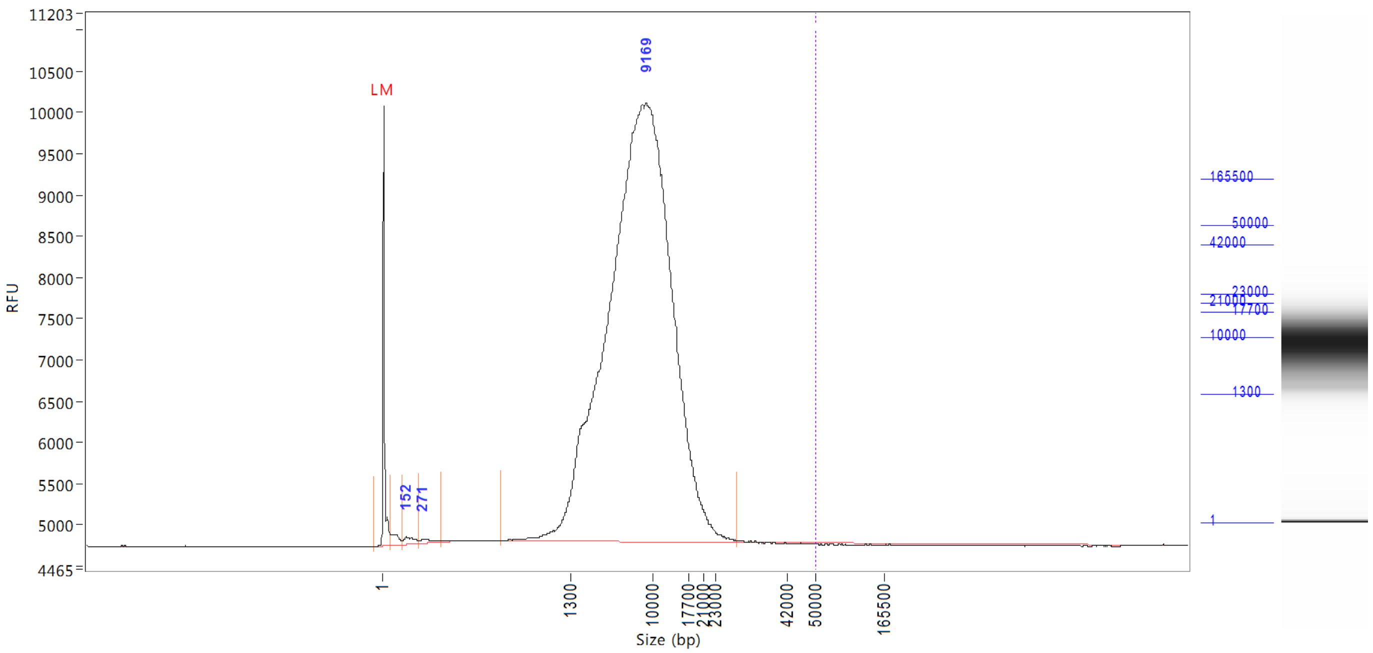

Peak	Size (bp)	Conc. (ng/uL)	From (bp)	To (bp)	Avg. Size (bp)	CV%	RFU	Corr. Peak Area
1	1 (LM)	0.0025	0	47	3	426.22	5333	32.951
2	152	0.0024	134	244	182	16.37	92	3.109
3	271	0.0017	244	405	309	13.81	55	2.172
4	9169	0.5526	817	28617	8643	47.61	5326	721.261

TIC:	0.5567	ng/uL
TIM:	0.1356	nmole/L
Total Conc.:	0.5605	ng/uL
GQN:	0.0	
Threshold:	50000	

Sample Peak Width (sec): 20 Sample Min Peak Height: 50 Sample Baseline V to V?: Y Sample Baseline V to V pts: 3
Sample Filter: Binomial # of Pts for Filter: 3 Sample Start Region (min): 0 Sample End Region (min): 70
Manual Baseline Start (min): 25 Manual Baseline End (min): 70
Marker Peak Width (sec): 8 Marker Min Peak Height: 500 Marker Baseline V to V?: N Marker Baseline V to V pts: 3
Lower Marker Selection: First Peak > 500 RFU Upper Marker Selection: Last Peak > 500 RFU
Ladder Size (bp)1, 1300, 10000, 17700, 21000, 23000, 42000, 50000, 165500
Quantification Using: Ladder Final Concentration (ng/uL): 0.0200 Dilution Factor: 10.0
Min. RFU for Data Processing: 1 Size Threshold (b.p.): 50000

 Copyright 2015 Advanced Analytical Technologies, Inc.

Sample: 3358 #D15
Well Location: B4
Created: 13-Dec-17 5:27 PM

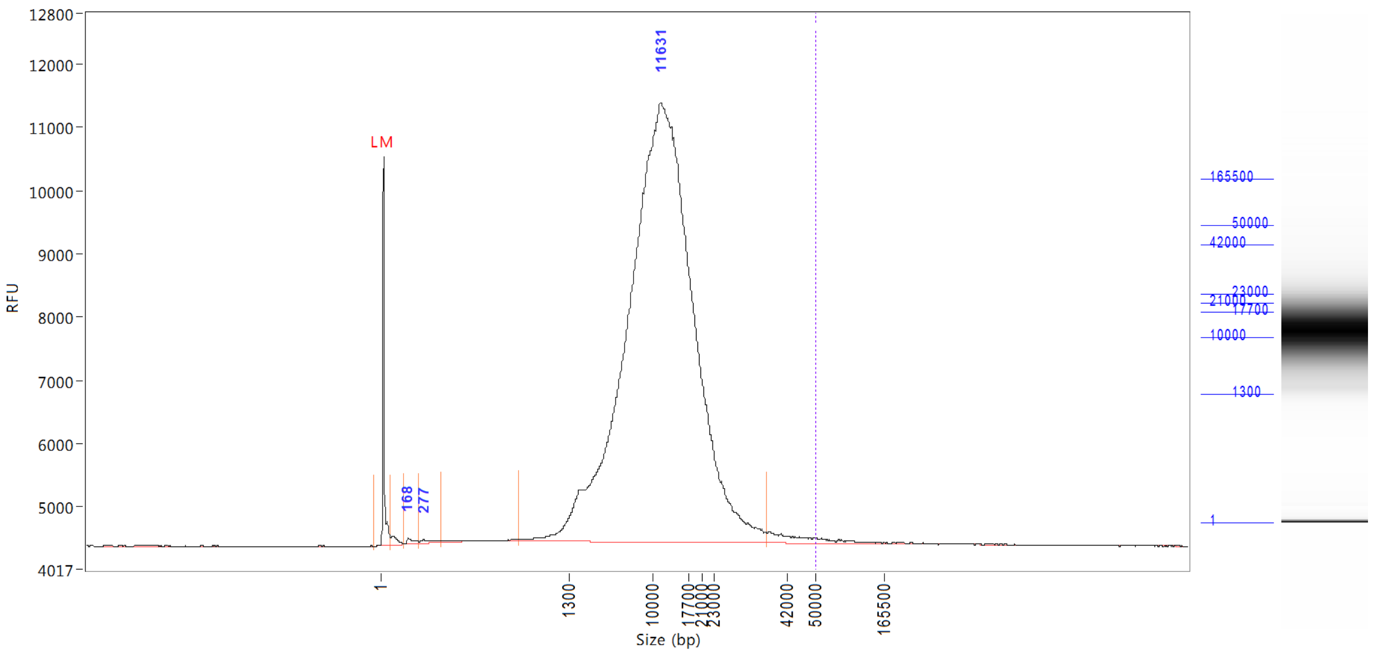

Peak	Size (bp)	Conc. (ng/uL)	From (bp)	To (bp)	Avg. Size (bp)	CV%	RFU	Corr. Peak Area
1	1 (LM)	0.0025	0	49	3	375.70	6144	37.350
2	168	0.0015	148	250	195	13.12	100	2.238
3	277	0.0015	250	407	315	13.45	54	2.181
4	11631	0.6299	944	36522	12047	48.44	6949	931.897

TIC:	0.6329	ng/uL
TIM:	0.1065	nmole/L
Total Conc.:	0.6443	ng/uL
GQN:	0.0	
Threshold:	50000	

Sample Peak Width (sec): 20   Sample Min Peak Height: 50   Sample Baseline V to V?: Y   Sample Baseline V to V pts: 3
Sample Filter: Binomial   # of Pts for Filter: 3   Sample Start Region (min): 0   Sample End Region (min): 70
Manual Baseline Start (min): 25   Manual Baseline End (min): 70
Marker Peak Width (sec): 8   Marker Min Peak Height: 500   Marker Baseline V to V?: N   Marker Baseline V to V pts: 3
Lower Marker Selection: First Peak > 500 RFU   Upper Marker Selection: Last Peak > 500 RFU
Ladder Size (bp)1, 1300, 10000, 17700, 21000, 23000, 42000, 50000, 165500
Quantification Using: Ladder   Final Concentration (ng/uL): 0.0200   Dilution Factor: 10.0
Min. RFU for Data Processing: 1   Size Threshold (b.p.): 50000

 Copyright 2015 Advanced Analytical Technologies, Inc.

Sample: 3358 #D16
Well Location: B5
Created: 13-Dec-17 5:27 PM

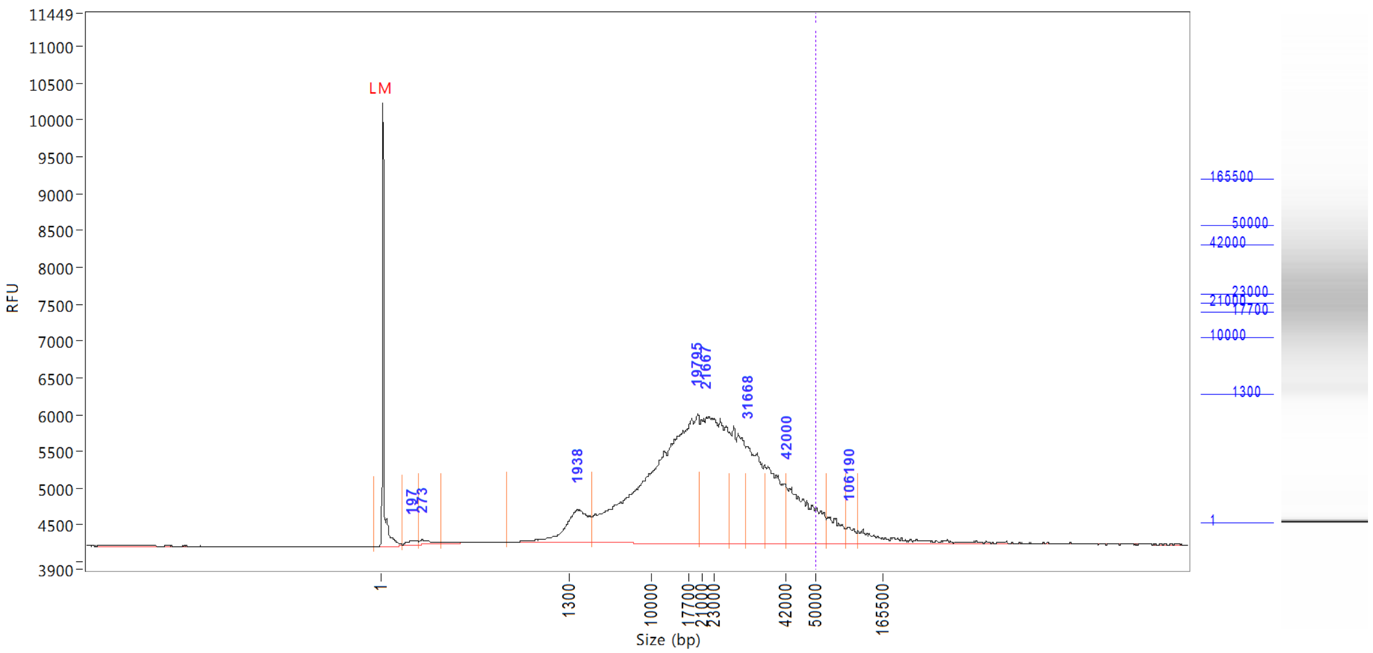

Peak	Size (bp)	Conc. (ng/uL)	From (bp)	To (bp)	Avg. Size (bp)	CV%	RFU	Corr. Peak Area
1	1 (LM)	0.0025	0	134	6	336.17	6033	39.851
2	197	0.0013	134	248	197	15.24	54	2.077
3	273	0.0015	248	403	307	13.38	63	2.302
4	1938	0.0173	866	3600	1975	39.91	453	27.261
5	19795	0.1130	3600	20366	12379	38.19	1754	178.362
6	21667	0.0505	20366	27161	23377	8.25	1722	79.656
7	31668	0.0226	31183	36522	33723	4.61	1318	35.687
8	42000	0.0199	42000	69622	48503	13.46	778	31.397
9	106190	0.0020	101730	123136	111726	5.59	224	3.086

TIC:	0.2280	ng/uL
TIM:	0.0536	nmole/L
Total Conc.:	0.2766	ng/uL
GQN:	0.6	
Threshold:	50000	

```
Sample Peak Width (sec): 20   Sample Min Peak Height: 50    Sample Baseline V to V?: Y    Sample Baseline V to V pts: 3
Sample Filter: Binomial       # of Pts for Filter: 3        Sample Start Region (min): 0  Sample End Region (min): 70
Manual Baseline Start (min): 25     Manual Baseline End (min): 70
Marker Peak Width (sec): 8    Marker Min Peak Height: 500   Marker Baseline V to V?: N    Marker Baseline V to V pts: 3
Lower Marker Selection: First Peak > 500 RFU                Upper Marker Selection: Last Peak > 500 RFU
Ladder Size (bp)1, 1300, 10000, 17700, 21000, 23000, 42000, 50000, 165500
Quantification Using: Ladder          Final Concentration (ng/uL): 0.0200          Dilution Factor: 10.0
Min. RFU for Data Processing: 1      Size Threshold (b.p.): 50000
```

 Copyright 2015 Advanced Analytical Technologies, Inc.

Sample: 3358 #D17
Well Location: B6
Created: 13-Dec-17 5:27 PM

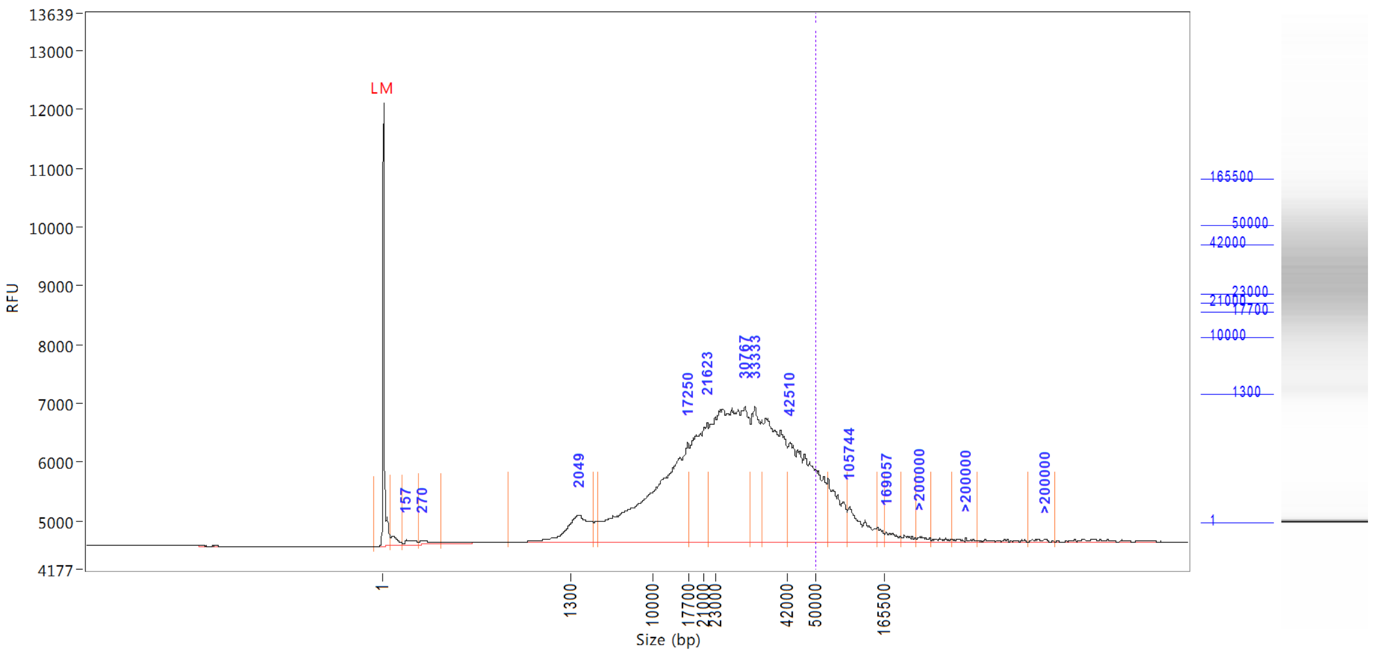

Peak	Size (bp)	Conc. (ng/uL)	From (bp)	To (bp)	Avg. Size (bp)	CV%	RFU	Corr. Peak Area
1	1 (LM)	0.0025	0	51	2	601.74	7555	47.354
2	157	0.0017	138	248	190	15.73	90	3.209
3	270	0.0013	248	400	304	12.99	73	2.487
4	2049	0.0148	868	3656	1986	40.17	475	27.781
5	17250	0.0714	4127	17700	11337	34.34	1703	133.957
6	21623	0.0311	17700	21845	19945	6.31	2044	58.268
7	30767	0.0761	21845	32292	26765	11.62	2319	142.704
8	33333	0.0200	32292	35343	33791	2.65	2313	37.560
9	42510	0.0415	41931	70068	48876	14.06	1699	77.921
10	105744	0.0072	103068	153460	123661	11.77	600	13.527
11	169057	0.0013	166833	193511	178802	4.29	173	2.493
12	>200000	0.0007	>200000	>200000	>200000	3.13	94	1.240
13	>200000	0.0006	>200000	>200000	>200000	4.01	65	1.068
14	>200000	0.0004	>200000	>200000	>200000	2.59	48	0.660

TIC:	0.2681	ng/uL
TIM:	0.0544	nmole/L
Total Conc.:	0.3218	ng/uL

GQN:	1.1
Threshold:	50000

```
Sample Peak Width (sec): 20   Sample Min Peak Height: 50    Sample Baseline V to V?: Y    Sample Baseline V to V pts: 3
Sample Filter: Binomial       # of Pts for Filter: 3        Sample Start Region (min): 0  Sample End Region (min): 70
Manual Baseline Start (min): 25     Manual Baseline End (min): 70
Marker Peak Width (sec): 8    Marker Min Peak Height: 500   Marker Baseline V to V?: N    Marker Baseline V to V pts: 3
Lower Marker Selection: First Peak > 500 RFU                Upper Marker Selection: Last Peak > 500 RFU
Ladder Size (bp)1, 1300, 10000, 17700, 21000, 23000, 42000, 50000, 165500
Quantification Using: Ladder          Final Concentration (ng/uL): 0.0200          Dilution Factor: 10.0
Min. RFU for Data Processing: 1      Size Threshold (b.p.): 50000
```

 Copyright 2015 Advanced Analytical Technologies, Inc.

Sample: 3358 #D18
Well Location: B7
Created: 13-Dec-17 5:27 PM

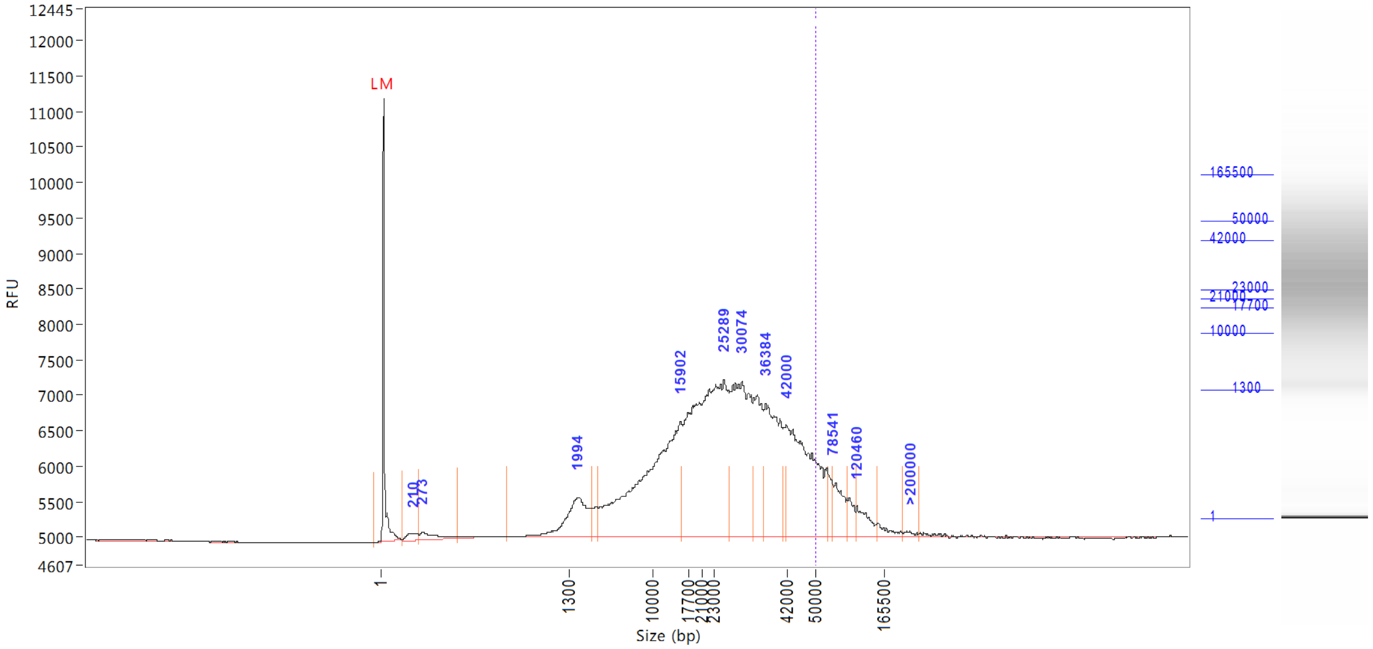

Peak	Size (bp)	Conc. (ng/uL)	From (bp)	To (bp)	Avg. Size (bp)	CV%	RFU	Corr. Peak Area
1	1 (LM)	0.0025	0	130	7	276.68	6253	40.821
2	210	0.0025	130	246	194	15.53	97	3.985
3	273	0.0037	246	514	341	20.99	117	5.920
4	1994	0.0188	855	3600	1994	38.73	554	30.448
5	15902	0.0806	4182	16295	10463	32.69	1628	130.403
6	25289	0.0907	16295	26745	21537	13.43	2210	146.704
7	30074	0.0469	26745	33055	29839	6.11	2183	75.811
8	36384	0.0315	35829	41307	38462	4.15	1879	50.908
9	42000	0.0424	42000	69622	48804	13.85	1566	68.615
10	78541	0.0081	78541	104852	90670	8.43	774	13.060
11	120460	0.0046	119122	153906	133690	7.31	437	7.428
12	>200000	0.0006	199291	>200000	>200000	3.65	77	0.910

TIC:	0.3304	ng/uL
TIM:	0.0793	nmole/L
Total Conc.:	0.3624	ng/uL

GQN:	0.9
Threshold:	50000

```
Sample Peak Width (sec): 20   Sample Min Peak Height: 50    Sample Baseline V to V?: Y    Sample Baseline V to V pts: 3
Sample Filter: Binomial       # of Pts for Filter: 3        Sample Start Region (min): 0  Sample End Region (min): 70
Manual Baseline Start (min): 25    Manual Baseline End (min): 70
Marker Peak Width (sec): 8    Marker Min Peak Height: 500   Marker Baseline V to V?: N    Marker Baseline V to V pts: 3
Lower Marker Selection: First Peak > 500 RFU                Upper Marker Selection: Last Peak > 500 RFU
Ladder Size (bp)1, 1300, 10000, 17700, 21000, 23000, 42000, 50000, 165500
Quantification Using: Ladder          Final Concentration (ng/uL): 0.0200          Dilution Factor: 10.0
Min. RFU for Data Processing: 1      Size Threshold (b.p.): 50000
```

 Copyright 2015 Advanced Analytical Technologies, Inc.

Sample: 3358 #D19
Well Location: B8
Created: 13-Dec-17 5:27 PM

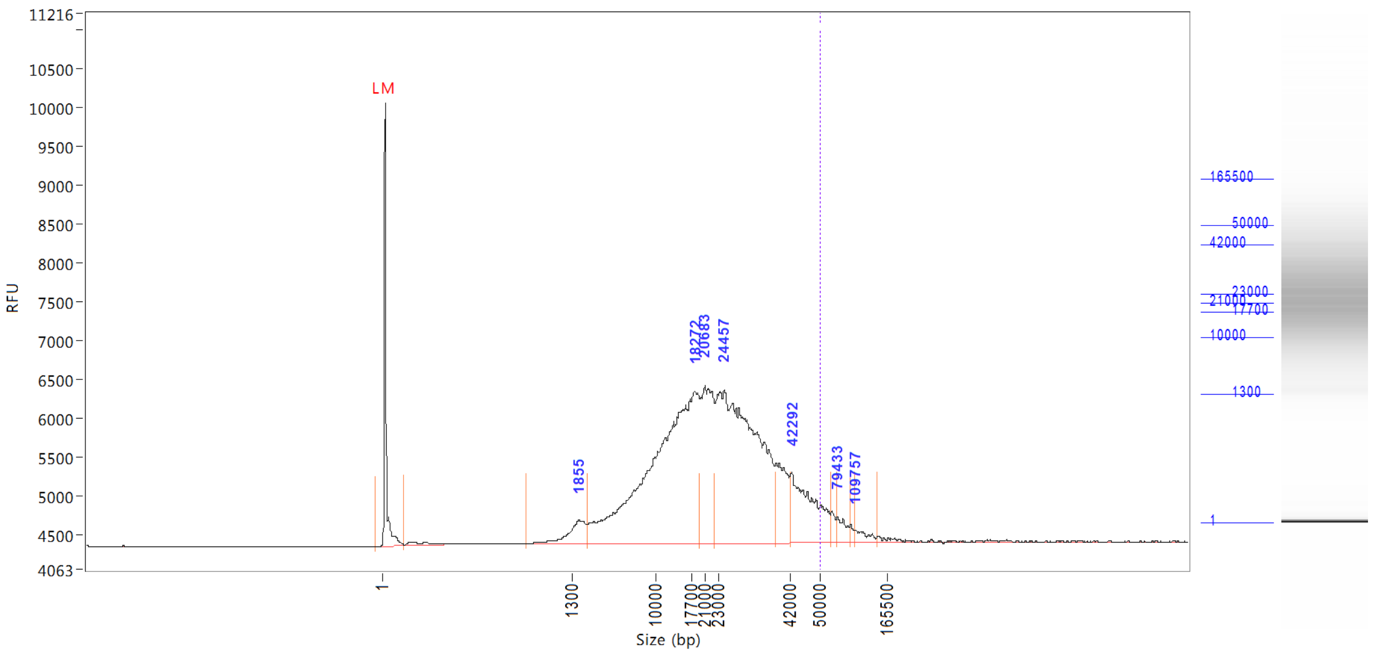

Peak	Size (bp)	Conc. (ng/uL)	From (bp)	To (bp)	Avg. Size (bp)	CV%	RFU	Corr. Peak Area
1	1 (LM)	0.0025	0	136	8	266.09	5715	38.103
2	1855	0.0083	975	2714	1681	29.50	308	12.602
3	18272	0.1229	2714	19350	12052	37.02	1962	185.419
4	20683	0.0318	19350	22378	20991	4.14	2026	47.964
5	24457	0.0962	22378	38256	29174	15.63	1966	145.258
6	42292	0.0220	42000	70068	48498	13.77	907	33.135
7	79433	0.0030	78541	100838	88722	7.44	339	4.504
8	109757	0.0020	109757	146770	125454	8.37	161	3.061

TIC:	0.2862	ng/uL
TIM:	0.0337	nmole/L
Total Conc.:	0.3046	ng/uL
GQN:	0.5	
Threshold:	50000	

Sample Peak Width (sec): 20 Sample Min Peak Height: 50 Sample Baseline V to V?: Y Sample Baseline V to V pts: 3
Sample Filter: Binomial # of Pts for Filter: 3 Sample Start Region (min): 0 Sample End Region (min): 70
Manual Baseline Start (min): 25 Manual Baseline End (min): 70
Marker Peak Width (sec): 8 Marker Min Peak Height: 500 Marker Baseline V to V?: N Marker Baseline V to V pts: 3
Lower Marker Selection: First Peak > 500 RFU Upper Marker Selection: Last Peak > 500 RFU
Ladder Size (bp)1, 1300, 10000, 17700, 21000, 23000, 42000, 50000, 165500
Quantification Using: Ladder Final Concentration (ng/uL): 0.0200 Dilution Factor: 10.0
Min. RFU for Data Processing: 1 Size Threshold (b.p.): 50000

 Copyright 2015 Advanced Analytical Technologies, Inc.

Sample: 3358 #D20
Well Location: B9
Created: 13-Dec-17 5:27 PM

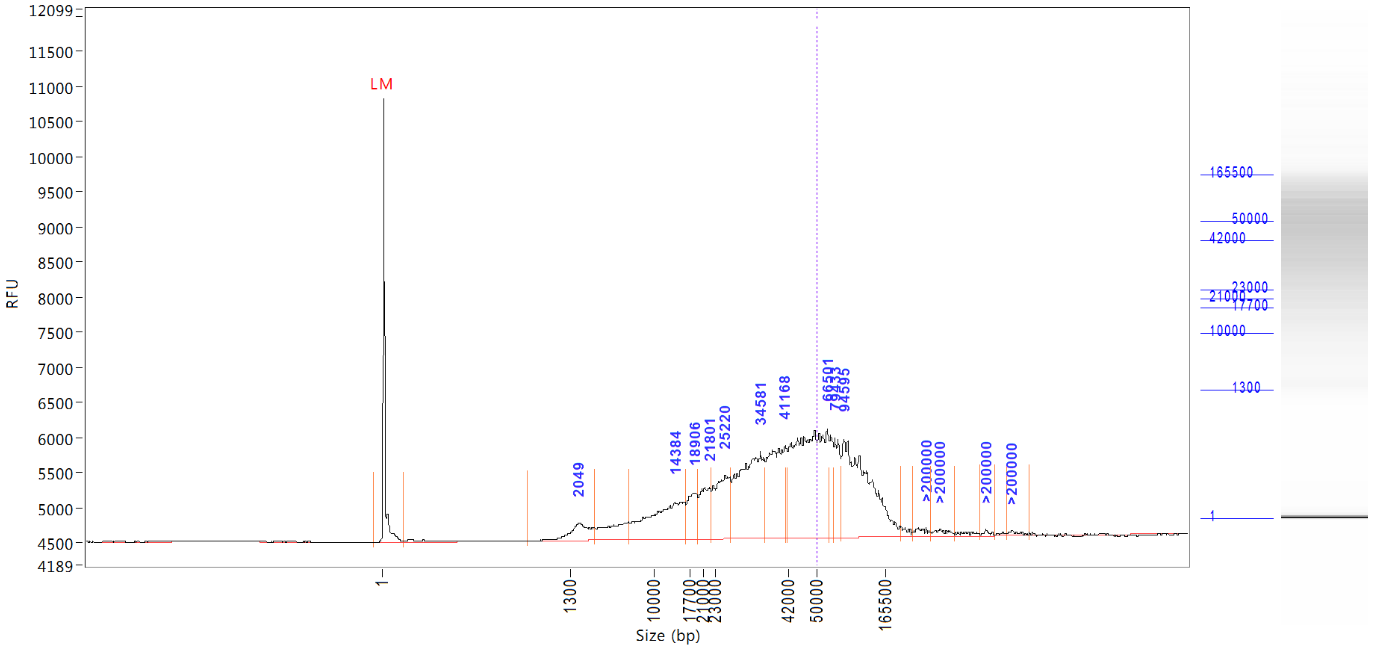

Peak	Size (bp)	Conc. (ng/uL)	From (bp)	To (bp)	Avg. Size (bp)	CV%	RFU	Corr. Peak Area
1	1 (LM)	0.0025	0	138	7	306.39	6326	41.548
2	2049	0.0071	1000	3739	2171	36.76	238	11.681
3	14384	0.0229	7368	16745	11909	23.55	536	37.659
4	18906	0.0077	16745	19541	18130	4.45	660	12.609
5	21801	0.0090	19541	22156	20984	3.57	726	14.856
6	25220	0.0154	22156	26815	24375	5.74	894	25.271
7	34581	0.0325	26815	35967	31600	8.30	1227	53.497
8	41168	0.0222	35967	41376	38705	4.08	1310	36.525
9	66501	0.0480	42000	69622	49996	14.55	1550	78.992
10	79433	0.0074	78095	89690	83694	4.11	1425	12.120
11	94595	0.0346	89690	192621	125112	19.81	1404	56.908
12	>200000	0.0012	>200000	>200000	>200000	3.83	128	1.931
13	>200000	0.0011	>200000	>200000	>200000	4.06	101	1.804
14	>200000	0.0004	>200000	>200000	>200000	1.87	88	0.663
15	>200000	0.0005	>200000	>200000	>200000	2.65	73	0.839

TIC:	0.2098	ng/uL
TIM:	0.0158	nmole/L
Total Conc.:	0.2276	ng/uL
GQN:	2.9	
Threshold:	50000	

```
Sample Peak Width (sec): 20   Sample Min Peak Height: 50    Sample Baseline V to V?: Y    Sample Baseline V to V pts: 3
Sample Filter: Binomial       # of Pts for Filter: 3        Sample Start Region (min): 0  Sample End Region (min): 70
Manual Baseline Start (min): 25     Manual Baseline End (min): 70
Marker Peak Width (sec): 8    Marker Min Peak Height: 500   Marker Baseline V to V?: N    Marker Baseline V to V pts: 3
Lower Marker Selection: First Peak > 500 RFU                Upper Marker Selection: Last Peak > 500 RFU
Ladder Size (bp)1, 1300, 10000, 17700, 21000, 23000, 42000, 50000, 165500
Quantification Using: Ladder          Final Concentration (ng/uL): 0.0200          Dilution Factor: 10.0
Min. RFU for Data Processing: 1      Size Threshold (b.p.): 50000
```

 Copyright 2015 Advanced Analytical Technologies, Inc.

Sample: 3358 #D21
Well Location: B10
Created: 13-Dec-17 5:27 PM

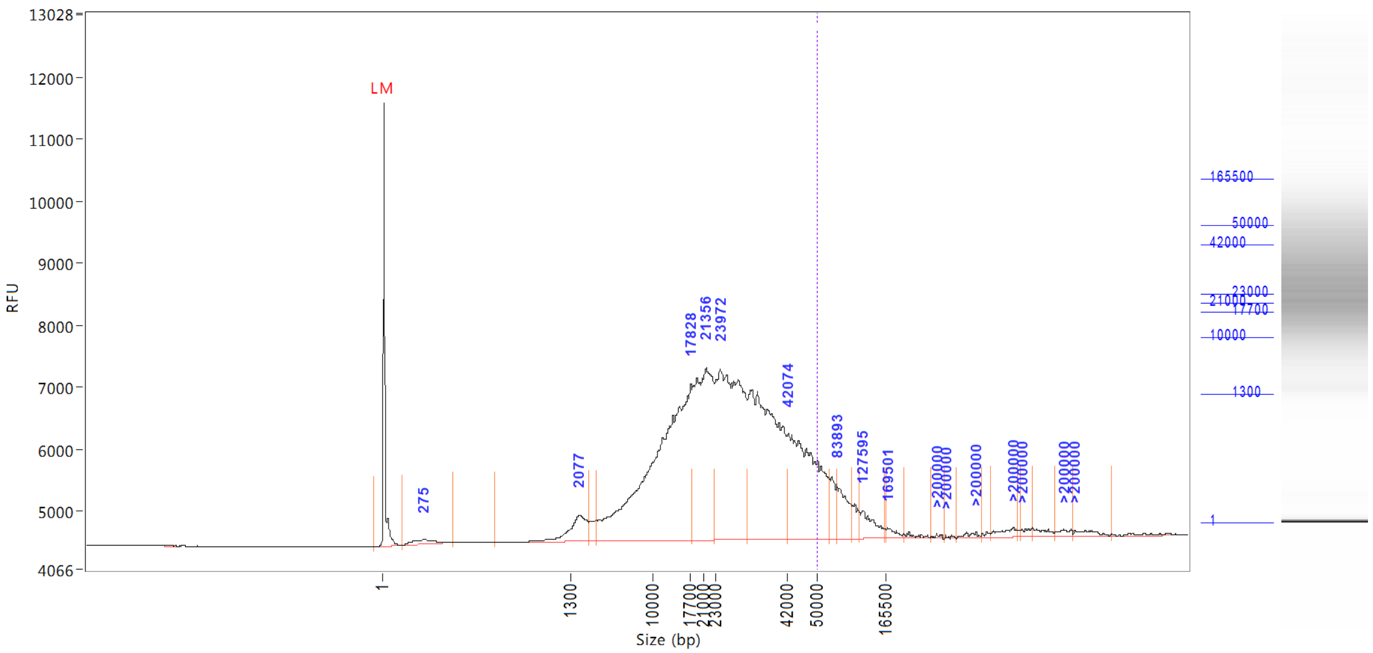

Peak	Size (bp)	Conc. (ng/uL)	From (bp)	To (bp)	Avg. Size (bp)	CV%	RFU	Corr. Peak Area
1	1 (LM)	0.0025	0	130	5	318.18	7159	45.594
2	275	0.0029	130	483	273	26.10	82	5.241
3	2077	0.0097	770	3074	1885	32.37	423	17.466
4	17828	0.1065	3961	18018	11889	32.43	2523	192.340
5	21356	0.0532	18018	22689	20569	6.63	2774	96.075
6	23972	0.0725	22689	31252	26732	9.45	2759	130.975
7	42074	0.0429	42000	70068	48909	13.92	1701	77.445
8	83893	0.0081	83001	110203	95387	8.30	874	14.555
9	127595	0.0049	123136	164162	139815	8.20	454	8.845
10	169501	0.0013	167278	197512	179424	4.75	167	2.280
11	>200000	0.0002	>200000	>200000	>200000	2.11	77	0.392
12	>200000	0.0001	>200000	>200000	>200000	1.86	39	0.155
13	>200000	0.0006	>200000	>200000	>200000	3.34	83	1.076
14	>200000	0.0016	>200000	>200000	>200000	3.58	159	2.886
15	>200000	0.0008	>200000	>200000	>200000	1.48	133	1.403
16	>200000	0.0009	>200000	>200000	>200000	1.88	116	1.687
17	>200000	0.0012	>200000	>200000	>200000	3.03	105	2.194

TIC:	0.3073	ng/uL
TIM:	0.0511	nmole/L
Total Conc.:	0.3870	ng/uL

GQN:	1.0
Threshold:	50000

Sample Peak Width (sec): 20   Sample Min Peak Height: 50   Sample Baseline V to V?: Y   Sample Baseline V to V pts: 3
Sample Filter: Binomial   # of Pts for Filter: 3   Sample Start Region (min): 0   Sample End Region (min): 70
Manual Baseline Start (min): 25   Manual Baseline End (min): 70
Marker Peak Width (sec): 8   Marker Min Peak Height: 500   Marker Baseline V to V?: N   Marker Baseline V to V pts: 3
Lower Marker Selection: First Peak > 500 RFU   Upper Marker Selection: Last Peak > 500 RFU
Ladder Size (bp)1, 1300, 10000, 17700, 21000, 23000, 42000, 50000, 165500
Quantification Using: Ladder   Final Concentration (ng/uL): 0.0200   Dilution Factor: 10.0
Min. RFU for Data Processing: 1   Size Threshold (b.p.): 50000

 Copyright 2015 Advanced Analytical Technologies, Inc.

Sample: 3358 #D22
Well Location: B11
Created: 13-Dec-17 5:27 PM

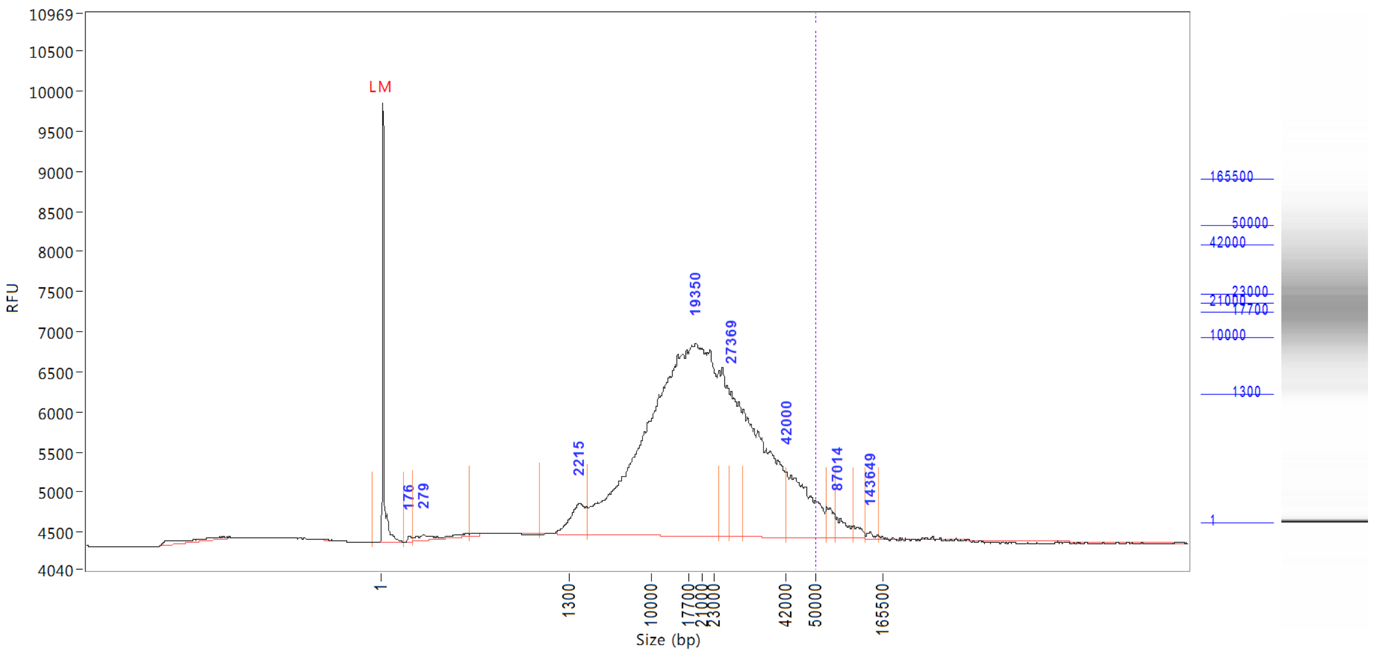

Peak	Size (bp)	Conc. (ng/uL)	From (bp)	To (bp)	Avg. Size (bp)	CV%	RFU	Corr. Peak Area
1	1 (LM)	0.0025	0	148	7	301.81	5489	35.970
2	176	0.0007	148	206	184	6.80	70	1.064
3	279	0.0040	206	602	371	32.68	71	5.700
4	2215	0.0108	1085	3185	2062	30.27	393	15.390
5	19350	0.2267	3185	24110	14538	38.99	2403	322.959
6	27369	0.0266	26884	30698	28736	3.90	1802	37.845
7	42000	0.0231	41931	69622	48134	13.12	816	32.854
8	87014	0.0029	85676	115554	98669	8.81	265	4.202
9	143649	0.0005	134730	158365	145418	4.49	77	0.767
10	0	NaN	0	0	>200000	NaN	0	NaN

TIC:	NaN	ng/uL
TIM:	NaN	nmole/L
Total Conc.:	0.3725	ng/uL
GQN:	0.3	
Threshold:	50000	

```
Sample Peak Width (sec): 20   Sample Min Peak Height: 50    Sample Baseline V to V?: Y    Sample Baseline V to V pts: 3
Sample Filter: Binomial       # of Pts for Filter: 3        Sample Start Region (min): 0  Sample End Region (min): 70
Manual Baseline Start (min): 25     Manual Baseline End (min): 70
Marker Peak Width (sec): 8    Marker Min Peak Height: 500   Marker Baseline V to V?: N    Marker Baseline V to V pts: 3
Lower Marker Selection: First Peak > 500 RFU                Upper Marker Selection: Last Peak > 500 RFU
Ladder Size (bp)1, 1300, 10000, 17700, 21000, 23000, 42000, 50000, 165500
Quantification Using: Ladder          Final Concentration (ng/uL): 0.0200          Dilution Factor: 10.0
Min. RFU for Data Processing: 1      Size Threshold (b.p.): 50000
```

 Copyright 2015 Advanced Analytical Technologies, Inc.

Sample: SampB12
Well Location: B12
Created: 13-Dec-17 5:27 PM

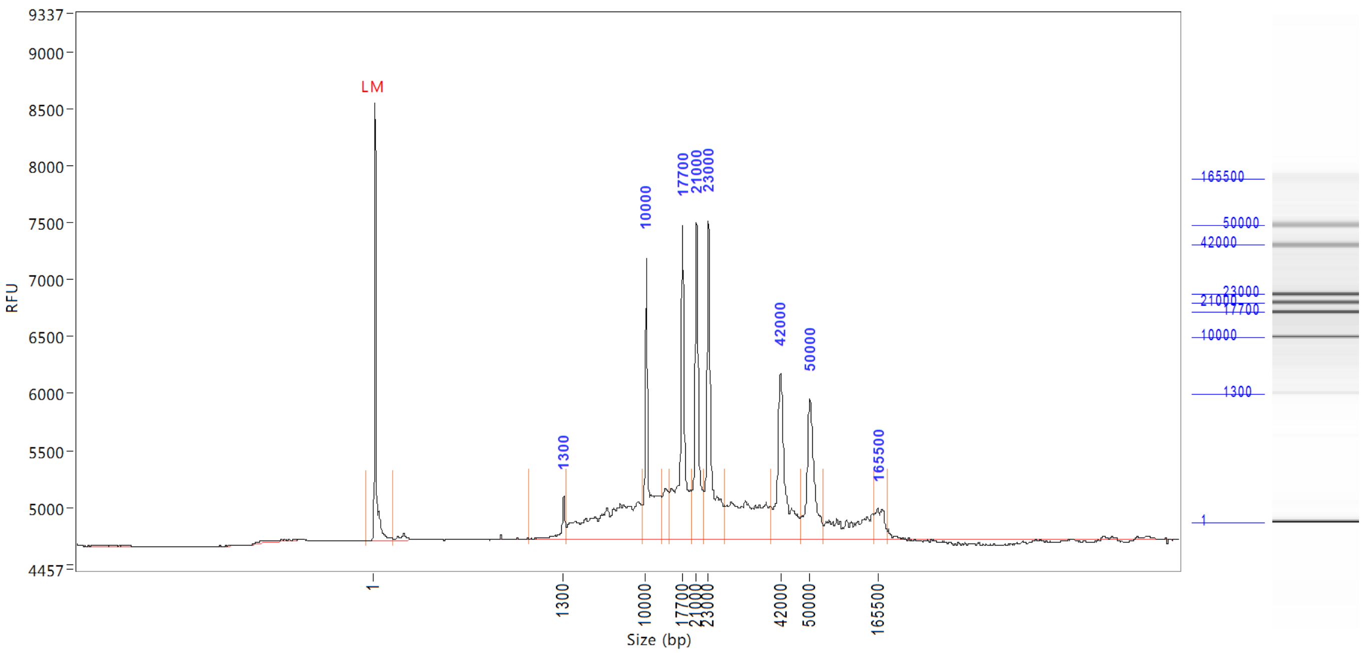

Peak	Size (bp)	Conc. (ng/uL)	From (bp)	To (bp)	Avg. Size (bp)	CV%	RFU	Corr. Peak Area
1	1 (LM)	0.0025	0	129	5	371.79	3851	25.243
2	1300	0.0036	1063	1550	1288	6.87	377	3.563
3	10000	0.0203	9557	13204	10747	9.93	2466	20.265
4	17700	0.0275	14834	19795	17399	6.46	2750	27.453
5	21000	0.0207	19795	22245	21025	2.41	2776	20.654
6	23000	0.0245	22245	27439	23747	5.63	2788	24.537
7	42000	0.0199	39365	47382	42586	4.20	1449	19.928
8	50000	0.0148	47382	72744	53420	10.82	1233	14.781
9	165500	0.0035	159257	182840	169251	3.60	273	3.546

TIC:	0.1347	ng/uL
TIM:	0.0148	nmole/L
Total Conc.:	0.2000	ng/uL

```
Sample Peak Width (sec): 20   Sample Min Peak Height: 300   Sample Baseline V to V?: Y    Sample Baseline V to V pts: 3
Sample Filter: Binomial       # of Pts for Filter: 3        Sample Start Region (min): 0  Sample End Region (min): 70
Manual Baseline Start (min): 25     Manual Baseline End (min): 70
Marker Peak Width (sec): 8    Marker Min Peak Height: 500   Marker Baseline V to V?: N    Marker Baseline V to V pts: 3
Lower Marker Selection: First Peak > 500 RFU                Upper Marker Selection: Last Peak > 500 RFU
Ladder Size (bp)1, 1300, 10000, 17700, 21000, 23000, 42000, 50000, 165500
Quantification Using: Ladder          Final Concentration (ng/uL): 0.0200          Dilution Factor: 10.0
Min. RFU for Data Processing: 1      Size Threshold (b.p.): 50000
```

 Copyright 2015 Advanced Analytical Technologies, Inc.

Sample: SampB12
Well Location: B12
Created: 13-Dec-17 5:27 PM
Fit Type: Point to Point

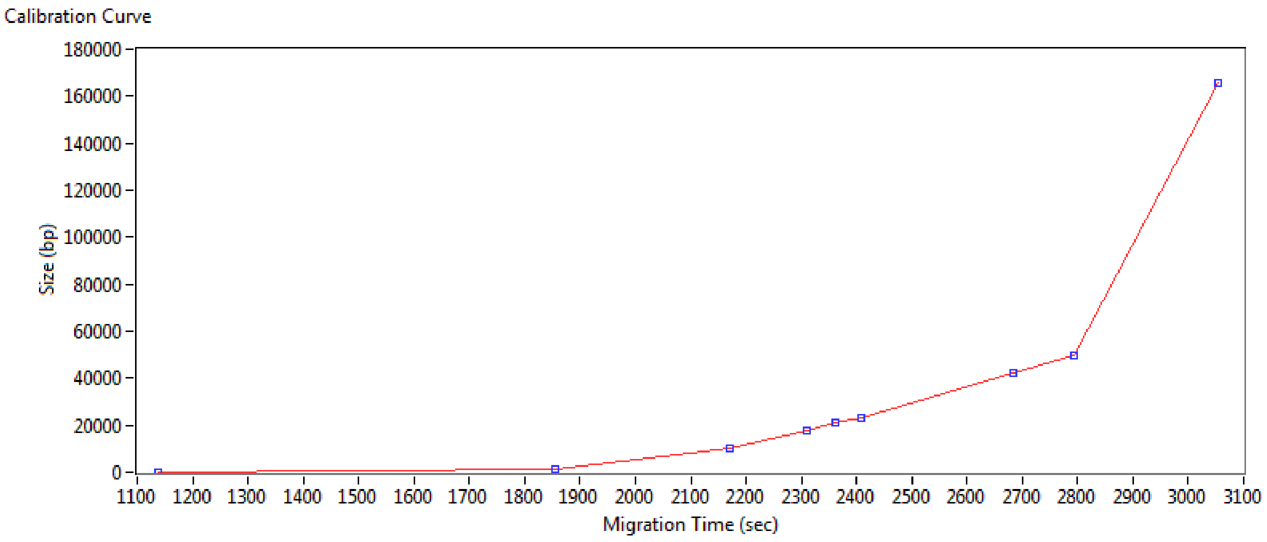

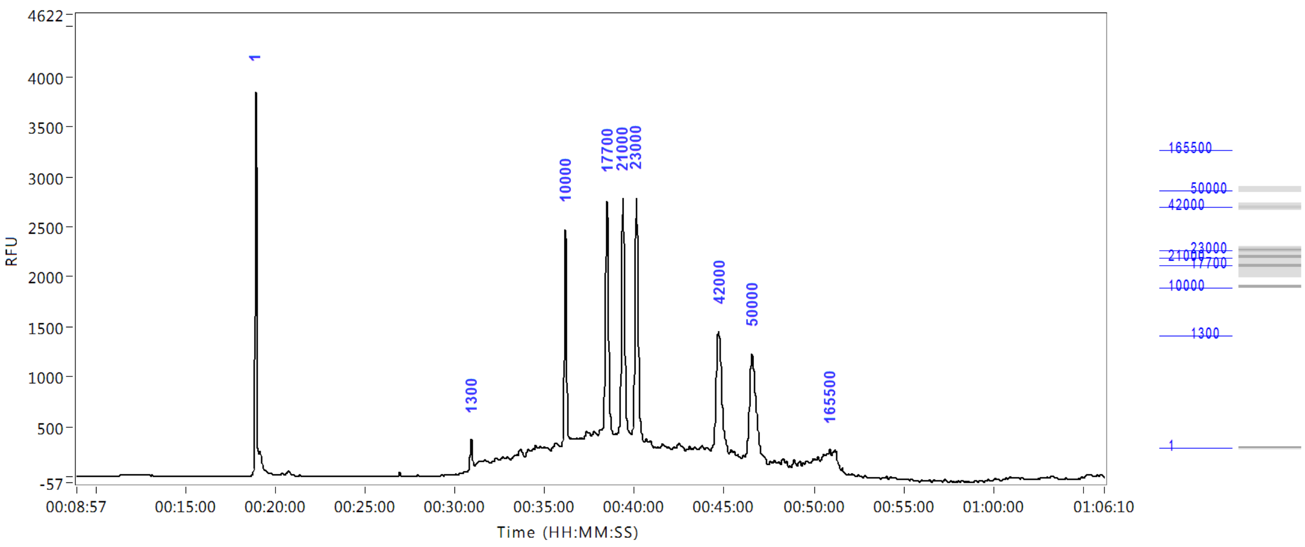

Supplement: Supplementary file 12 — Source Data [file 41467_2019_12209_MOESM12_ESM.zip › Source_Data/Source_Data_supplementary_Figure_4.zip!/Source_Data_supplementary_Figure_4/supplemenary_Figure_4_3358_FEMTO_D12-D22_Summary.pdf]
