# Supplementary material for: Linked-read sequencing of gametes allows efficient genome-wide analysis of meiotic recombination
Source: Nat Commun. 2019 Sep 20;10:4310. doi: 10.1038/s41467-019-12209-2 (PMC6754367; doi:10.1038/s41467-019-12209-2)
Supplement: Supplementary file 12 — Source Data [file 41467_2019_12209_MOESM12_ESM.zip › Source_Data/Source_Data_supplementary_Figure_4.zip!/Source_Data_supplementary_Figure_4/supplemenary_Figure_4_3358_FEMTO_D23-D30_Summary.pdf]

FEMTO Pulse Run Summary:

Filename and Data Path: Z:\project_data\LIMS_projects\
3358_He_Quan_Sun_10X_Genomics_for_recombination_break_identification_MPIPZ\
Public\18-38-12\2017 12 13 18H 38M.raw
Created: 13-Dec-17 6:55 PM
# of Capillaries: 12
Array Serial #: 041017-03USFP
Effect Length: 22 cm
Array Usage Count: 63
FEMTO Pulse Version #: 1.0.0.32
Device Serial #: F12119

METHOD INFORMATION
Method Name: FP-1002-22 - gDNA 165Kb.mthds
Gel Prime: No
Full Conditioning: Yes
Gel Prime to Buffer: No
Gel Selection: Gel 1
Perform Prerun: -5.0 kV, 60 sec.
Rinse: No
Marker 1: No
Rinse: Tray: 3, Row: A, # Dips: 1
Sample Injection: -5.0 kV, 30 sec.
Separation: 70.0 min.
Tray Name: Tray-1

Analysis Mode: gDNA

NOTES

Time Shift: Using configured: slope=0.00025, intercept=0.0

 Copyright 2015 Advanced Analytical Technologies, Inc.

Gel Image

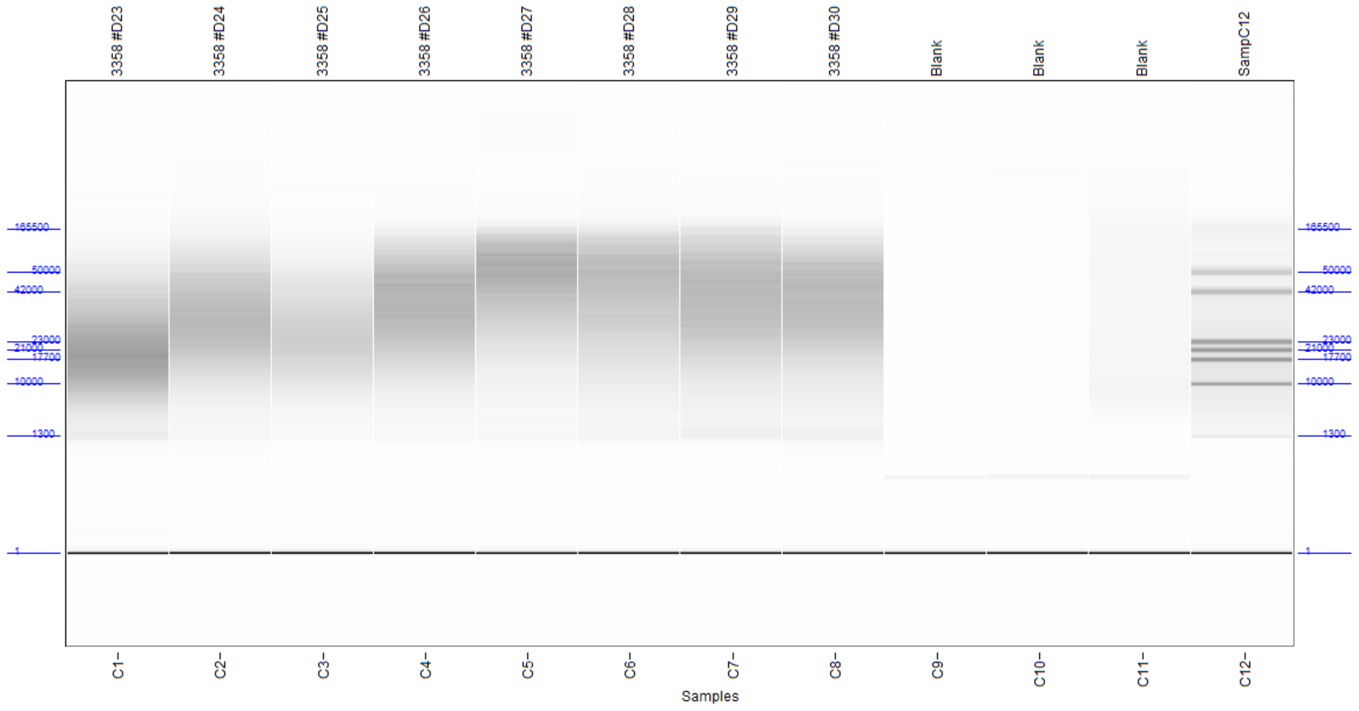

Copyright 2015 Advanced Analytical Technologies, Inc.

GQN Summary

Well	Sample ID	GQN	Size Threshold (bp)	Total Conc. (ng/uL)
C1	3358 #D23	0.4	50000	0.3980
C2	3358 #D24	1.2	50000	0.3212
C3	3358 #D25	0.8	50000	0.2022
C4	3358 #D26	1.8	50000	0.3302
C5	3358 #D27	3.3	50000	0.3020
C6	3358 #D28	2.7	50000	0.3149
C7	3358 #D29	2.2	50000	0.3485
C8	3358 #D30	1.9	50000	0.3373
C9	Blank	4.0	50000	0.0041
C10	Blank	5.3	50000	0.0055
C11	Blank	2.8	50000	0.0677
C12	SampC12			

 Copyright 2015 Advanced Analytical Technologies, Inc.

Filename and Data Path: Z:\project_data\LIMS_projects\ 3358_He_Quan_Sun_10X_Genomics_for_recombination_break_identification_MPIPZ\Public\18-38-12\2017 12 13 18H 38M.raw

C1: 3358 #D23

GQN: 0.4

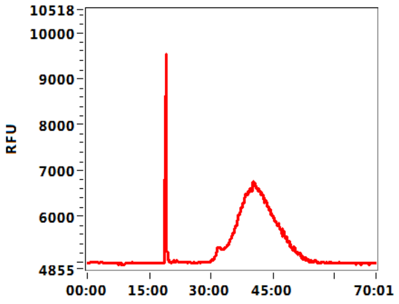

C2: 3358 #D24

GQN: 1.2

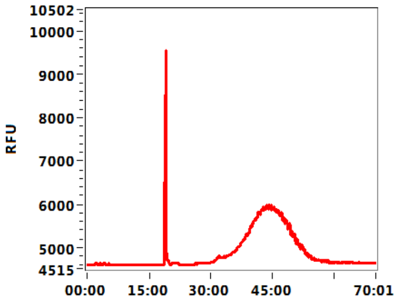

C3: 3358 #D25

GQN: 0.8

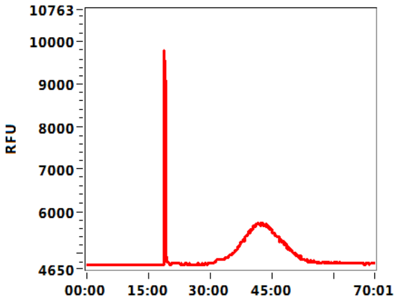

C4: 3358 #D26

GQN: 1.8

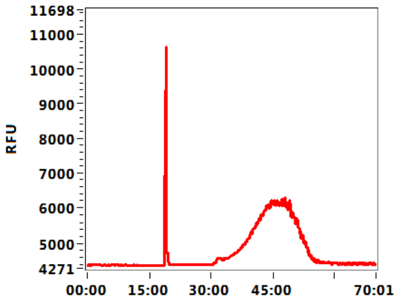

C5: 3358 #D27

GQN: 3.3

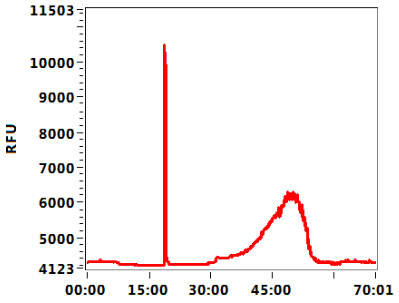

C6: 3358 #D28

GQN: 2.7

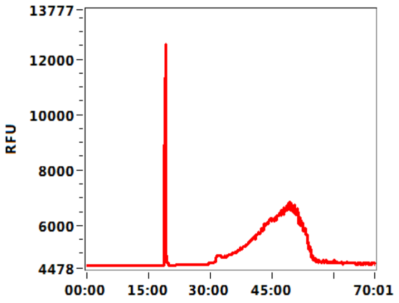

C7: 3358 #D29

GQN: 2.2

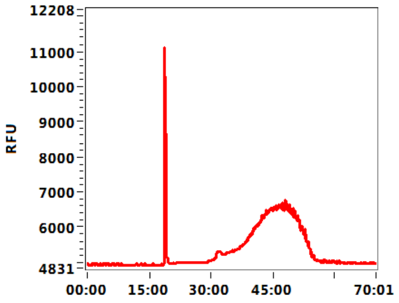

C8: 3358 #D30

GQN: 1.9

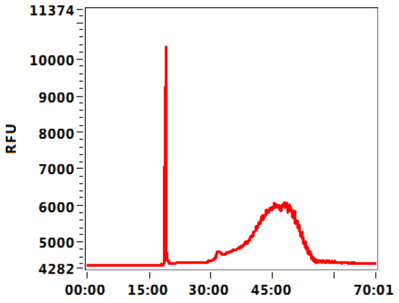

C9: Blank

GQN: 4.0

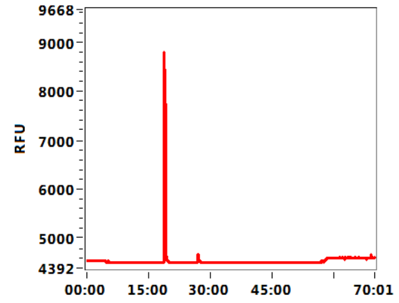

C10: Blank

GQN: 5.3

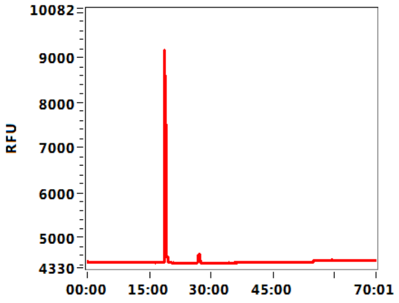

C11: Blank

GQN: 2.8

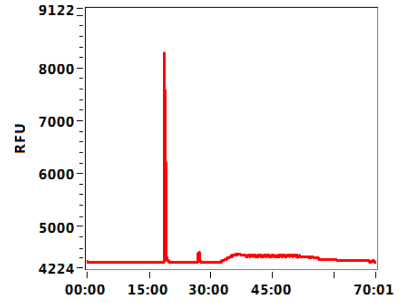

C12: SampC12

GQN: 4.6

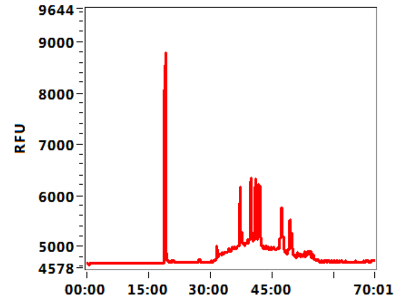

 Copyright 2015 Advanced Analytical Technologies, Inc.

Sample: 3358 #D23
Well Location: C1
Created: 13-Dec-17 6:55 PM

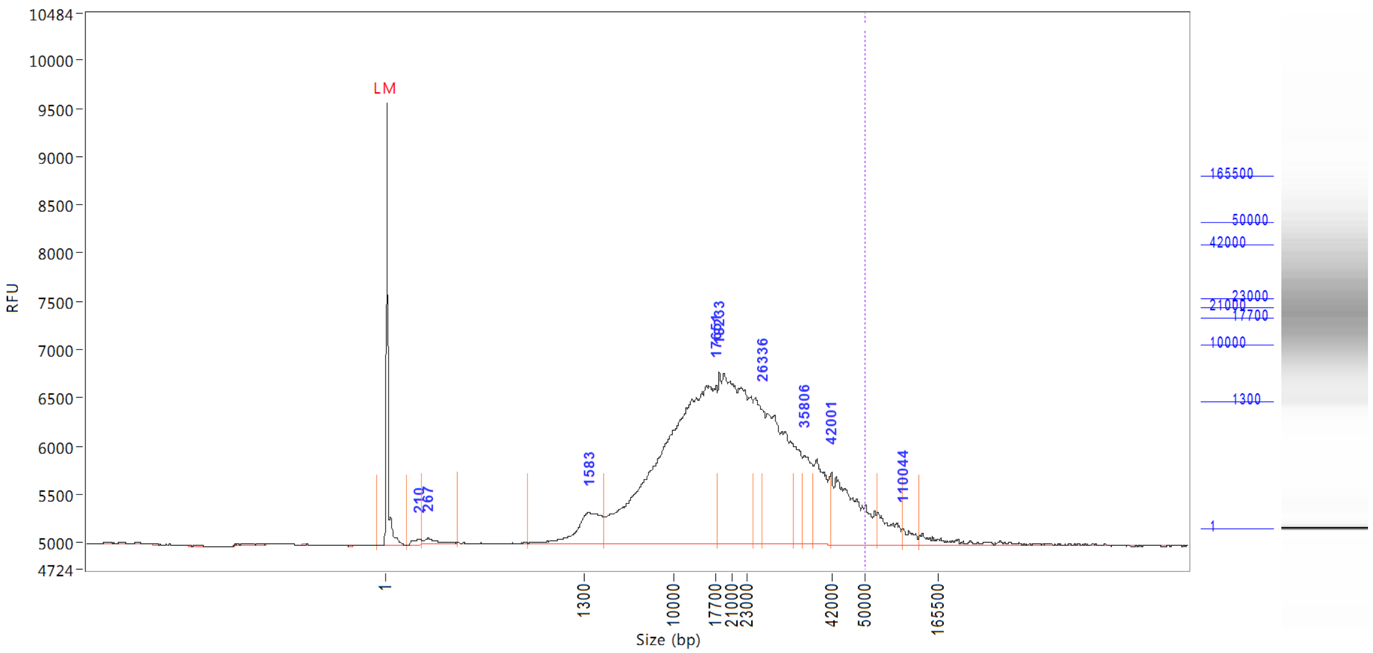

Peak	Size (bp)	Conc. (ng/uL)	From (bp)	To (bp)	Avg. Size (bp)	CV%	RFU	Corr. Peak Area
1	1 (LM)	0.0028	0	136	5	379.74	4596	31.743
2	210	0.0016	136	234	192	12.58	52	1.790
3	267	0.0020	234	468	295	15.14	65	2.315
4	1583	0.0165	923	3123	1786	35.10	319	18.915
5	17651	0.1593	3123	17861	11148	35.39	1646	182.633
6	18233	0.0785	17861	24252	21028	8.38	1777	89.973
7	26336	0.0495	26336	33602	29743	7.04	1403	56.706
8	35806	0.0119	35568	38069	36783	2.00	917	13.611
9	42001	0.0249	42001	69598	48111	13.14	752	28.569
10	110044	0.0020	109210	135479	120999	6.24	160	2.286
11	0	NaN	0	0	>200000	NaN	0	NaN

TIC:	NaN	ng/uL
TIM:	NaN	nmole/L
Total Conc.:	0.3980	ng/uL
GQN:	0.4	
Threshold:	50000	

```
Sample Peak Width (sec): 20   Sample Min Peak Height: 50    Sample Baseline V to V?: Y    Sample Baseline V to V pts: 3
Sample Filter: Binomial       # of Pts for Filter: 3        Sample Start Region (min): 0  Sample End Region (min): 70
Manual Baseline Start (min): 25     Manual Baseline End (min): 70
Marker Peak Width (sec): 8    Marker Min Peak Height: 500   Marker Baseline V to V?: N    Marker Baseline V to V pts: 3
Lower Marker Selection: First Peak > 500 RFU                Upper Marker Selection: Last Peak > 500 RFU
Ladder Size (bp)1, 1300, 10000, 17700, 21000, 23000, 42000, 50000, 165500
Quantification Using: Ladder          Final Concentration (ng/uL): 0.0200          Dilution Factor: 10.0
Min. RFU for Data Processing: 1      Size Threshold (b.p.): 50000
```

 Copyright 2015 Advanced Analytical Technologies, Inc.

Sample: 3358 #D24
Well Location: C2
Created: 13-Dec-17 6:55 PM

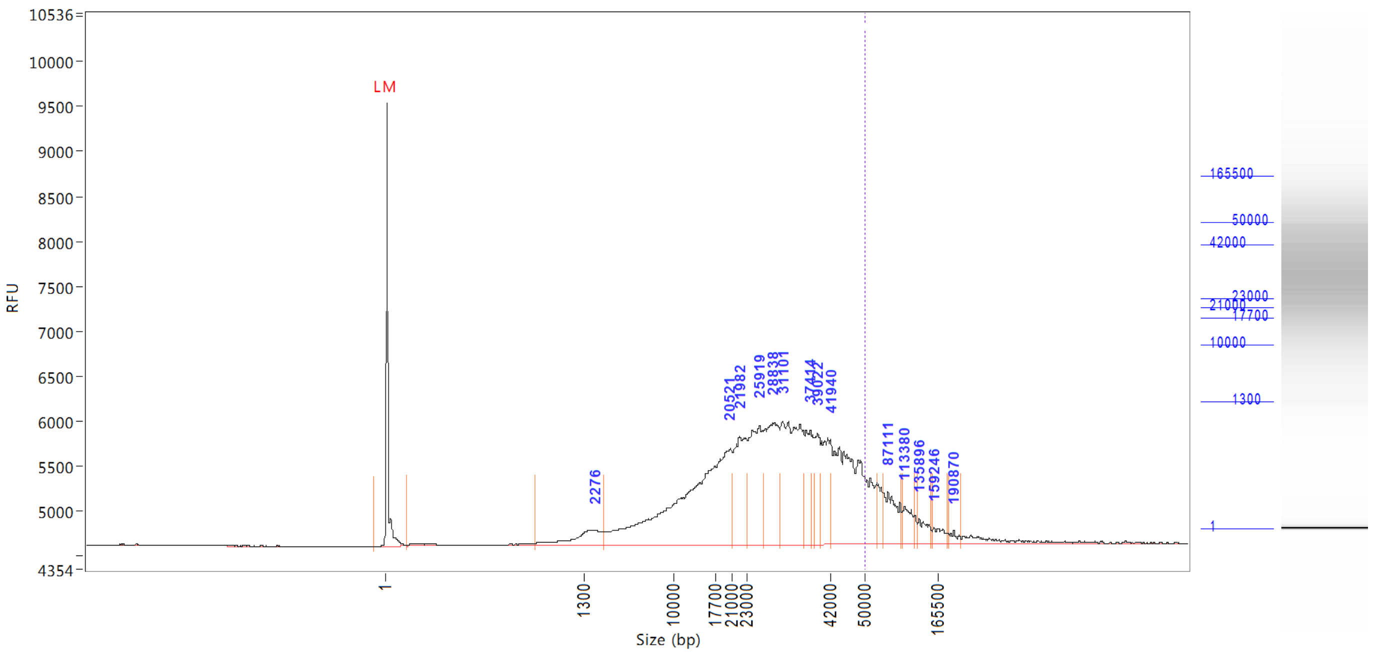

Peak	Size (bp)	Conc. (ng/uL)	From (bp)	To (bp)	Avg. Size (bp)	CV%	RFU	Corr. Peak Area
1	1 (LM)	0.0028	0	133	6	290.59	4939	32.736
2	2276	0.0091	980	3123	1719	36.98	164	10.810
3	20521	0.0861	3123	20947	13243	37.72	1072	101.819
4	21982	0.0231	20947	23180	22059	2.92	1202	27.349
5	25919	0.0252	23180	26694	24953	4.10	1318	29.804
6	28838	0.0271	26694	30446	28572	3.82	1354	32.065
7	31101	0.0391	30446	36104	33228	4.97	1372	46.271
8	37414	0.0110	36104	37771	36932	1.35	1273	13.034
9	39022	0.0090	38248	39677	38955	1.09	1233	10.688
10	41940	0.0461	41940	70849	48805	14.14	1160	54.449
11	87111	0.0086	80439	107542	93156	8.45	586	10.182
12	113380	0.0046	110878	130058	119945	4.68	421	5.467
13	135896	0.0028	134645	154242	143936	4.02	284	3.350
14	159246	0.0022	157578	181304	168755	4.05	191	2.654
15	190870	0.0011	184631	>200000	192900	2.75	154	1.265

TIC:	0.2954	ng/uL
TIM:	0.0291	nmole/L
Total Conc.:	0.3212	ng/uL

GQN:	1.2
Threshold:	50000

Sample Peak Width (sec): 20   Sample Min Peak Height: 50   Sample Baseline V to V?: Y   Sample Baseline V to V pts: 3
Sample Filter: Binomial   # of Pts for Filter: 3   Sample Start Region (min): 0   Sample End Region (min): 70
Manual Baseline Start (min): 25   Manual Baseline End (min): 70
Marker Peak Width (sec): 8   Marker Min Peak Height: 500   Marker Baseline V to V?: N   Marker Baseline V to V pts: 3
Lower Marker Selection: First Peak > 500 RFU   Upper Marker Selection: Last Peak > 500 RFU
Ladder Size (bp)1, 1300, 10000, 17700, 21000, 23000, 42000, 50000, 165500
Quantification Using: Ladder   Final Concentration (ng/uL): 0.0200   Dilution Factor: 10.0
Min. RFU for Data Processing: 1   Size Threshold (b.p.): 50000

 Copyright 2015 Advanced Analytical Technologies, Inc.

Sample: 3358 #D25
Well Location: C3
Created: 13-Dec-17 6:55 PM

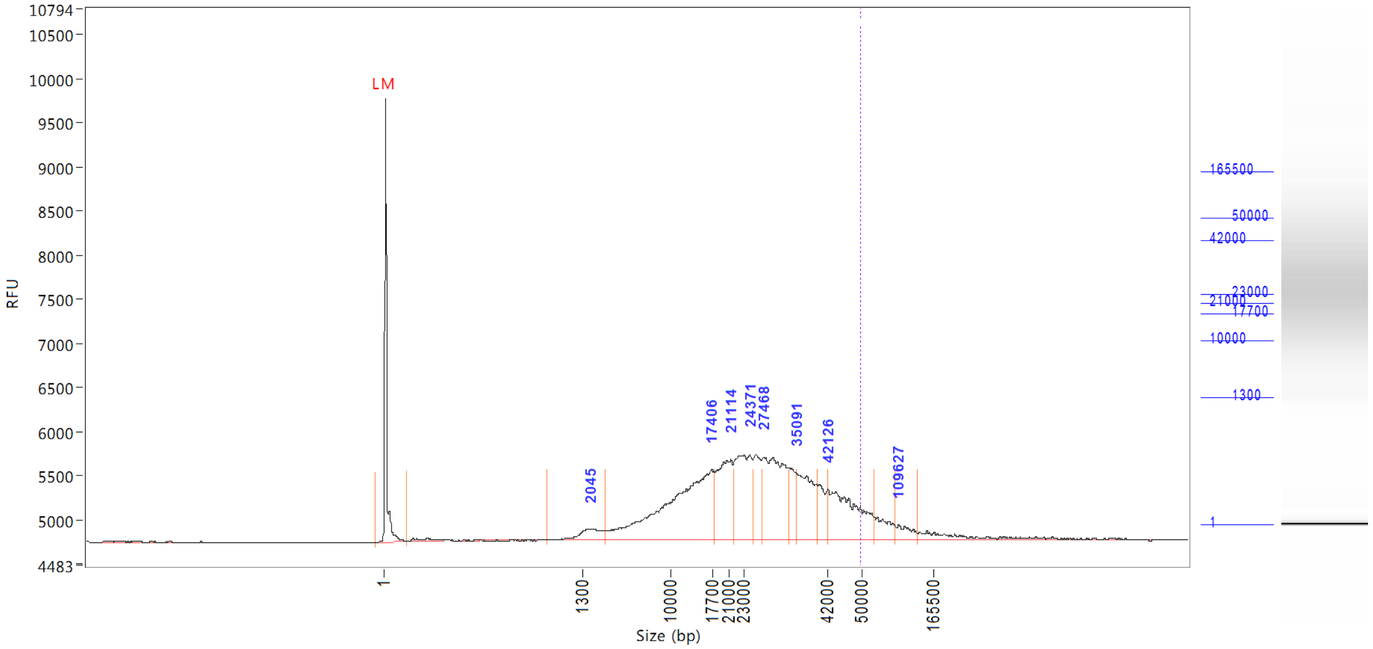

Peak	Size (bp)	Conc. (ng/uL)	From (bp)	To (bp)	Avg. Size (bp)	CV%	RFU	Corr. Peak Area
1	1 (LM)	0.0028	0	146	6	304.02	5040	35.397
2	2045	0.0054	1071	3482	2042	35.76	124	6.962
3	17406	0.0562	3482	17914	11652	33.70	798	71.817
4	21114	0.0199	17914	21491	19838	5.38	918	25.488
5	24371	0.0223	21491	25086	23081	4.48	977	28.461
6	27468	0.0274	27051	33185	30030	5.93	945	35.008
7	35091	0.0161	34913	39737	37232	3.77	751	20.557
8	42126	0.0200	42000	70432	48457	13.69	577	25.586
9	109627	0.0027	104623	139231	120256	8.11	171	3.444

TIC:	0.1700	ng/uL
TIM:	0.0185	nmole/L
Total Conc.:	0.2022	ng/uL
GQN:	0.8	
Threshold:	50000	

```
Sample Peak Width (sec): 20   Sample Min Peak Height: 50    Sample Baseline V to V?: Y    Sample Baseline V to V pts: 3
Sample Filter: Binomial       # of Pts for Filter: 3        Sample Start Region (min): 0  Sample End Region (min): 70
Manual Baseline Start (min): 25     Manual Baseline End (min): 70
Marker Peak Width (sec): 8    Marker Min Peak Height: 500   Marker Baseline V to V?: N    Marker Baseline V to V pts: 3
Lower Marker Selection: First Peak > 500 RFU                Upper Marker Selection: Last Peak > 500 RFU
Ladder Size (bp)1, 1300, 10000, 17700, 21000, 23000, 42000, 50000, 165500
Quantification Using: Ladder          Final Concentration (ng/uL): 0.0200          Dilution Factor: 10.0
Min. RFU for Data Processing: 1      Size Threshold (b.p.): 50000
```

 Copyright 2015 Advanced Analytical Technologies, Inc.

Sample: 3358 #D26
Well Location: C4
Created: 13-Dec-17 6:55 PM

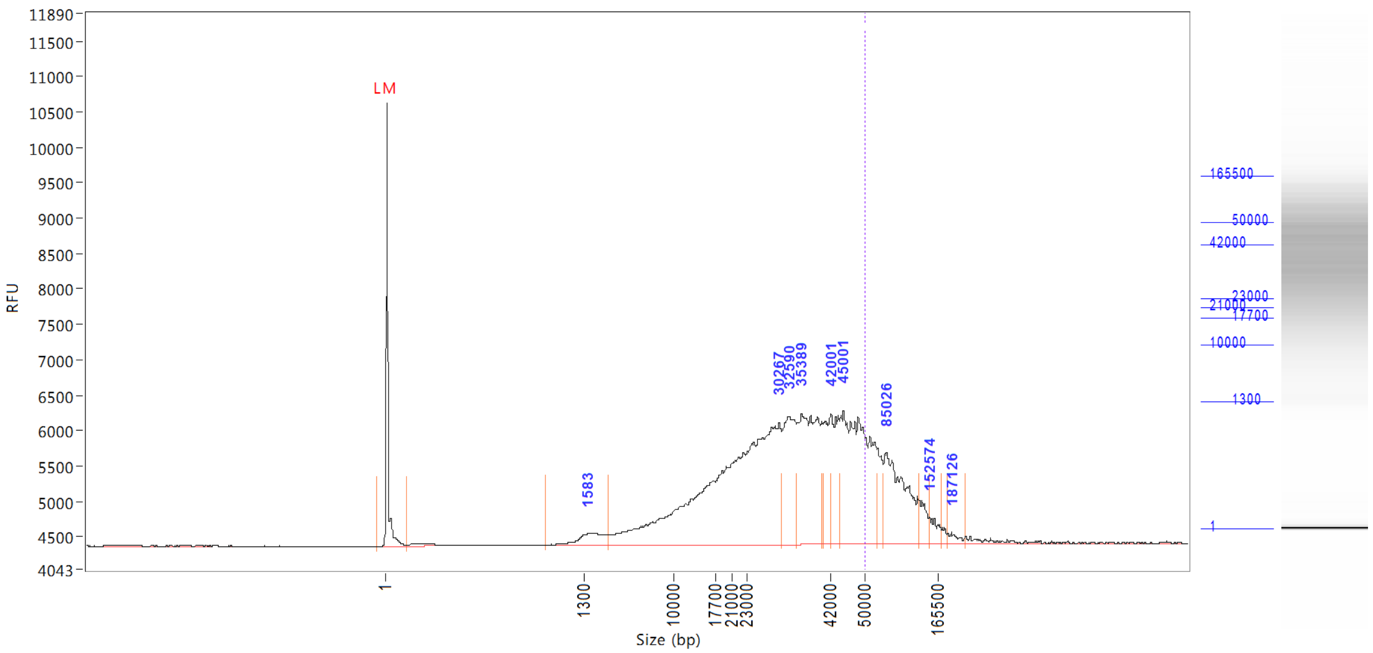

Peak	Size (bp)	Conc. (ng/uL)	From (bp)	To (bp)	Avg. Size (bp)	CV%	RFU	Corr. Peak Area
1	1 (LM)	0.0028	0	134	6	291.57	6273	42.659
2	1583	0.0066	1040	3559	2019	37.75	166	10.173
3	30267	0.1366	3559	30863	19600	37.56	1717	210.503
4	32590	0.0236	30863	34138	32512	2.92	1811	36.292
5	35389	0.0412	34138	40035	37057	4.62	1841	63.459
6	42001	0.0235	40511	44001	42232	2.41	1850	36.136
7	45001	0.0649	42000	70015	49150	14.00	1895	99.903
8	85026	0.0257	80022	135896	104094	15.33	1289	39.617
9	152574	0.0028	152574	172154	161538	3.51	381	4.273
10	187126	0.0014	180472	>200000	193249	4.46	167	2.189

TIC:	0.3262	ng/uL
TIM:	0.0234	nmole/L
Total Conc.:	0.3302	ng/uL
GQN:	1.8	
Threshold:	50000	

```
Sample Peak Width (sec): 20   Sample Min Peak Height: 50    Sample Baseline V to V?: Y    Sample Baseline V to V pts: 3
Sample Filter: Binomial       # of Pts for Filter: 3        Sample Start Region (min): 0  Sample End Region (min): 70
Manual Baseline Start (min): 25     Manual Baseline End (min): 70
Marker Peak Width (sec): 8    Marker Min Peak Height: 500   Marker Baseline V to V?: N    Marker Baseline V to V pts: 3
Lower Marker Selection: First Peak > 500 RFU                Upper Marker Selection: Last Peak > 500 RFU
Ladder Size (bp)1, 1300, 10000, 17700, 21000, 23000, 42000, 50000, 165500
Quantification Using: Ladder          Final Concentration (ng/uL): 0.0200          Dilution Factor: 10.0
Min. RFU for Data Processing: 1      Size Threshold (b.p.): 50000
```

 Copyright 2015 Advanced Analytical Technologies, Inc.

Sample: 3358 #D27
Well Location: C5
Created: 13-Dec-17 6:55 PM

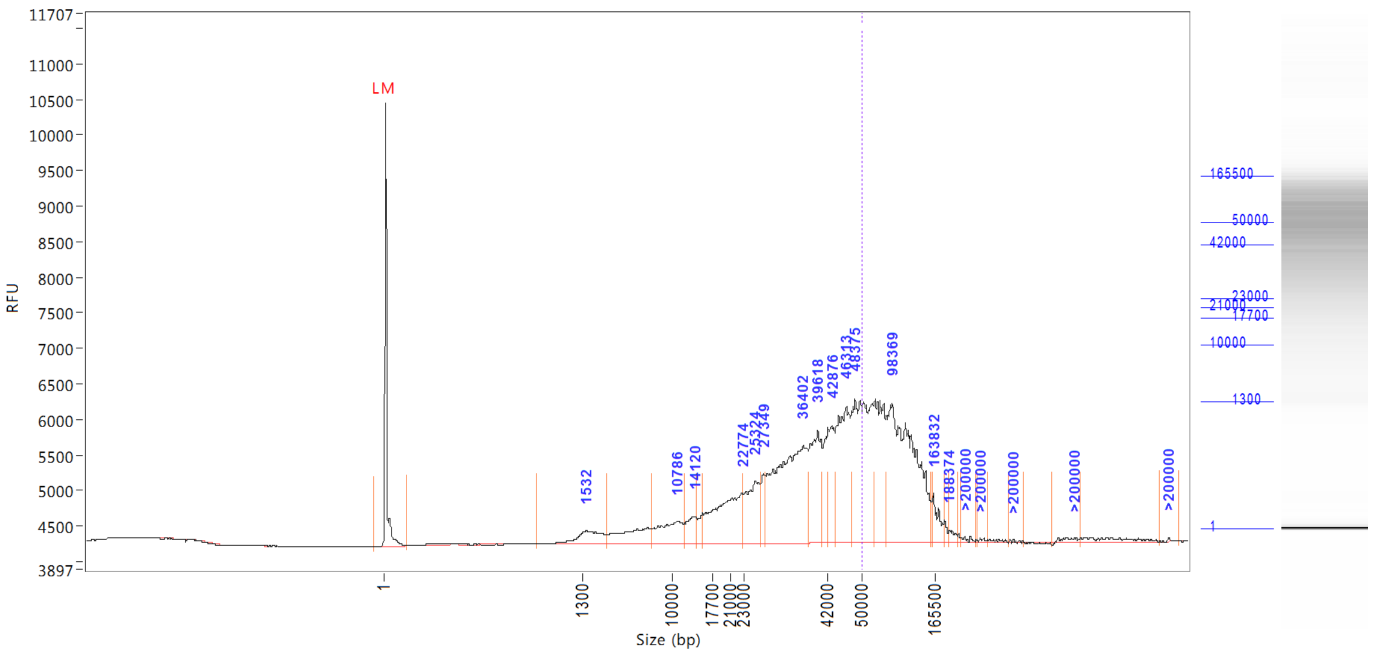

Peak	Size (bp)	Conc. (ng/uL)	From (bp)	To (bp)	Avg. Size (bp)	CV%	RFU	Corr. Peak Area
1	1 (LM)	0.0028	0	141	6	336.26	6242	41.143
2	1532	0.0074	999	3610	1994	38.60	184	10.962
3	10786	0.0101	7998	12355	9938	12.32	313	14.985
4	14120	0.0047	12355	14611	13523	4.82	379	6.924
5	22774	0.0234	15738	22887	19806	10.64	698	34.825
6	25324	0.0135	22887	26694	24809	4.59	870	20.102
7	27349	0.0049	26694	27825	27267	1.24	971	7.231
8	36402	0.0474	27825	37533	32939	8.51	1368	70.426
9	39618	0.0187	37533	40690	39121	2.34	1586	27.714
10	42876	0.0179	40690	43563	42144	1.96	1651	26.541
11	46313	0.0266	43563	47563	45594	2.55	1911	39.556
12	48375	0.0751	41940	70015	49989	14.61	2015	111.511
13	98369	0.0499	88779	159246	118626	16.26	1950	74.139
14	163832	0.0037	162581	181720	170600	3.35	694	5.533
15	188374	0.0010	187542	>200000	194590	2.43	180	1.536
16	>200000	0.0005	>200000	>200000	>200000	3.02	84	0.690
17	>200000	0.0003	>200000	>200000	>200000	1.81	63	0.377
18	>200000	0.0001	>200000	>200000	>200000	2.35	41	0.163
19	>200000	0.0007	>200000	>200000	>200000	2.89	60	0.968
20	>200000	0.0001	>200000	>200000	>200000	1.02	59	0.204

TIC:	0.3058	ng/uL
TIM:	0.0195	nmole/L
Total Conc.:	0.3020	ng/uL

```
Sample Peak Width (sec): 20   Sample Min Peak Height: 50    Sample Baseline V to V?: Y    Sample Baseline V to V pts: 3
Sample Filter: Binomial       # of Pts for Filter: 3        Sample Start Region (min): 0  Sample End Region (min): 70
Manual Baseline Start (min): 25     Manual Baseline End (min): 70
Marker Peak Width (sec): 8    Marker Min Peak Height: 500   Marker Baseline V to V?: N    Marker Baseline V to V pts: 3
Lower Marker Selection: First Peak > 500 RFU                Upper Marker Selection: Last Peak > 500 RFU
Ladder Size (bp)1, 1300, 10000, 17700, 21000, 23000, 42000, 50000, 165500
Quantification Using: Ladder          Final Concentration (ng/uL): 0.0200          Dilution Factor: 10.0
Min. RFU for Data Processing: 1      Size Threshold (b.p.): 50000
```

 Copyright 2015 Advanced Analytical Technologies, Inc.

Sample: 3358 #D27
Well Location: C5
Created: 13-Dec-17 6:55 PM

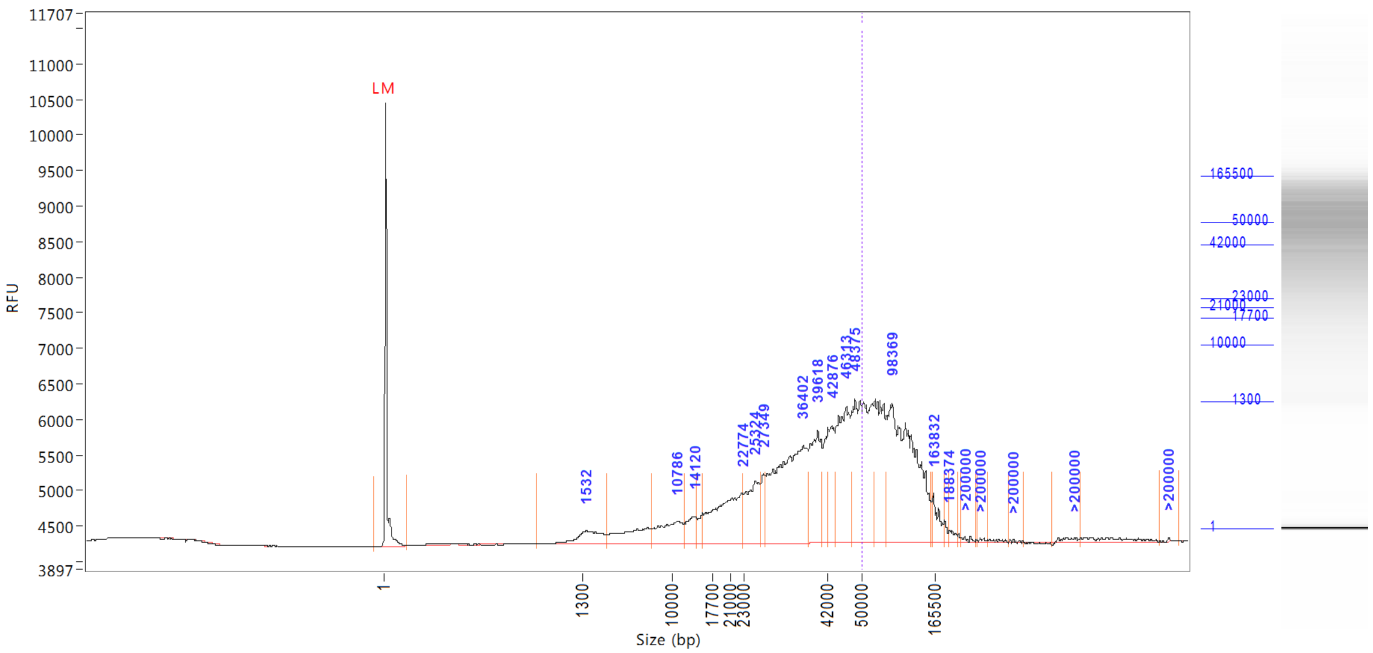

Peak	Size (bp)	Conc. (ng/uL)	From (bp)	To (bp)	Avg. Size (bp)	CV%	RFU	Corr. Peak Area

GQN:	3.3
Threshold:	50000

```
Sample Peak Width (sec): 20   Sample Min Peak Height: 50    Sample Baseline V to V?: Y    Sample Baseline V to V pts: 3
Sample Filter: Binomial       # of Pts for Filter: 3        Sample Start Region (min): 0  Sample End Region (min): 70
Manual Baseline Start (min): 25     Manual Baseline End (min): 70
Marker Peak Width (sec): 8    Marker Min Peak Height: 500   Marker Baseline V to V?: N    Marker Baseline V to V pts: 3
Lower Marker Selection: First Peak > 500 RFU                Upper Marker Selection: Last Peak > 500 RFU
Ladder Size (bp)1, 1300, 10000, 17700, 21000, 23000, 42000, 50000, 165500
Quantification Using: Ladder         Final Concentration (ng/uL): 0.0200          Dilution Factor: 10.0
Min. RFU for Data Processing: 1      Size Threshold (b.p.): 50000
```

 Copyright 2015 Advanced Analytical Technologies, Inc.

Sample: 3358 #D28
Well Location: C6
Created: 13-Dec-17 6:55 PM

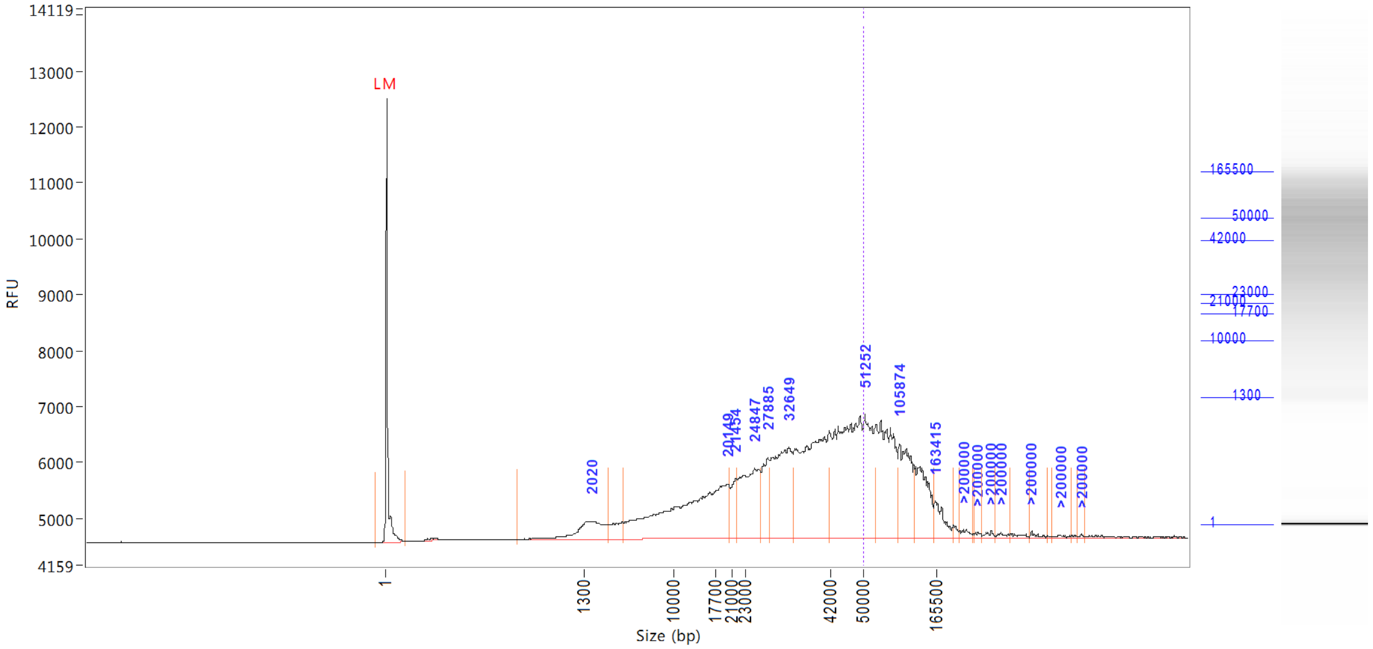

Peak	Size (bp)	Conc. (ng/uL)	From (bp)	To (bp)	Avg. Size (bp)	CV%	RFU	Corr. Peak Area
1	1 (LM)	0.0028	0	129	5	311.53	7958	50.989
2	2020	0.0109	860	3559	1953	39.05	316	20.106
3	20149	0.0566	5047	20628	12896	35.66	980	104.145
4	21454	0.0063	20628	21755	21240	1.52	1073	11.658
5	24847	0.0220	21755	26217	23801	5.62	1262	40.569
6	27885	0.0098	26217	28242	27260	2.19	1451	17.971
7	32649	0.0284	28242	33662	30990	5.08	1627	52.314
8	51252	0.0654	42000	69598	49693	14.18	2211	120.341
9	105874	0.0173	103372	131309	116706	6.91	1695	31.889
10	163415	0.0045	161747	190870	172642	4.60	656	8.215
11	>200000	0.0009	>200000	>200000	>200000	2.67	152	1.637
12	>200000	0.0004	>200000	>200000	>200000	1.48	108	0.710
13	>200000	0.0005	>200000	>200000	>200000	2.12	123	0.996
14	>200000	0.0005	>200000	>200000	>200000	2.57	112	0.978
15	>200000	0.0005	>200000	>200000	>200000	2.67	119	0.831
16	>200000	0.0003	>200000	>200000	>200000	2.20	55	0.486
17	>200000	0.0001	>200000	>200000	>200000	0.78	68	0.272

TIC:	0.2244	ng/uL
TIM:	0.0230	nmole/L
Total Conc.:	0.3149	ng/uL

GQN:	2.7
Threshold:	50000

```
Sample Peak Width (sec): 20   Sample Min Peak Height: 50    Sample Baseline V to V?: Y    Sample Baseline V to V pts: 3
Sample Filter: Binomial       # of Pts for Filter: 3        Sample Start Region (min): 0  Sample End Region (min): 70
Manual Baseline Start (min): 25     Manual Baseline End (min): 70
Marker Peak Width (sec): 8    Marker Min Peak Height: 500   Marker Baseline V to V?: N    Marker Baseline V to V pts: 3
Lower Marker Selection: First Peak > 500 RFU                Upper Marker Selection: Last Peak > 500 RFU
Ladder Size (bp)1, 1300, 10000, 17700, 21000, 23000, 42000, 50000, 165500
Quantification Using: Ladder          Final Concentration (ng/uL): 0.0200          Dilution Factor: 10.0
Min. RFU for Data Processing: 1      Size Threshold (b.p.): 50000
```

 Copyright 2015 Advanced Analytical Technologies, Inc.

Sample: 3358 #D29
Well Location: C7
Created: 13-Dec-17 6:55 PM

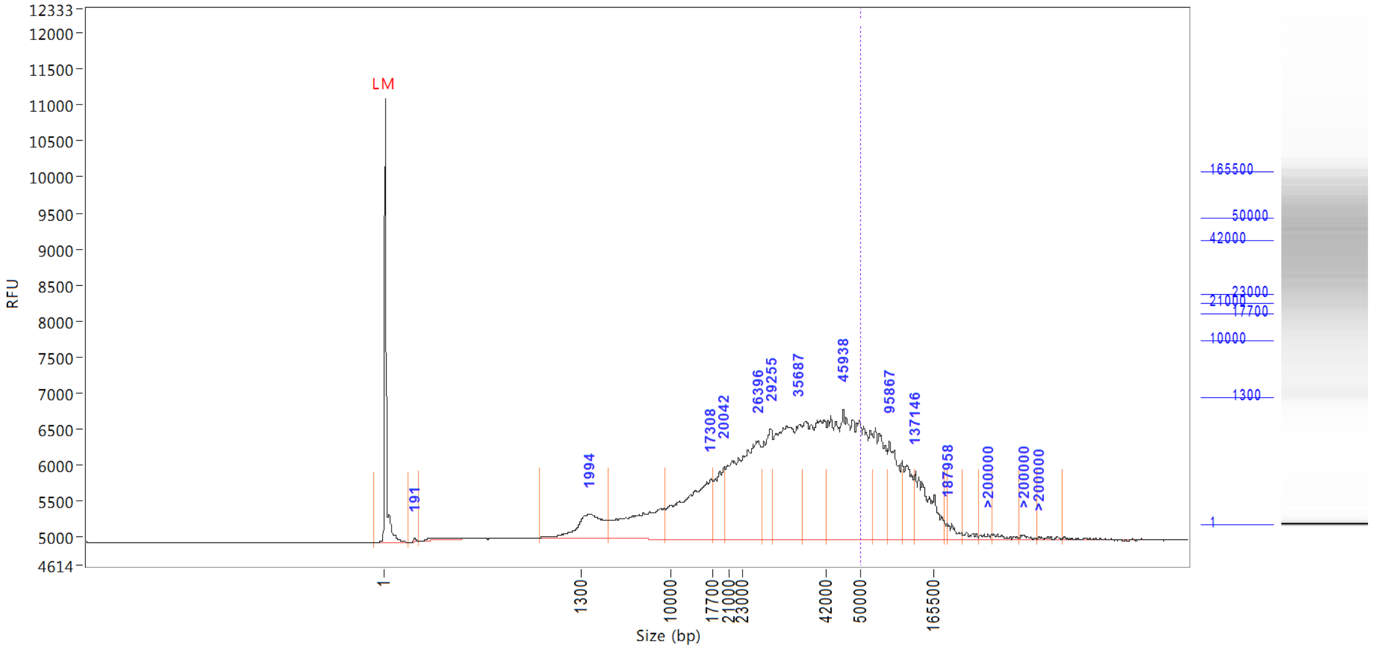

Peak	Size (bp)	Conc. (ng/uL)	From (bp)	To (bp)	Avg. Size (bp)	CV%	RFU	Corr. Peak Area
1	1 (LM)	0.0028	0	153	6	312.92	6169	40.651
2	191	0.0003	153	220	192	3.72	51	0.393
3	1994	0.0151	1016	3867	2100	40.60	340	22.173
4	17308	0.0325	9461	17807	13878	18.15	834	47.746
5	20042	0.0113	17807	20202	19045	3.70	1012	16.612
6	26396	0.0443	20202	27170	23552	8.50	1363	65.022
7	29255	0.0165	27170	29791	28513	2.69	1532	24.197
8	35687	0.0436	29791	36521	33178	5.88	1591	64.045
9	45938	0.0659	41940	70015	49403	14.25	1809	96.701
10	95867	0.0145	93365	117132	104585	6.69	1363	21.352
11	137146	0.0141	135062	182552	154306	8.47	948	20.731
12	187958	0.0017	187542	>200000	197538	3.54	228	2.486
13	>200000	0.0006	>200000	>200000	>200000	2.62	87	0.858
14	>200000	0.0004	>200000	>200000	>200000	2.35	71	0.657
15	>200000	0.0005	>200000	>200000	>200000	3.39	50	0.676

TIC:	0.2614	ng/uL
TIM:	0.0278	nmole/L
Total Conc.:	0.3485	ng/uL

GQN:	2.2
Threshold:	50000

```
Sample Peak Width (sec): 20   Sample Min Peak Height: 50    Sample Baseline V to V?: Y    Sample Baseline V to V pts: 3
Sample Filter: Binomial       # of Pts for Filter: 3        Sample Start Region (min): 0  Sample End Region (min): 70
Manual Baseline Start (min): 25     Manual Baseline End (min): 70
Marker Peak Width (sec): 8    Marker Min Peak Height: 500   Marker Baseline V to V?: N    Marker Baseline V to V pts: 3
Lower Marker Selection: First Peak > 500 RFU                Upper Marker Selection: Last Peak > 500 RFU
Ladder Size (bp)1, 1300, 10000, 17700, 21000, 23000, 42000, 50000, 165500
Quantification Using: Ladder          Final Concentration (ng/uL): 0.0200         Dilution Factor: 10.0
Min. RFU for Data Processing: 1      Size Threshold (b.p.): 50000
```

 Copyright 2015 Advanced Analytical Technologies, Inc.

Sample: 3358 #D30
Well Location: C8
Created: 13-Dec-17 6:55 PM

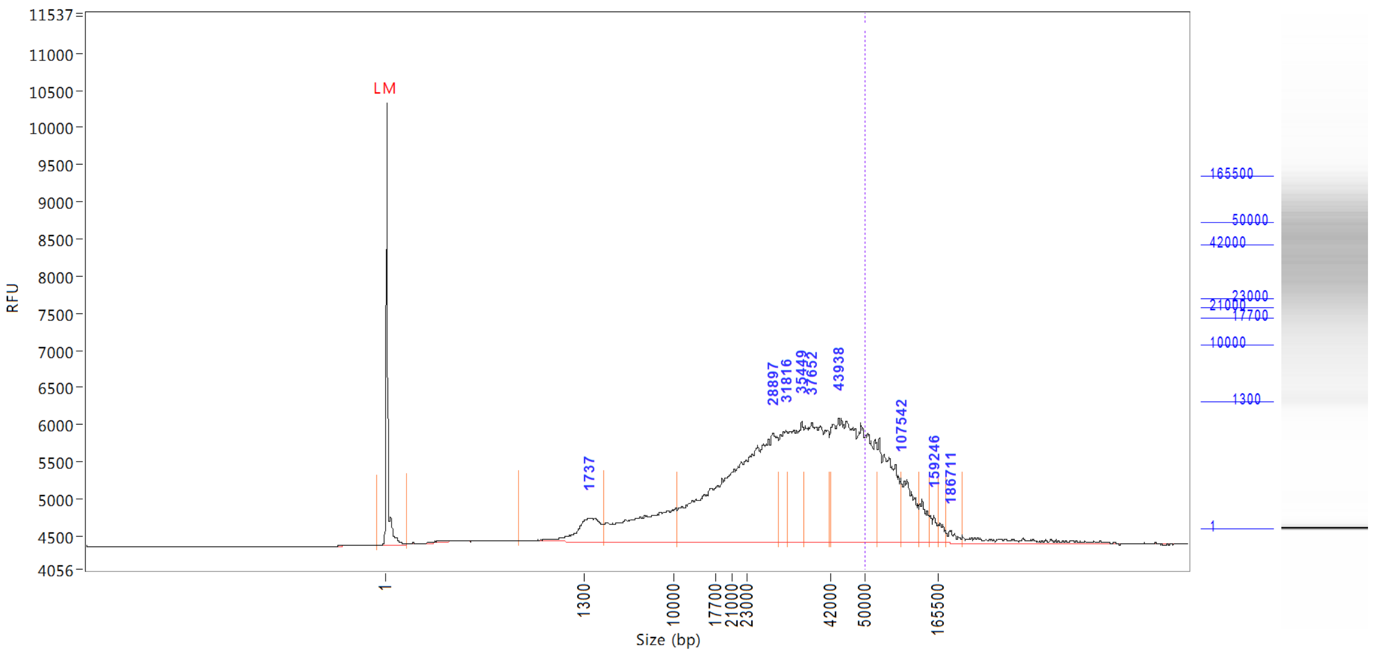

Peak	Size (bp)	Conc. (ng/uL)	From (bp)	To (bp)	Avg. Size (bp)	CV%	RFU	Corr. Peak Area
1	1 (LM)	0.0028	0	140	5	341.08	5959	38.526
2	1737	0.0136	870	3123	1733	35.74	319	18.882
3	28897	0.1015	10442	30148	21637	24.77	1472	141.206
4	31816	0.0150	30148	32352	31260	2.08	1507	20.816
5	35449	0.0245	32352	35925	34150	3.08	1634	34.042
6	37652	0.0387	35925	41702	38770	4.32	1618	53.849
7	43938	0.0663	41940	70015	49214	14.10	1666	92.281
8	107542	0.0110	107542	136313	120495	6.80	851	15.327
9	159246	0.0022	152574	165915	158840	2.46	377	3.117
10	186711	0.0013	178808	>200000	189691	3.72	163	1.848

TIC:	0.2741	ng/uL
TIM:	0.0266	nmole/L
Total Conc.:	0.3373	ng/uL
GQN:	1.9	
Threshold:	50000	

```
Sample Peak Width (sec): 20   Sample Min Peak Height: 50    Sample Baseline V to V?: Y    Sample Baseline V to V pts: 3
Sample Filter: Binomial       # of Pts for Filter: 3        Sample Start Region (min): 0  Sample End Region (min): 70
Manual Baseline Start (min): 25     Manual Baseline End (min): 70
Marker Peak Width (sec): 8    Marker Min Peak Height: 500   Marker Baseline V to V?: N    Marker Baseline V to V pts: 3
Lower Marker Selection: First Peak > 500 RFU                Upper Marker Selection: Last Peak > 500 RFU
Ladder Size (bp)1, 1300, 10000, 17700, 21000, 23000, 42000, 50000, 165500
Quantification Using: Ladder          Final Concentration (ng/uL): 0.0200          Dilution Factor: 10.0
Min. RFU for Data Processing: 1      Size Threshold (b.p.): 50000
```


Copyright 2015 Advanced Analytical Technologies, Inc.

Sample: Blank
Well Location: C9
Created: 13-Dec-17 6:55 PM

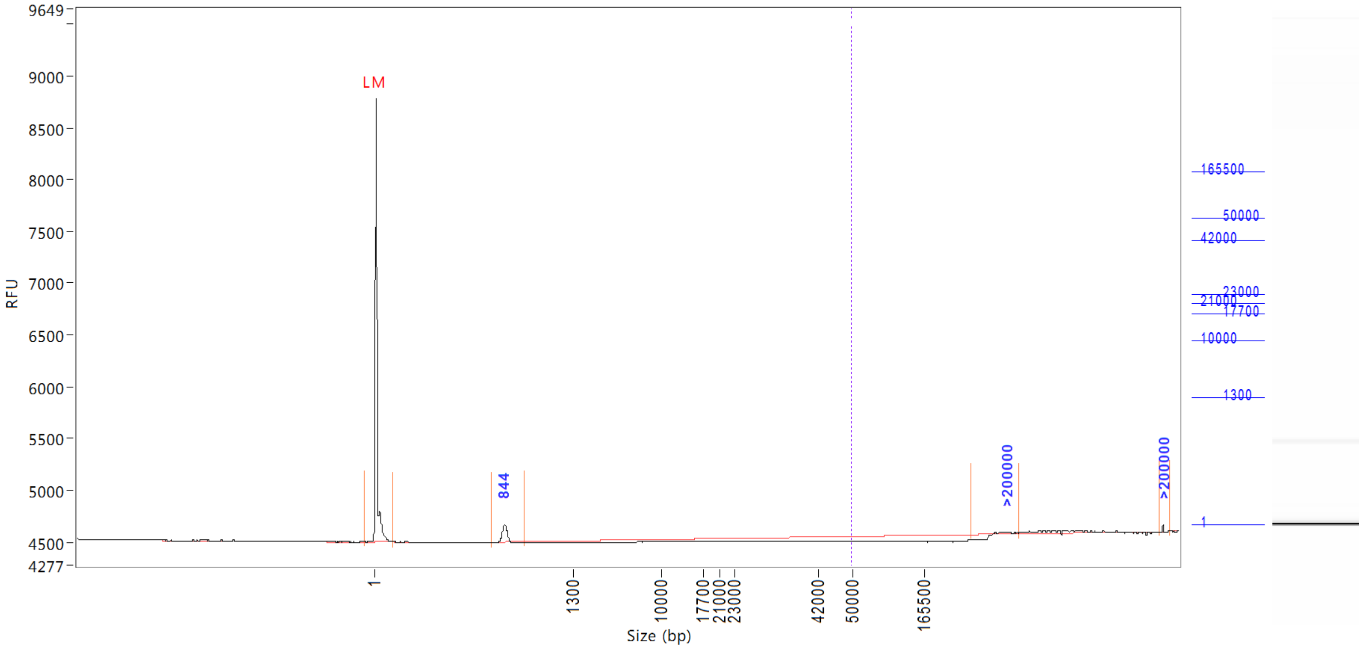

Peak	Size (bp)	Conc. (ng/uL)	From (bp)	To (bp)	Avg. Size (bp)	CV%	RFU	Corr. Peak Area
1	1 (LM)	0.0028	0	115	3	409.22	4287	28.632
2	844	0.0021	765	977	843	1.65	167	2.172
3	>200000	0.0003	>200000	>200000	>200000	3.76	18	0.298
4	>200000	0.0001	>200000	>200000	>200000	0.22	66	0.065

TIC:	0.0025	ng/uL
TIM:	0.0041	nmole/L
Total Conc.:	0.0041	ng/uL
GQN:	4.0	
Threshold:	50000	

Sample Peak Width (sec): 20 Sample Min Peak Height: 50 Sample Baseline V to V?: Y Sample Baseline V to V pts: 3
Sample Filter: Binomial # of Pts for Filter: 3 Sample Start Region (min): 0 Sample End Region (min): 70
Manual Baseline Start (min): 25 Manual Baseline End (min): 70
Marker Peak Width (sec): 8 Marker Min Peak Height: 500 Marker Baseline V to V?: N Marker Baseline V to V pts: 3
Lower Marker Selection: First Peak > 500 RFU Upper Marker Selection: Last Peak > 500 RFU
Ladder Size (bp)1, 1300, 10000, 17700, 21000, 23000, 42000, 50000, 165500
Quantification Using: Ladder Final Concentration (ng/uL): 0.0200 Dilution Factor: 10.0
Min. RFU for Data Processing: 1 Size Threshold (b.p.): 50000

 Copyright 2015 Advanced Analytical Technologies, Inc.

Sample: Blank
Well Location: C10
Created: 13-Dec-17 6:55 PM

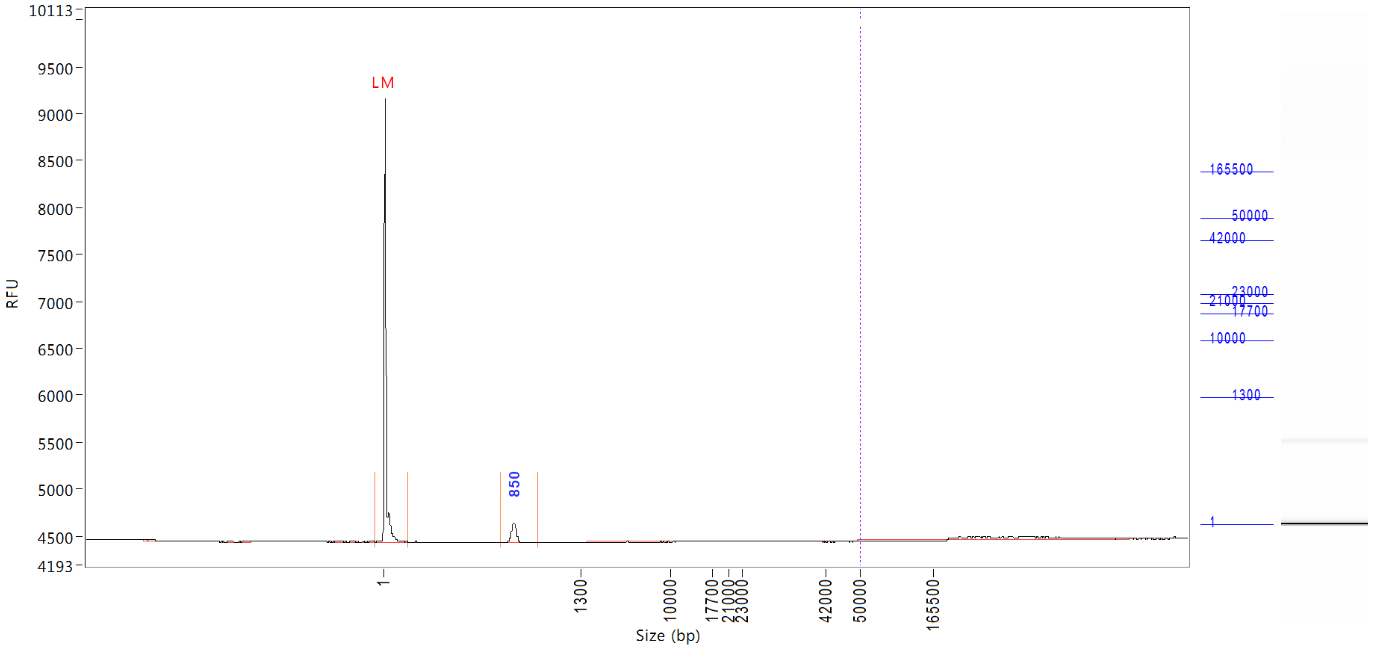

Peak	Size (bp)	Conc. (ng/uL)	From (bp)	To (bp)	Avg. Size (bp)	CV%	RFU	Corr. Peak Area
1	1 (LM)	0.0028	0	160	5	337.21	4727	31.423
2	850	0.0024	769	1015	847	1.74	204	2.749

TIC:	0.0024	ng/uL
TIM:	0.0047	nmole/L
Total Conc.:	0.0055	ng/uL

GQN:	5.3
Threshold:	50000

Sample Peak Width (sec): 20 Sample Min Peak Height: 50 Sample Baseline V to V?: Y Sample Baseline V to V pts: 3
Sample Filter: Binomial # of Pts for Filter: 3 Sample Start Region (min): 0 Sample End Region (min): 70
Manual Baseline Start (min): 25 Manual Baseline End (min): 70
Marker Peak Width (sec): 8 Marker Min Peak Height: 500 Marker Baseline V to V?: N Marker Baseline V to V pts: 3
Lower Marker Selection: First Peak > 500 RFU Upper Marker Selection: Last Peak > 500 RFU
Ladder Size (bp)1, 1300, 10000, 17700, 21000, 23000, 42000, 50000, 165500
Quantification Using: Ladder Final Concentration (ng/uL): 0.0200 Dilution Factor: 10.0
Min. RFU for Data Processing: 1 Size Threshold (b.p.): 50000

 Copyright 2015 Advanced Analytical Technologies, Inc.

Sample: Blank
Well Location: C11
Created: 13-Dec-17 6:55 PM

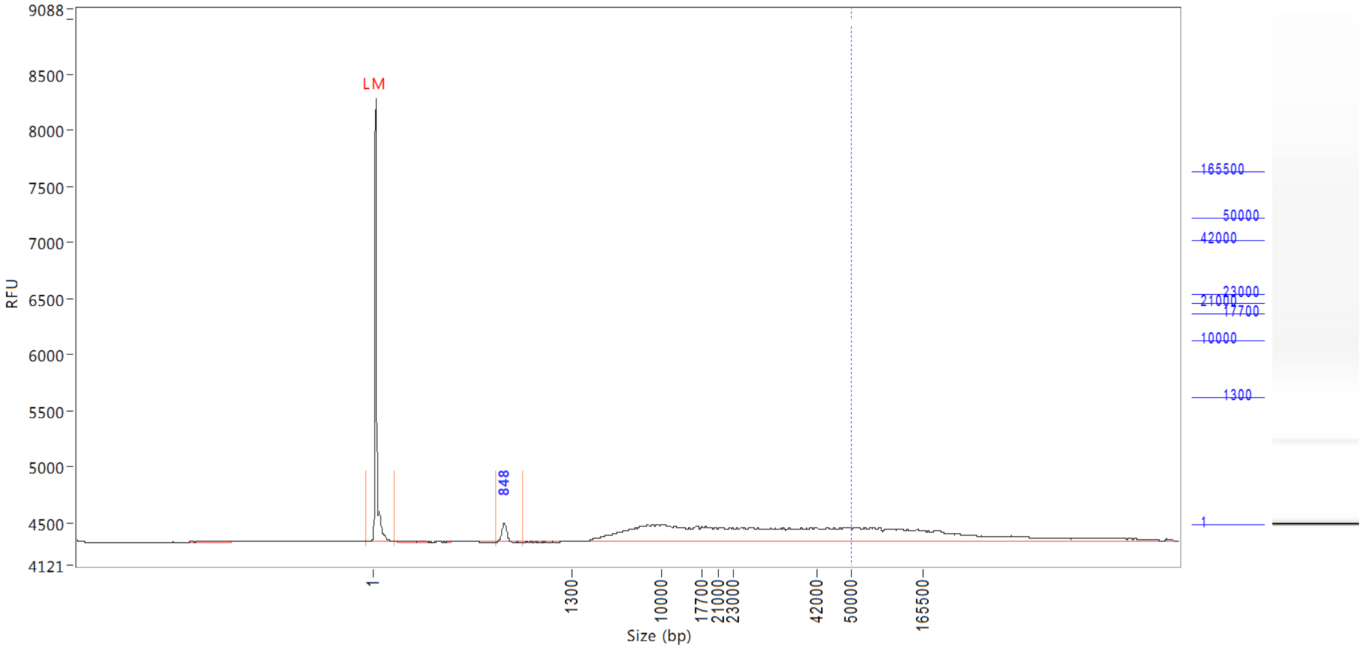

Peak	Size (bp)	Conc. (ng/uL)	From (bp)	To (bp)	Avg. Size (bp)	CV%	RFU	Corr. Peak Area
1	1 (LM)	0.0028	0	133	4	342.64	3960	26.148
2	848	0.0023	793	972	846	1.63	168	2.197

TIC:	0.0023	ng/uL
TIM:	0.0045	nmole/L
Total Conc.:	0.0677	ng/uL
GQN:	2.8	
Threshold:	50000	

Sample Peak Width (sec): 20 Sample Min Peak Height: 50 Sample Baseline V to V?: Y Sample Baseline V to V pts: 3
Sample Filter: Binomial # of Pts for Filter: 3 Sample Start Region (min): 0 Sample End Region (min): 70
Manual Baseline Start (min): 25 Manual Baseline End (min): 70
Marker Peak Width (sec): 8 Marker Min Peak Height: 500 Marker Baseline V to V?: N Marker Baseline V to V pts: 3
Lower Marker Selection: First Peak > 500 RFU Upper Marker Selection: Last Peak > 500 RFU
Ladder Size (bp)1, 1300, 10000, 17700, 21000, 23000, 42000, 50000, 165500
Quantification Using: Ladder Final Concentration (ng/uL): 0.0200 Dilution Factor: 10.0
Min. RFU for Data Processing: 1 Size Threshold (b.p.): 50000

 Copyright 2015 Advanced Analytical Technologies, Inc.

Sample: SampC12
Well Location: C12
Created: 13-Dec-17 6:55 PM

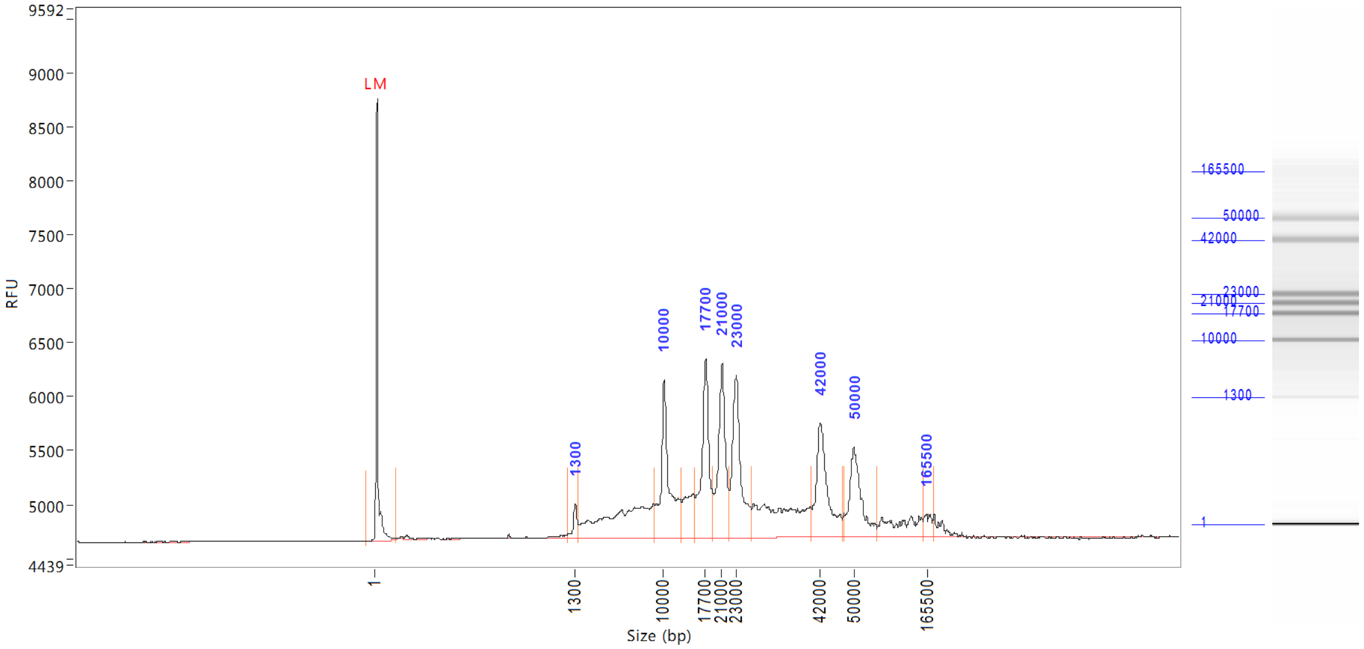

Peak	Size (bp)	Conc. (ng/uL)	From (bp)	To (bp)	Avg. Size (bp)	CV%	RFU	Corr. Peak Area
1	1 (LM)	0.0028	0	131	5	351.85	4103	27.266
2	1300	0.0028	1250	1558	1334	5.76	323	2.802
3	10000	0.0247	9076	13238	10617	10.38	1466	24.293
4	17700	0.0231	15738	19244	17551	4.52	1650	22.699
5	21000	0.0216	19244	22019	20808	3.09	1609	21.241
6	23000	0.0232	22019	26336	23398	4.46	1503	22.819
7	42000	0.0187	40154	47188	42793	3.83	1057	18.386
8	50000	0.0147	47625	86277	56043	16.63	835	14.518
9	165500	0.0023	160079	176729	168118	2.90	213	2.259

TIC:	0.1310	ng/uL
TIM:	0.0140	nmole/L
Total Conc.:	0.2000	ng/uL

```
Sample Peak Width (sec): 20   Sample Min Peak Height: 300   Sample Baseline V to V?: Y    Sample Baseline V to V pts: 3
Sample Filter: Binomial       # of Pts for Filter: 3        Sample Start Region (min): 0  Sample End Region (min): 70
Manual Baseline Start (min): 25     Manual Baseline End (min): 70
Marker Peak Width (sec): 8    Marker Min Peak Height: 500   Marker Baseline V to V?: N    Marker Baseline V to V pts: 3
Lower Marker Selection: First Peak > 500 RFU                Upper Marker Selection: Last Peak > 500 RFU
Ladder Size (bp)1, 1300, 10000, 17700, 21000, 23000, 42000, 50000, 165500
Quantification Using: Ladder          Final Concentration (ng/uL): 0.0200          Dilution Factor: 10.0
Min. RFU for Data Processing: 1      Size Threshold (b.p.): 50000
```

 Copyright 2015 Advanced Analytical Technologies, Inc.

Sample: SampC12
Well Location: C12
Created: 13-Dec-17 6:55 PM
Fit Type: Point to Point

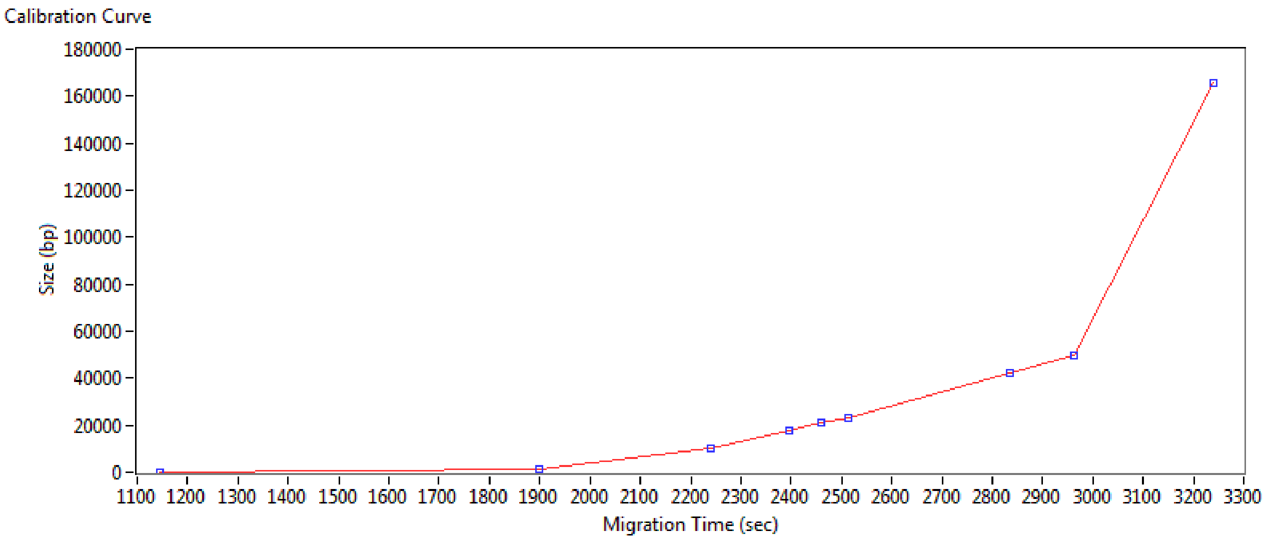

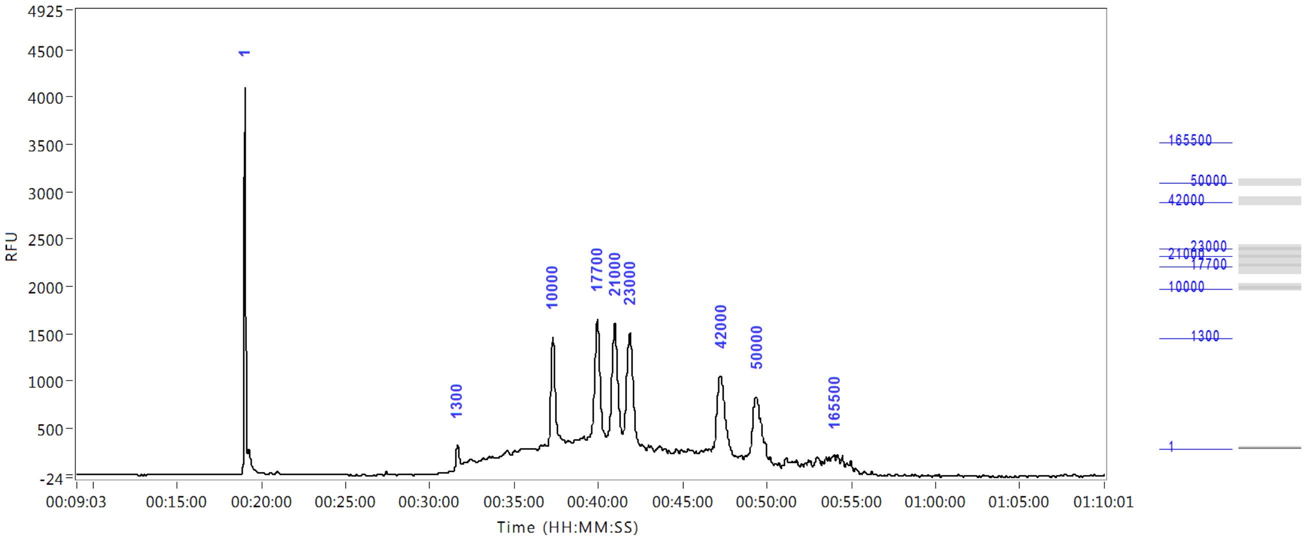

 Copyright 2015 Advanced Analytical Technologies, Inc.
