# Supplementary material for: Linked-read sequencing of gametes allows efficient genome-wide analysis of meiotic recombination
Source: Nat Commun. 2019 Sep 20;10:4310. doi: 10.1038/s41467-019-12209-2 (PMC6754367; doi:10.1038/s41467-019-12209-2)
Supplement: Supplementary file 12 — Source Data [file 41467_2019_12209_MOESM12_ESM.zip › Source_Data/Source_Data_supplementary_Figure_4.zip!/Source_Data_supplementary_Figure_4/supplemenary_Figure_4_3358_FEMTO_B1-B4_summary.pdf]

FEMTO Pulse Run Summary:

Filename and Data Path: Z:\project_data\LIMS_projects\
Filename and Data Path: 3358_He_Quan_Sun_10X_Genomics_for_recombination_break_identification_MPIPZ\
Filename and Data Path: Lab\14-12-06\2017 12 04 14H 12M.raw
Created: 04-Dec-17 2:28 PM
# of Capillaries: 4
Array Serial #: 041017-03USFP
Effect Length: 22 cm
Array Usage Count: 55
FEMTO Pulse Version #: 1.0.0.32
Device Serial #: F12119

METHOD INFORMATION
Method Name: FP-1002-22 - gDNA 165Kb.mthds
Gel Prime: No
Full Conditioning: Yes
Gel Prime to Buffer: No
Gel Selection: Gel 1
Perform Prerun: -5,0 kV, 60 sec.
Rinse: No
Marker 1: No
Rinse: Tray: 3, Row: A, # Dips: 1
Sample Injection: -5,0 kV, 30 sec.
Separation: 70,0 min.
Tray Name: Tray-1

Analysis Mode: gDNA

NOTES

Time Shift: Using configured: slope=0.00025, intercept=0.0

 Copyright 2015 Advanced Analytical Technologies, Inc.

Gel Image

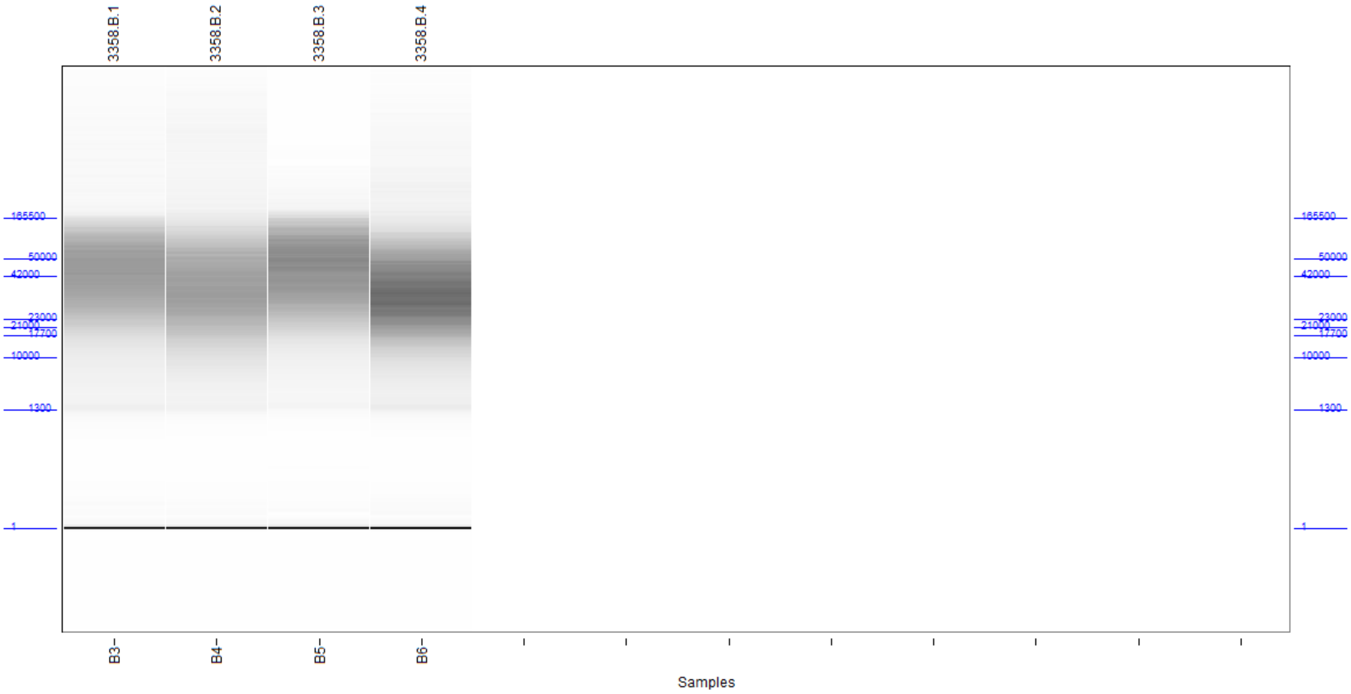

Copyright 2015 Advanced Analytical Technologies, Inc.

GQN Summary

Well	Sample ID	GQN	Size Threshold (bp)	Total Conc. (ng/uL)
B3	3358.B.1	2,9	50000	0,2103
B4	3358.B.2	2,4	50000	0,2285
B5	3358.B.3	3,2	50000	0,2148
B6	3358.B.4	1,8	50000	0,2936

 Copyright 2015 Advanced Analytical Technologies, Inc.

Filename and Data Path: Z:\project_data\LIMS_projects\
3358_He_Quan_Sun_10X_Genomics_for_recombination_break_identification_MPIPZ\Lab\14-12-06\
2017 12 04 14H 12M.raw

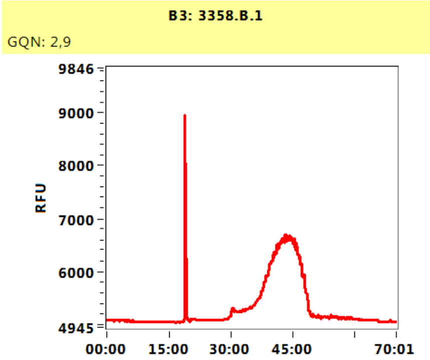

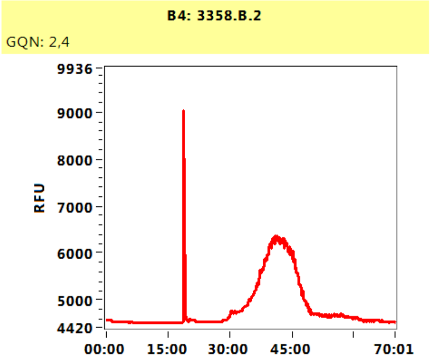

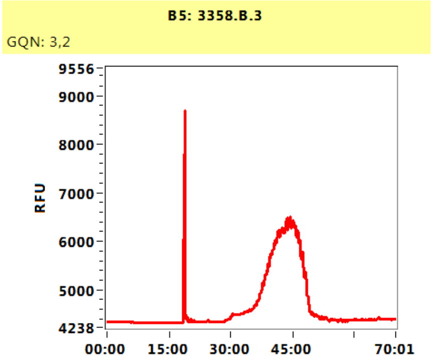

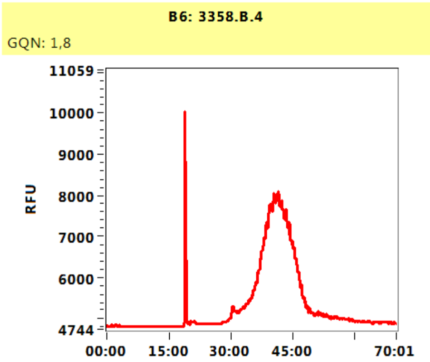

 Copyright 2015 Advanced Analytical Technologies, Inc.

Sample: 3358.B.1
Well Location: B3
Created: 04-Dec-17 2:28 PM

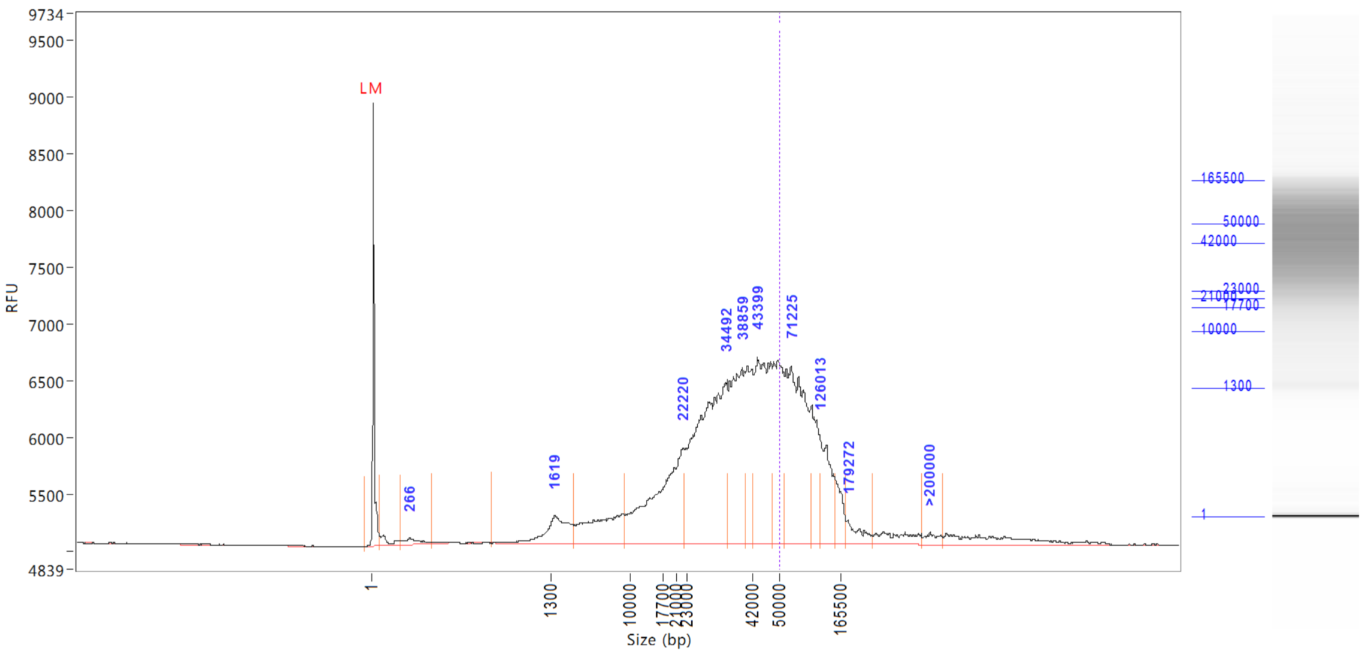

Peak	Size (bp)	Conc. (ng/uL)	From (bp)	To (bp)	Avg. Size (bp)	CV%	RFU	Corr. Peak Area
1	1 (LM)	0,0013	0	55	4	316,06	3907	23,870
2	266	0,0015	202	428	290	19,78	57	2,813
3	1619	0,0070	867	3729	2018	40,43	243	13,123
4	22220	0,0254	9422	22464	17073	23,04	844	47,895
5	34492	0,0420	22464	34799	28933	12,46	1442	79,125
6	38859	0,0210	34799	40008	37432	4,06	1553	39,511
7	43399	0,0237	42156	47903	45020	3,72	1640	44,709
8	71225	0,0274	58392	109231	82458	17,79	1567	51,603
9	126013	0,0079	126013	153160	138525	5,65	943	14,853
10	179272	0,0022	174845	>200000	195846	7,45	210	4,165
11	>200000	0,0010	>200000	>200000	>200000	3,33	113	1,837

TIC:	0,1591	ng/uL
TIM:	0,0215	nmole/L
Total Conc.:	0,2103	ng/uL
GQN:	2,9	
Threshold:	50000	

```
Sample Peak Width (sec): 20   Sample Min Peak Height: 50    Sample Baseline V to V?: Y    Sample Baseline V to V pts: 3
Sample Filter: Binomial       # of Pts for Filter: 3        Sample Start Region (min): 0  Sample End Region (min): 70
Manual Baseline Start (min): 25     Manual Baseline End (min): 70
Marker Peak Width (sec): 8    Marker Min Peak Height: 500   Marker Baseline V to V?: N    Marker Baseline V to V pts: 3
Lower Marker Selection: First Peak > 500 RFU                Upper Marker Selection: Last Peak > 500 RFU
Ladder Size (bp)1, 1300, 10000, 17700, 21000, 23000, 42000, 50000, 165500
Quantification Using: Ladder          Final Concentration (ng/uL): 0,0200          Dilution Factor: 10,0
Min. RFU for Data Processing: 1      Size Threshold (b.p.): 50000
```

 Copyright 2015 Advanced Analytical Technologies, Inc.

Sample: 3358.B.2
Well Location: B4
Created: 04-Dec-17 2:28 PM

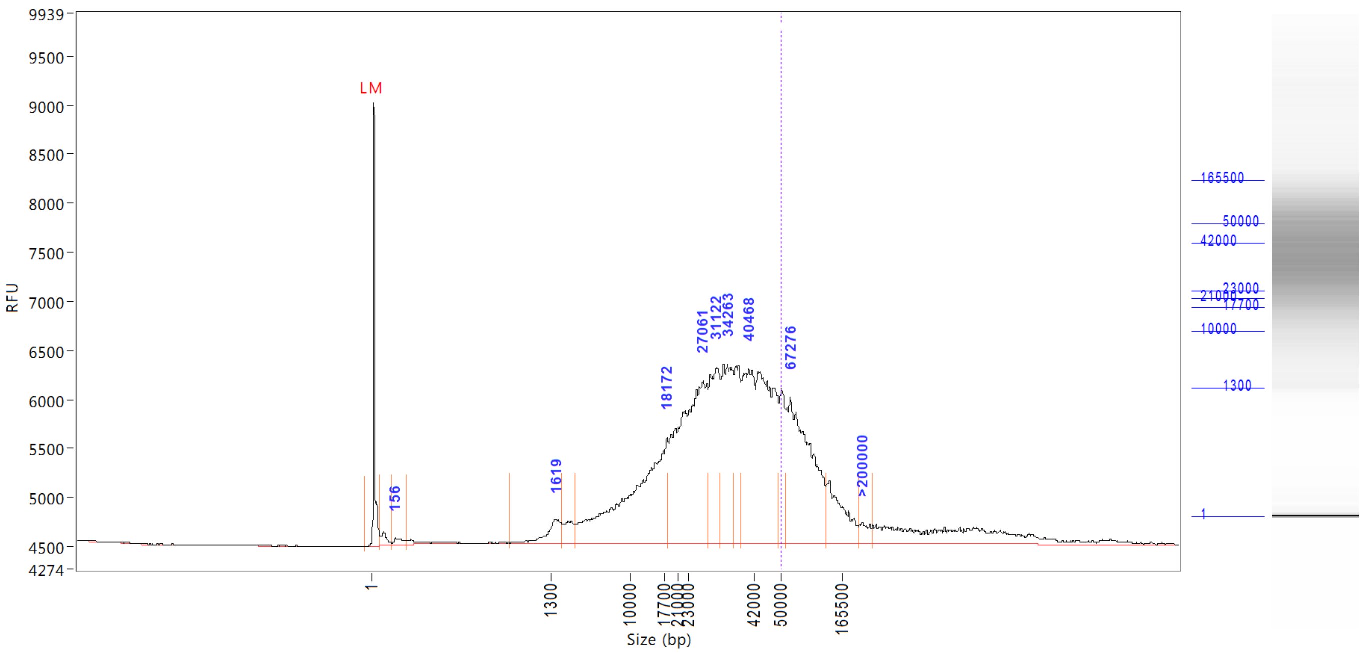

Peak	Size (bp)	Conc. (ng/uL)	From (bp)	To (bp)	Avg. Size (bp)	CV%	RFU	Corr. Peak Area
1	1 (LM)	0,0013	0	51	4	316,36	4524	27,282
2	156	0,0011	133	241	185	15,31	70	2,370
3	1619	0,0041	992	2428	1558	25,48	238	8,780
4	18172	0,0381	3844	18644	11712	36,47	1083	81,960
5	27061	0,0411	18644	28670	23697	11,92	1653	88,475
6	31122	0,0161	28670	32347	30516	3,52	1799	34,567
7	34263	0,0172	32347	36254	34297	3,33	1829	36,973
8	40468	0,0414	38476	49146	43603	7,04	1784	89,155
9	67276	0,0271	59379	135885	91826	23,29	1497	58,264
10	>200000	0,0014	196980	>200000	>200000	3,48	217	3,094

TIC:	0,1876	ng/uL
TIM:	0,0261	nmole/L
Total Conc.:	0,2285	ng/uL
GQN:	2,4	
Threshold:	50000	

```
Sample Peak Width (sec): 20   Sample Min Peak Height: 50    Sample Baseline V to V?: Y    Sample Baseline V to V pts: 3
Sample Filter: Binomial       # of Pts for Filter: 3        Sample Start Region (min): 0  Sample End Region (min): 70
Manual Baseline Start (min): 25     Manual Baseline End (min): 70
Marker Peak Width (sec): 8    Marker Min Peak Height: 500   Marker Baseline V to V?: N    Marker Baseline V to V pts: 3
Lower Marker Selection: First Peak > 500 RFU                Upper Marker Selection: Last Peak > 500 RFU
Ladder Size (bp)1, 1300, 10000, 17700, 21000, 23000, 42000, 50000, 165500
Quantification Using: Ladder          Final Concentration (ng/uL): 0,0200          Dilution Factor: 10,0
Min. RFU for Data Processing: 1      Size Threshold (b.p.): 50000
```

 Copyright 2015 Advanced Analytical Technologies, Inc.

Sample: 3358.B.3
Well Location: B5
Created: 04-Dec-17 2:28 PM

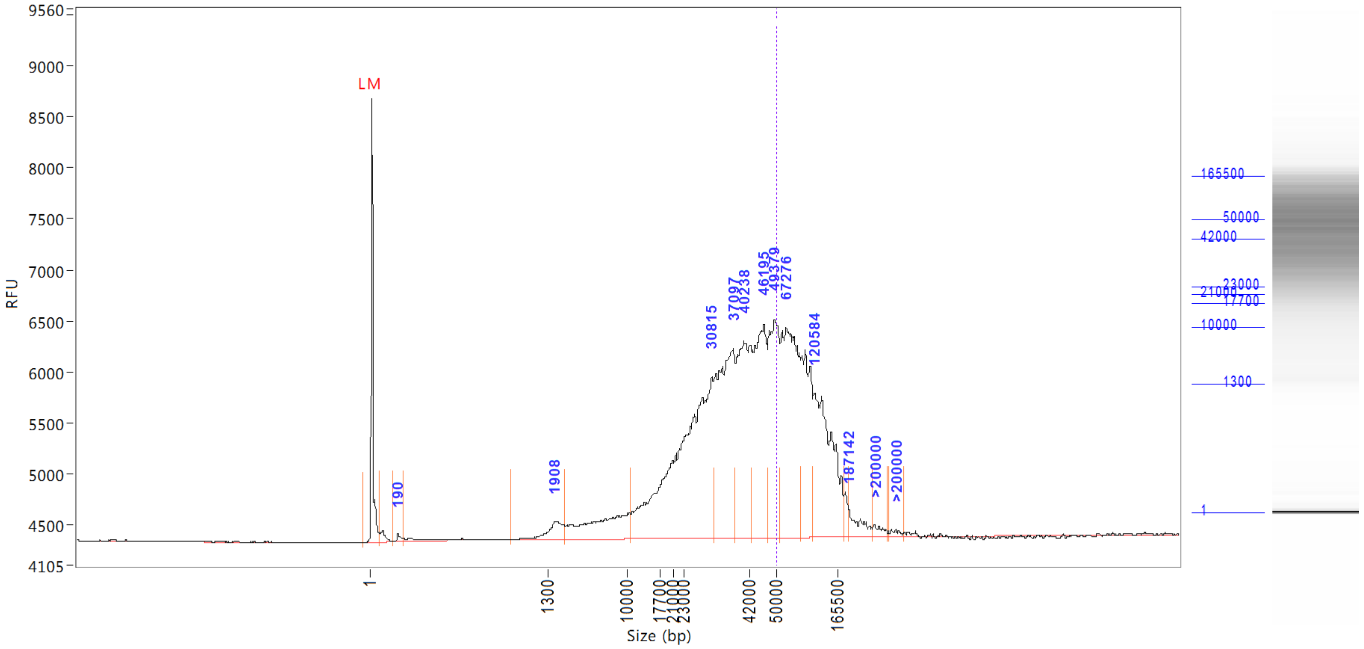

Peak	Size (bp)	Conc. (ng/uL)	From (bp)	To (bp)	Avg. Size (bp)	CV%	RFU	Corr. Peak Area
1	1 (LM)	0,0013	0	55	4	338,24	4357	27,211
2	190	0,0005	164	240	202	8,59	78	1,127
3	1908	0,0032	1023	3006	1875	31,09	178	6,764
4	30815	0,0508	10607	31505	23329	23,44	1579	109,034
5	37097	0,0261	31505	37787	34716	5,28	1858	56,127
6	40238	0,0223	37787	42700	40278	3,55	1926	47,835
7	46195	0,0205	42700	47282	45011	2,95	2099	44,021
8	49379	0,0171	47282	55430	49633	4,10	2139	36,600
9	67276	0,0274	55430	96398	75396	15,68	2054	58,849
10	120584	0,0192	119597	178780	143680	11,17	1420	41,182
11	187142	0,0020	186650	>200000	>200000	6,24	263	4,247
12	>200000	0,0007	>200000	>200000	>200000	3,34	122	1,557
13	>200000	0,0004	>200000	>200000	>200000	2,59	80	0,784

TIC:	0,1901	ng/uL
TIM:	0,0149	nmole/L
Total Conc.:	0,2148	ng/uL

GQN:	3,2
Threshold:	50000

Sample Peak Width (sec): 20   Sample Min Peak Height: 50   Sample Baseline V to V?: Y   Sample Baseline V to V pts: 3
Sample Filter: Binomial   # of Pts for Filter: 3   Sample Start Region (min): 0   Sample End Region (min): 70
Manual Baseline Start (min): 25   Manual Baseline End (min): 70
Marker Peak Width (sec): 8   Marker Min Peak Height: 500   Marker Baseline V to V?: N   Marker Baseline V to V pts: 3
Lower Marker Selection: First Peak > 500 RFU   Upper Marker Selection: Last Peak > 500 RFU
Ladder Size (bp)1, 1300, 10000, 17700, 21000, 23000, 42000, 50000, 165500
Quantification Using: Ladder   Final Concentration (ng/uL): 0,0200   Dilution Factor: 10,0
Min. RFU for Data Processing: 1   Size Threshold (b.p.): 50000

 Copyright 2015 Advanced Analytical Technologies, Inc.

Sample: 3358.B.4
Well Location: B6
Created: 04-Dec-17 2:28 PM

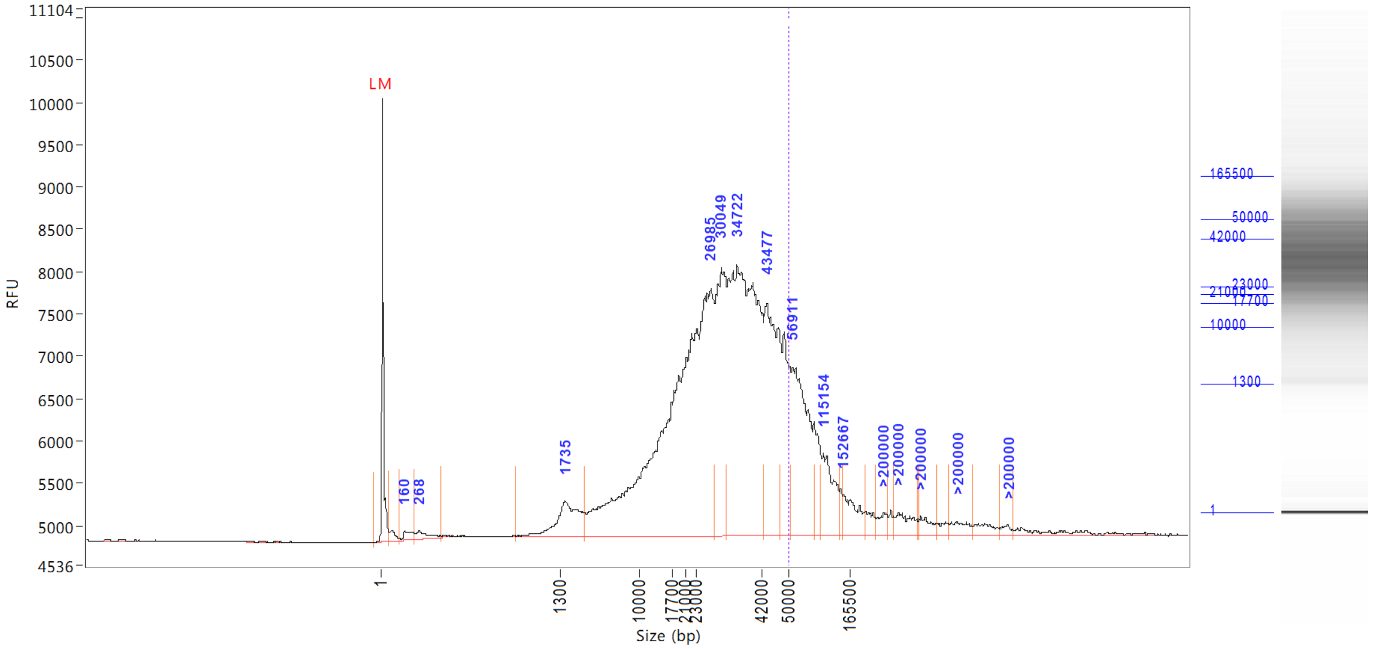

Peak	Size (bp)	Conc. (ng/uL)	From (bp)	To (bp)	Avg. Size (bp)	CV%	RFU	Corr. Peak Area
1	1 (LM)	0,0013	0	51	3	445,22	5240	32,094
2	160	0,0014	131	241	190	14,77	112	3,569
3	268	0,0014	241	428	309	15,89	92	3,609
4	1735	0,0090	977	4018	2185	41,25	411	22,895
5	26985	0,1048	4018	28057	18339	36,11	2918	265,426
6	30049	0,0243	28057	31811	29955	3,65	3173	61,555
7	34722	0,0649	31811	42545	37013	8,37	3195	164,383
8	43477	0,0247	42545	47515	44950	3,24	2751	62,418
9	56911	0,0224	51975	98372	73010	18,25	1984	56,820
10	115154	0,0075	110712	145757	126214	8,02	972	18,868
11	152667	0,0041	152667	195012	171680	7,16	481	10,262
12	>200000	0,0014	>200000	>200000	>200000	2,86	262	3,424
13	>200000	0,0024	>200000	>200000	>200000	4,72	273	6,019
14	>200000	0,0013	>200000	>200000	>200000	2,96	223	3,337
15	>200000	0,0014	>200000	>200000	>200000	3,27	167	3,481
16	>200000	0,0005	>200000	>200000	>200000	1,46	120	1,307

TIC:	0,2715	ng/uL
TIM:	0,0418	nmole/L
Total Conc.:	0,2936	ng/uL

GQN:	1,8
Threshold:	50000

Sample Peak Width (sec): 20 Sample Min Peak Height: 50 Sample Baseline V to V?: Y Sample Baseline V to V pts: 3
Sample Filter: Binomial # of Pts for Filter: 3 Sample Start Region (min): 0 Sample End Region (min): 70
Manual Baseline Start (min): 25 Manual Baseline End (min): 70
Marker Peak Width (sec): 8 Marker Min Peak Height: 500 Marker Baseline V to V?: N Marker Baseline V to V pts: 3
Lower Marker Selection: First Peak > 500 RFU Upper Marker Selection: Last Peak > 500 RFU
Ladder Size (bp)1, 1300, 10000, 17700, 21000, 23000, 42000, 50000, 165500
Quantification Using: Ladder Final Concentration (ng/uL): 0,0200 Dilution Factor: 10,0
Min. RFU for Data Processing: 1 Size Threshold (b.p.): 50000

 Copyright 2015 Advanced Analytical Technologies, Inc.

Sample: Ladder
Well Location: B12
Created: 04-Dec-17 2:28 PM
Fit Type: Point to Point

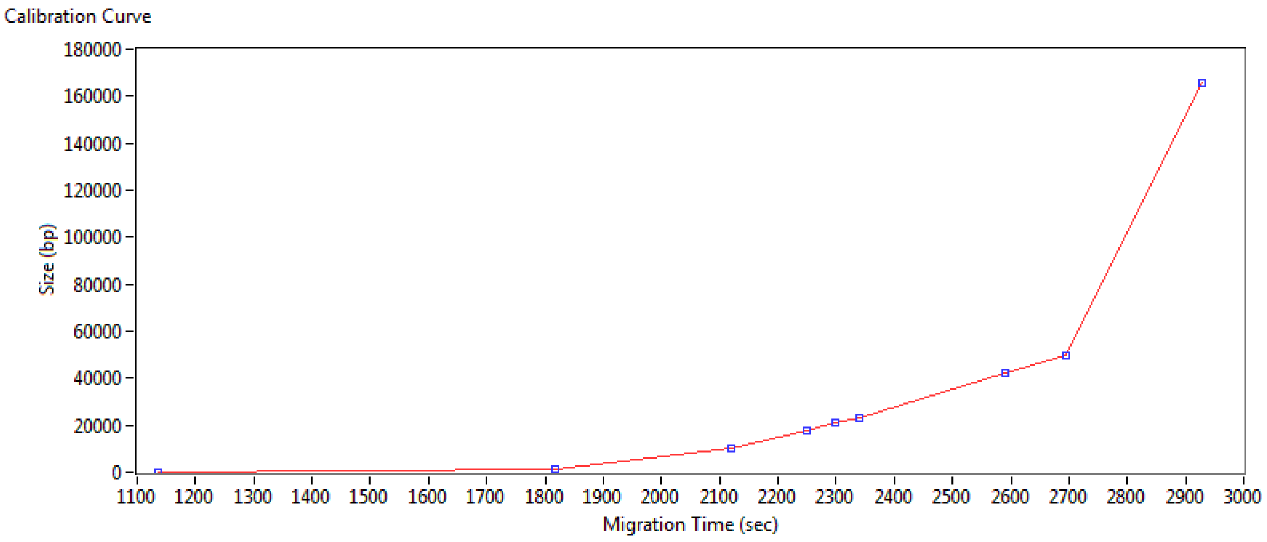

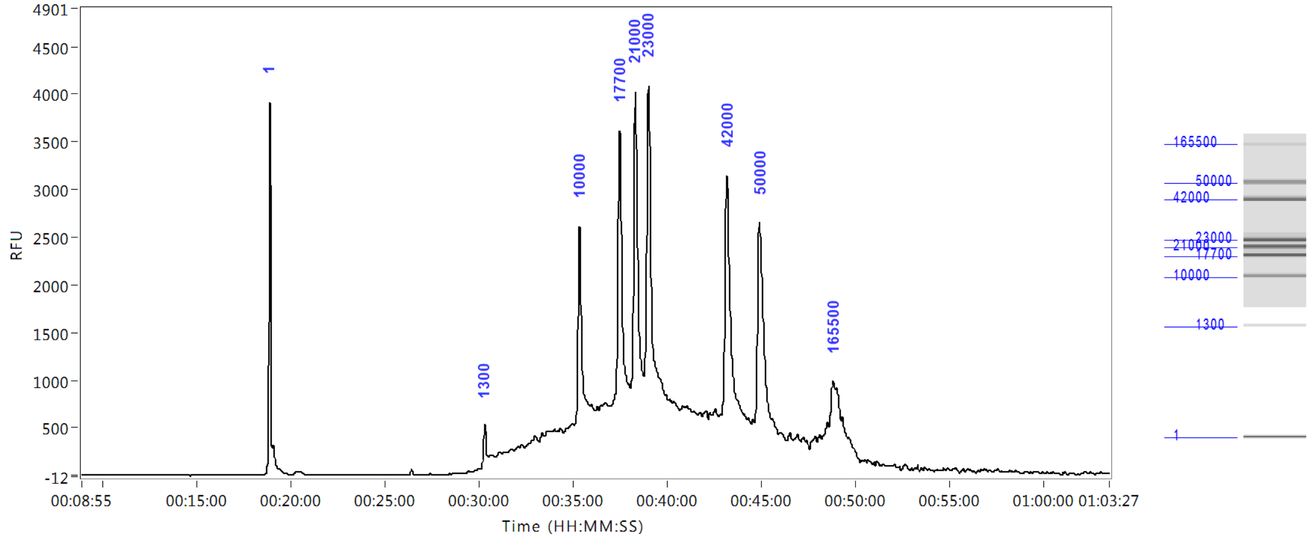

 Copyright 2015 Advanced Analytical Technologies, Inc.
